# A chromosome-level reference genome of *Ensete glaucum* gives insight into diversity and chromosomal and repetitive sequence evolution in the Musaceae

**DOI:** 10.1093/gigascience/giac027

**Published:** 2022-04-30

**Authors:** Ziwei Wang, Mathieu Rouard, Manosh Kumar Biswas, Gaetan Droc, Dongli Cui, Nicolas Roux, Franc-Christophe Baurens, Xue-Jun Ge, Trude Schwarzacher, Pat (J S) Heslop-Harrison, Qing Liu

**Affiliations:** Key Laboratory of Plant Resources Conservation and Sustainable Utilization/Guangdong Provincial Key Laboratory of Applied Botany, South China Botanical Garden, Chinese Academy of Sciences, Guangzhou 510650, China; Center of Conservation Biology, Core Botanical Gardens, Chinese Academy of Sciences, Guangzhou 510650, China; College of Life Sciences, University of the Chinese Academy of Sciences, Beijing 100049, China; Bioversity International, Parc Scientifique Agropolis II, 34397 Montpellier Cedex 5, France; French Institute of Bioinformatics (IFB) - South Green Bioinformatics Platform, Alliance Bioversity and CIAT, CIRAD, INRAE, IRD, F-34398 Montpellier, France; Department of Genetics and Genome Biology, University of Leicester, Leicester LE1 7RH, UK; French Institute of Bioinformatics (IFB) - South Green Bioinformatics Platform, Alliance Bioversity and CIAT, CIRAD, INRAE, IRD, F-34398 Montpellier, France; CIRAD, UMR AGAP Institut, F-34398 Montpellier, France; UMR AGAP Institut, Univ Montpellier, CIRAD, INRAE, Institut Agro, F-34398 Montpellier, France; Key Laboratory of Plant Resources Conservation and Sustainable Utilization/Guangdong Provincial Key Laboratory of Applied Botany, South China Botanical Garden, Chinese Academy of Sciences, Guangzhou 510650, China; Center of Conservation Biology, Core Botanical Gardens, Chinese Academy of Sciences, Guangzhou 510650, China; College of Life Sciences, University of the Chinese Academy of Sciences, Beijing 100049, China; Bioversity International, Parc Scientifique Agropolis II, 34397 Montpellier Cedex 5, France; CIRAD, UMR AGAP Institut, F-34398 Montpellier, France; UMR AGAP Institut, Univ Montpellier, CIRAD, INRAE, Institut Agro, F-34398 Montpellier, France; Key Laboratory of Plant Resources Conservation and Sustainable Utilization/Guangdong Provincial Key Laboratory of Applied Botany, South China Botanical Garden, Chinese Academy of Sciences, Guangzhou 510650, China; Center of Conservation Biology, Core Botanical Gardens, Chinese Academy of Sciences, Guangzhou 510650, China; Key Laboratory of Plant Resources Conservation and Sustainable Utilization/Guangdong Provincial Key Laboratory of Applied Botany, South China Botanical Garden, Chinese Academy of Sciences, Guangzhou 510650, China; Department of Genetics and Genome Biology, University of Leicester, Leicester LE1 7RH, UK; Key Laboratory of Plant Resources Conservation and Sustainable Utilization/Guangdong Provincial Key Laboratory of Applied Botany, South China Botanical Garden, Chinese Academy of Sciences, Guangzhou 510650, China; Department of Genetics and Genome Biology, University of Leicester, Leicester LE1 7RH, UK; Key Laboratory of Plant Resources Conservation and Sustainable Utilization/Guangdong Provincial Key Laboratory of Applied Botany, South China Botanical Garden, Chinese Academy of Sciences, Guangzhou 510650, China; Center of Conservation Biology, Core Botanical Gardens, Chinese Academy of Sciences, Guangzhou 510650, China

**Keywords:** centromeres, chromosome-scale assembly, Ensete glaucum, Musaceae evolution, Nanopore, pangenome, repetitive DNA, retrotransposons, synteny, translocations

## Abstract

**Background:**

*Ensete glaucum* (2*n* = 2*x* = 18) is a giant herbaceous monocotyledonous plant in the small Musaceae family along with banana (*Musa*). A high-quality reference genome sequence assembly of *E. glaucum* is a resource for functional and evolutionary studies of *Ensete*, Musaceae, and the Zingiberales.

**Findings:**

Using Oxford Nanopore Technologies, chromosome conformation capture (Hi-C), Illumina and RNA survey sequence, supported by molecular cytogenetics, we report a high-quality 481.5 Mb genome assembly with 9 pseudo-chromosomes and 36,836 genes. A total of 55% of the genome is composed of repetitive sequences with predominantly LTR-retroelements (37%) and DNA transposons (7%). The single 5S ribosomal DNA locus had an exceptionally long monomer length of 1,056 bp, more than twice that of the monomers at multiple loci in *Musa*. A tandemly repeated satellite (1.1% of the genome, with no similar sequence in *Musa*) was present around all centromeres, together with a few copies of a long interspersed nuclear element (LINE) retroelement. The assembly enabled us to characterize in detail the chromosomal rearrangements occurring between *E. glaucum* and the x = 11 species of *Musa*. One *E. glaucum* chromosome has the same gene content as *Musa acuminata*, while others show multiple, complex, but clearly defined evolutionary rearrangements in the change between x= 9 and 11.

**Conclusions:**

The advance towards a Musaceae pangenome including *E. glaucum*, tolerant of extreme environments, makes a complete set of gene alleles, copy number variation, and a reference for structural variation available for crop breeding and understanding environmental responses. The chromosome-scale genome assembly shows the nature of chromosomal fusion and translocation events during speciation, and features of rapid repetitive DNA change in terms of copy number, sequence, and genomic location, critical to understanding its role in diversity and evolution.

## Background

The genus *Ensete* Bruce ex Horaninow (Musaceae) includes 10 species of giant, herbaceous monocotyledonous plants, native to tropical Africa and Asia [1]. Among them, the African species *Ensete ventricosum* (Welw.) Cheesman (enset) is an important food crop for >20 million people in Ethiopia [2]. Its sister genus *Musa*, grown throughout the tropics for food and fibre, includes diploid species, triploids, and hybrids of *Musa acuminata* and *Musa balbisiana*, with banana cultivars. Sisters to the grasses (Poales) and palms (Arecales) in monocots, both *Ensete* and *Musa*, along with a third genus *Musella*, belong to Musaceae in the order Zingiberales (gingers and bananas) [3]. Following rapid diversification of the Zingiberales at the Cretaceous/Tertiary boundary (>65 million years ago [Mya]) the crown node age of the Musaceae family soon appears, with the *Musa* genus diverging from *Ensete* and *Musella* ∼40 Mya [4, 5].

**Figure 1:**
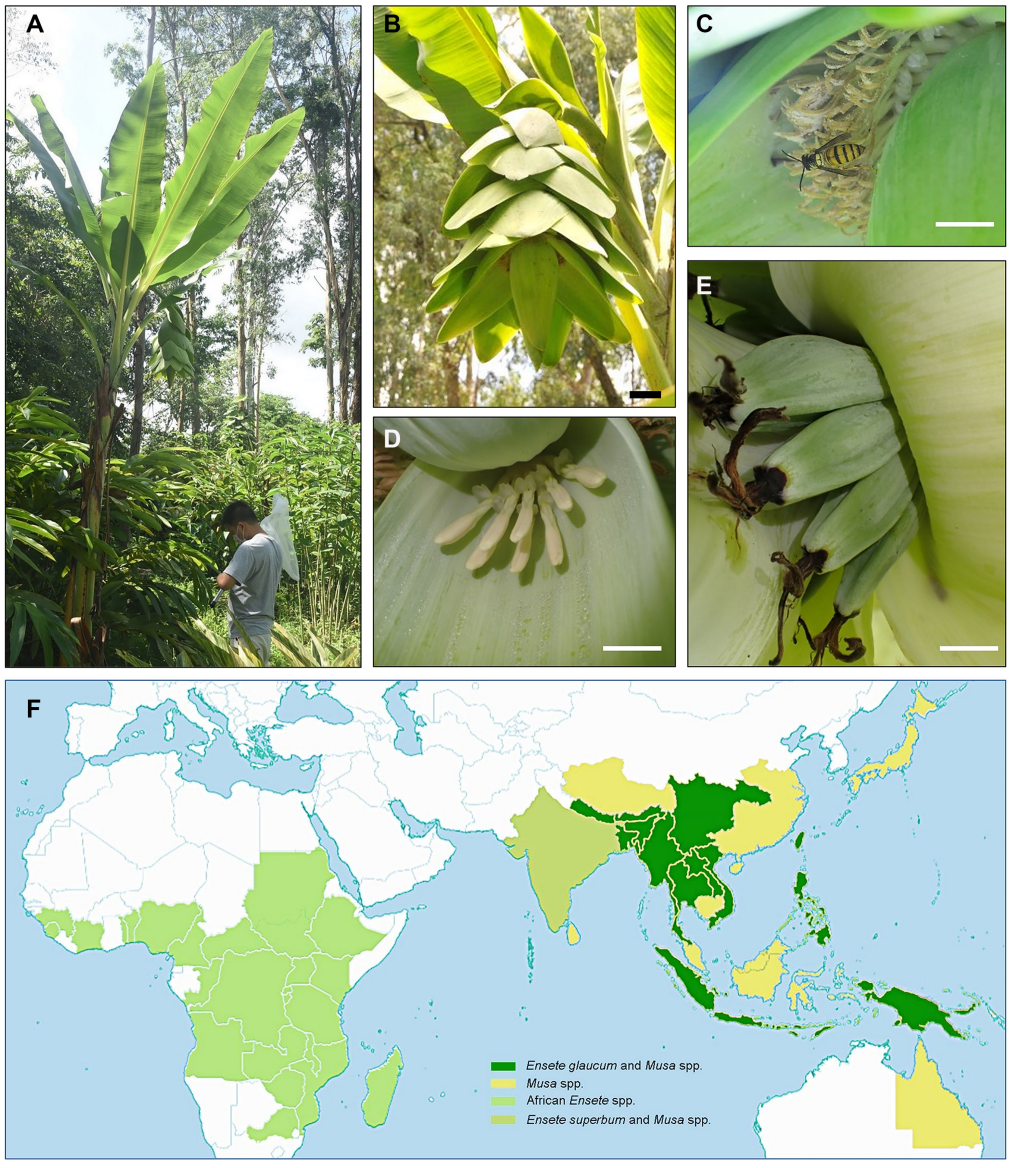
*Ensete glaucum* plant morphology and distribution map. (A) *E. glaucum* in South China Botanical Garden, Chinese Academy of Sciences. The pseudo-stem of this plant is ∼3.5 m tall. (B) Inflorescence with male and female flowers showing bracts and flowers alternately arranged along the main axis. (C) Staminate flowers, and visiting black shield wasp (*Vespa bicolor*, Vespidae, Hymenoptera). (D) Female flowers. (E) Fruits. Bars represent 5 cm in B, 2 cm in C and D, and 1 cm in E. (F) Native distribution of *Ensete glaucum, E. superbum, Musa*, and *Ensete* species by countries or provinces (for China, India-Assam, and Australia). Musaceae are not currently native in the Americas, although *Ensete* is present in the fossil record [126]. *E. glaucum* always occurs in the same provinces as *Musa* and sometimes with other Asian *Ensete* species. Map adapted from POWO [62].

*Ensete glaucum* (Roxb.) Cheesman (NCBI: txid482298), like other species in *Ensete*, is monocarpic with a dilated and characteristically glaucous basal pseudo-stem (Fig. 1A–E), with a small number of large seeds (10 mm in diameter) in elongated, banana-like fruits, borne in hands with a terminal flower and is diploid with 2n = 2x = 18 chromosomes [1, 6–10]. *E. glaucum* is widely distributed in Asia (Fig. 1F) and has records from Burma, China, India, Indonesia, Laos, Myanmar, Vietnam, Philippine, Papua New Guinea, Thailand, and Solomon Islands [11].

Originating in the tropics and subtropics at lower elevations, most species in Musaceae lack cold acclimation. Cold stress is one of the key limitations in extending banana planting and production to higher altitudes and beyond the tropics [12]. In contrast to other Musaceae species, *Ensete glaucum* can be found above 1,000 m in the mountains of Yunnan in China, where the temperature often drops lower than 0°C, with limited rainfall in winter. As one of the most cold-resistant and perhaps the most drought-tolerant species in Musaceae, *E. glaucum* is a potential gene and germplasm resource for abiotic stress tolerance in banana breeding, likely to be required for the adaptation to a more variable and extreme climate in the future.

Whole-genome assemblies (genome sequences) are published for some species of *Musa* with pseudo-chromosome–level data [13–17] using long-molecule sequencing with an N50 of >42 Mb for *M. acuminata* [17], *M. balbisiana* [15], and *Musa schizocarpa* [13]). The assemblies and annotations are available on the Banana Genome Hub, a community website that brings together genomic data, with genome browsers, extensive search facilities, and comparison features [18]. Draft genome assemblies in *Ensete* species are limited to accessions of *E. ventricosum*, but these are with tens of thousands of contigs with N50 lengths mostly between 10,000 and 21,000 bp and no pseudo-chromosome assignments [19, 20]. Effective analysis, introduction, and utilization of genetic resources present in wild species of *Ensete*, based around genome assemblies, are a need for banana improvement and understanding the genome evolution in Musacea. We applied Illumina, Oxford Nanopore Technologies (ONT), and chromosome conformation capture (Hi-C) sequencing to generate a high-quality chromosome-level assembly of the *Ensete glaucum* genome. We aimed to use the chromosome sequence to show the genome structure and gene composition, as well as revealing the repetitive DNA organization. The structural variations of *E. glaucum* (x = 9) were studied in a comparative context with *Musa* (x = 11) species, showing the evolutionary history of the family. The study aims to be useful in expanding the gene pool available not only to banana and enset breeder, but also for plant conservation of biodiversity in ecologically sensitive or threatened areas, and for fundamental research on chromosome and genome evolution.

## Analyses, Results, and Discussion

### *De novo* chromosome-scale genome assembly

**Figure 2:**
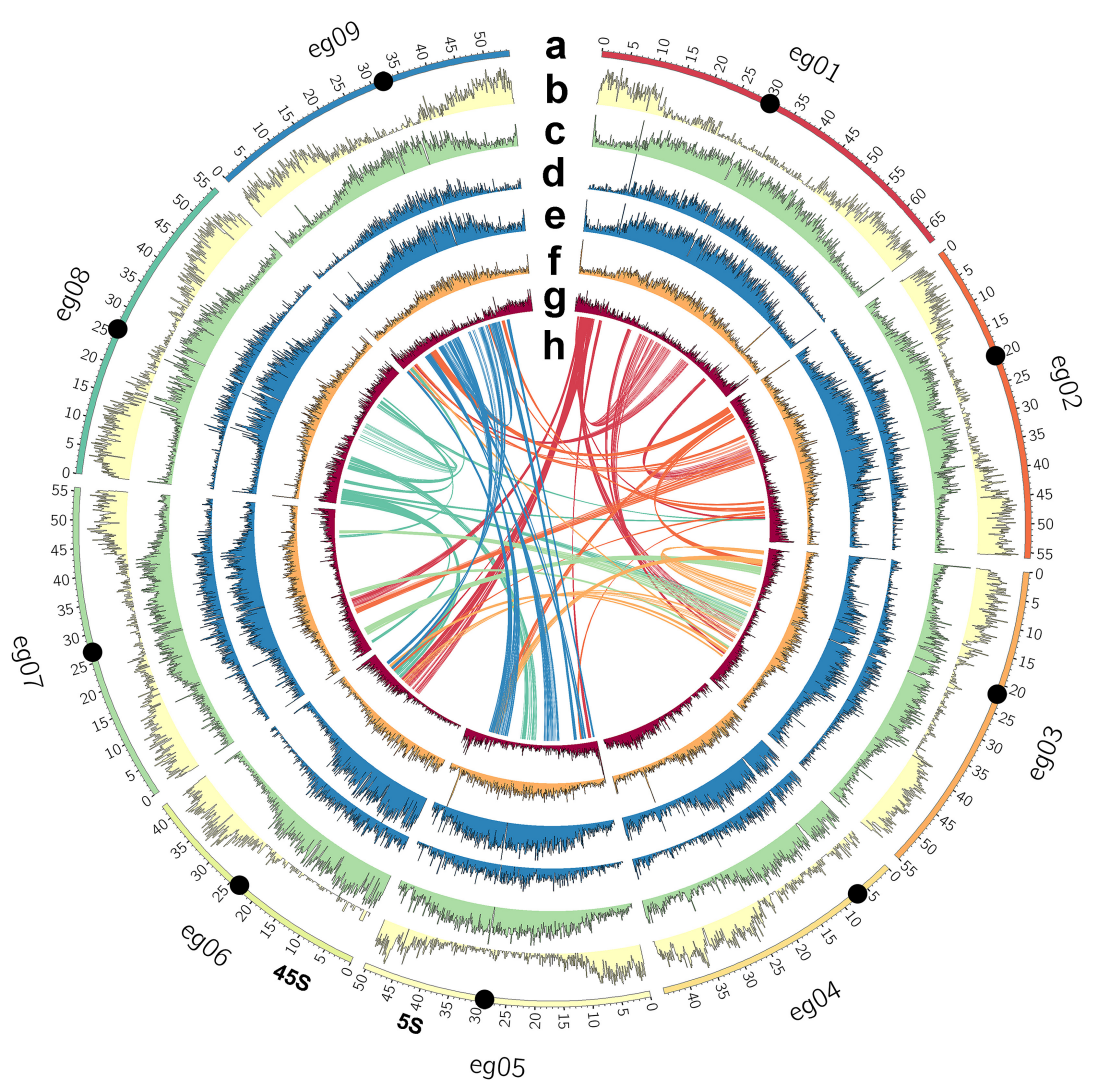
*Ensete glaucum* chromosome assembly and genome features. Circos plot of (a) The 9 pseudo-molecules (eg01 to eg09) of the EGL assembly corresponding to the 9 chromosomes. 5S and 45S rDNA loci are indicated, and centromere positions are shown by black dots; scale in Mb; (b) gene density; (c) repeat density; (d) *Copia* LTR retroelement density; (e) *Gypsy* LTR retroelement density; (f) DNA transposon density; (g) simple sequence repeat (microsatellite) density; (h) Syntenic genomic blocks, linked by curved lines (arbitrary colour) in middle of the plot.

A *de novo* chromosome-level assembly of *Ensete glaucum* was made by combining high-coverage ONT long-read sequencing, Illumina 150 bp paired-end sequences, and Hi-C chromosome conformation capture sequence data (Table 1). From the initial assembly (with N50 of 10.256 Mb, Table 2 and Supplementary Table S1), we assembled 9 pseudo-molecules, eg01–eg09 (Fig. 2 and Supplementary Table S2), corresponding to the chromosome number (2n = 18) and observed chromosome morphology.

**Table 1:** Statistics of whole-genome sequence assembly and transcriptome analysis of *Ensete glaucum* using Illumina, ONT, and Hi-C

Type	Method	No. of reads	Clean data (Gb)	Read length (bp)	Assembly coverage (×)
Genome	Illumina	245,852,534	36.88	2 × 150	74
	ONT	4,357,035	109	38,885 (N50)	220
	Hi-C	319,793,734	48	2 × 150	
Transcriptome	Illumina (Leaf)	62,410,840	94	2 × 150	
	Illumina (Root)	13,712,542	21	2 × 150	

Sizes and coverage are based on the unreplicated haploid genome (1C).

**Table 2:** Statistics of *Ensete glaucum* genome assembly and annotation

**Genome assembly**	Value
*k*-mer estimation of genome size (17-mer)	563,295,571 bp
Total contig length	495,175,598 bp
Percentage of estimated genome	87.9%
Anchored into chromosomes	481,507,213 bp
GC content	38.21%
Contigs	
Number	124
N50 length	10,255,891 bp
Longest	31,226,749 bp
Pseudo-chromosomes	
Number	9
Shortest	42,457,113 bp
Complete BUSCOs of genome	98.3%
**RepeatMasker repetitive DNA**	
Transposable elements	
LTR retroelements	37.20%
*Copia*	17.64%
*Gypsy*	19.25%
LINEs	0.77%
Class II DNA transposons	7.18%
Unclassified dispersed repeats	8.74%
Simple repeats and low complexity	1.13%
Total repeats (RepeatMasker)	55.02%
**Tandem repeat content**	
45S rDNA	1.21%
5S rDNA	0.08%
Egcen satellite	1.32%
Microsatellites (<8 bp motif)	0.59%
**Protein-coding genes**	
Number	36,836
Average number of exons per gene	4.86
Average exon length per gene	1,114 bp
Average intron length	2,816 bp
Average length of predicted proteins	371 aa
Complete BUSCOs of predicted genes	94.7%
**Functional annotation**	
NR	31,599 (85.78%)
InterPro	30,160 (81.88%)
GO	24,436 (66.34%)
KO	11,192 (30.38%)
Total	31,804 (86.34%)

RepeatMasker did not identify satellite sequences. 5S and 45S rDNA and the centromeric sequence Egcen were identified manually in assemblies and the abundance measured in raw read data; microsatellite abundance was calculated from the assemblies (see Supplementary Tables S1, S2, S3, S10, S12, and S14).

A Hi-C/ONT-only assembly was constructed first, by using an OLC (overlap layout-consensus)/string graph method with corrected reads. Contigs were refined using Illumina short reads, and after discarding redundant contigs, the final genome assembly was 481 Mb long, with 9 pseudo-chromosomes between 42,457,113 and 67,484,389 bp long. BUSCO analysis [21] was used to assess the assembly in “genome” mode showing 98.3% complete single and duplicated Embryophyta core gene sets from the embryophyta_odb10 database (Table 2, Supplementary Table S3a): of the 1,614 genes tested for, 1,526 are complete and single-copy BUSCOs (S), 61 are complete and duplicated BUSCOs (D), 12 are fragmented BUSCOs (F), 15 are missing BUSCOs (M). Few genes were fragmented or missing. *E. glaucum* chromosome designations were chosen to follow major regions of synteny with *M. acuminata* chromosomes [16, 17].

### Genome size, heterozygosity, and organization

The contig-level assembly size is 495,175,598 bp, and 97.2% of these contigs are anchored to 9 pseudo-chromosomes after Hi-C scaffolding, resulting in a 481,507,213-bp final chromosome-level genome assembly. Some arrays of tandem repeats, including the ribosomal DNA (rDNA) (see below) and telomeres, were collapsed and chromosome termini were not fully assembled. Approximately 55% of the assembled genome was estimated to be repeat sequences (RepeatMasker; Table 2). The genome size was estimated as 563,295,571 bp (highest 17-mer peak frequency). Presumably because of sensitivity of parameters to the evolutionary whole-genome duplications (WGDs) (Fig. 1, centre) and more recent duplications, slightly higher estimates were made by findGSE software (*k* = 21: 588,939,614 bp; range from *k* = 17 to 25, 582–591 Mb), and lower estimates by GenomeScope (468,990,370 bp for *k* = 21; or 407,601,233 bp for *k* = 17, Supplementary Fig. S1). MGSE [22] gave a mean coverage of reference regions of 62.948-fold and median coverage of 75.00-fold,, corresponding to genome size estimates of 587,786,744 and 493,333,333 bp. The total genome size of *E. glaucum* (x = 9) is similar to that of the x = 11 *Musa* species (see [17]) using sequencing methods, and to estimates of both genera by flow cytometry [23].

The heterozygosity rate of *E. glaucum* was 0.164% (Supplementary Fig. S1 estimated with *k* = 21 using GenomeScope). Heterozygosity in plants is influenced by mating systems and pollination [24], life span, habitat fragmentation, and cultivation [25]. Relatively little is known about the breeding system and pollination of *Ensete* species (see [26]), although we observed insects (including the hornet *Vespa bicolor*, a widespread pollinator in southern China) visiting flowers (Fig. 1C). Our low level of heterozygosity is within the range found in individual plants in populations of *M. acuminata* ssp. *banksii* (0.02–0.34% in 24 individuals [27]; and 0.13–0.23% [28]), and in other wild monocotyledonous species including 2 (most likely self-pollinating) diploid oat species (0.07% heterozygosity in *Avena atlantica* and 0.12% *Avena eriantha* [29]); it is, however, low compared to other species (e.g., walnut, *Juglans nigra* 1.0% [30]; *Nyssa sinensis* 0.87% [31]) and, in particular, many *Musa* species, some with known hybrid genome composition [28]. The low value seen in species including *E. glaucum* here is consistent with frequent self-pollination and inbreeding, or a population bottleneck of this monocarpic tropical plant [32].

Genes were unevenly distributed along chromosomes (Fig. 2 circle b), and generally depleted in broad centromeric regions; few genes were found on the short arm of the more acrocentric chromosome eg04 and the nucleolar organizing region (NOR) bearing chromosome arm of eg06 (see rDNA below). The centromeric, gene-poor regions are rich in repeats (Fig. 2 circle c) and transposable elements (TEs) (*Copia* and *Gypsy* long terminal repeat [LTR] retroelements, Fig. 2 circles d, e, and, less markedly, DNA transposons, Fig. 2 circle f), as observed in many species (including *Musa* [14, 15]).

**Figure 3:**
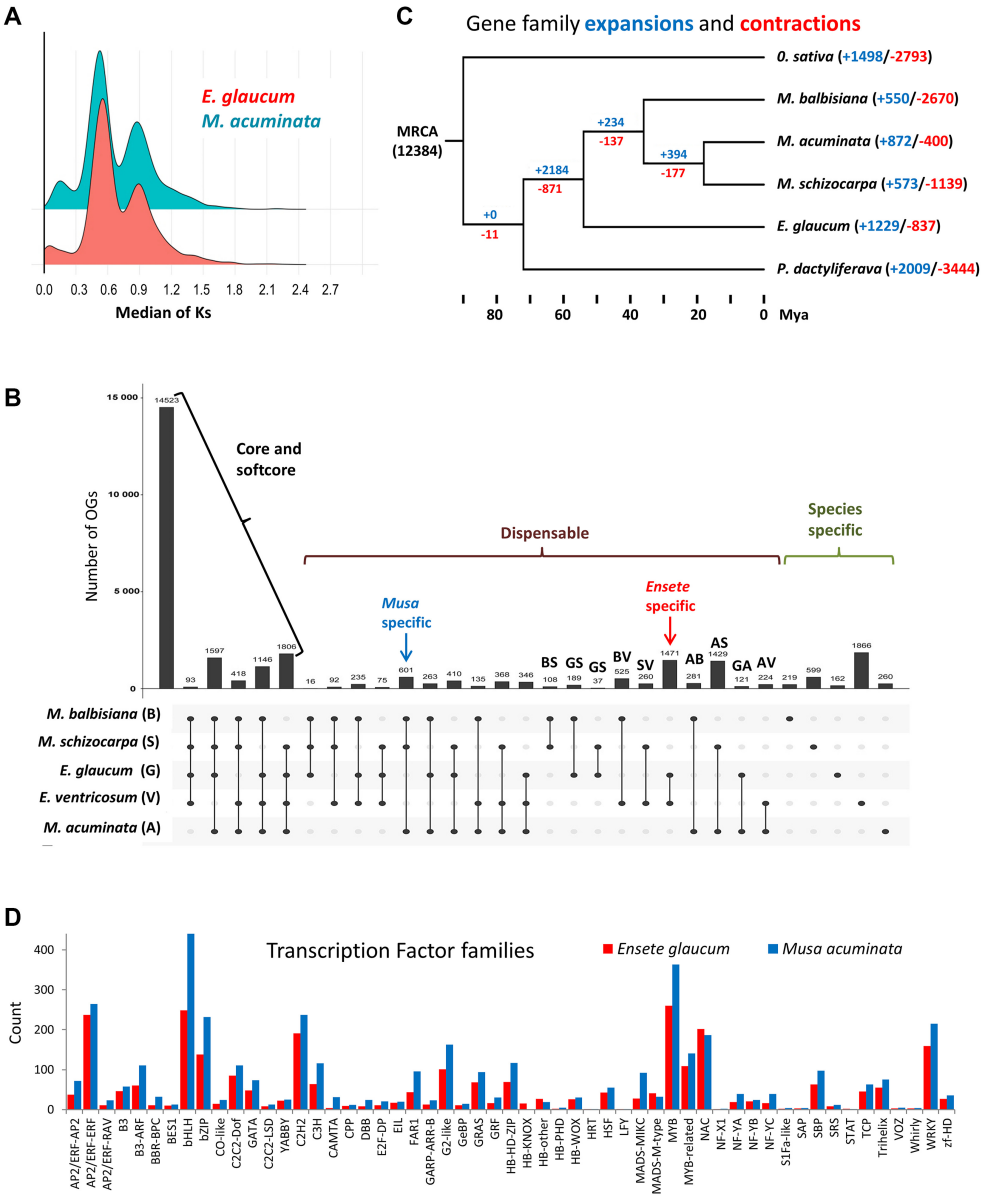
Gene family evolution and conservation. (A) The synonymous substitutions (*K*s) frequency density distributions of orthologs within EGL or MAC, whose peaks indicate whole-genome duplications (WGDs). (B) Intersection diagram showing the distribution of shared orthogroups (OGs) (≥2 sequences per OG) among *Musa* and *Ensete* genomes. E: *E. glaucum*; V: *E. ventricosum*; A: *M. acuminata*; B: *M. balbisiana*; S: *M. schizocarpa*. (C) Gene family expansion and contraction with a phylogenetic tree showing timeline of divergence of monocot species. MRCA: most recent common ancestor. Numbers denote the gene family expansion (red) and contraction (blue). (D) Histogram of the comparative abundance (number of genes) of transcription factors between *M. acuminata* and *E. glaucum*.

The Ks (the synonymous rates of substitution) between genes in paired collinearity gene groups were calculated between *E. glaucum* and *M. acuminata* to see whether they share the same 3 WGD events [14]. The 2 genomes have a nearly identical Ks density distribution (Fig. 3A), both having 2 peaks at ∼0.55 and ∼0.9. This result indicates that Musaceae share the same WGD events. The more recent peak at 0.55 most likely represents the α and β duplications, while the peaks at 0.9 may represent the more ancient γ duplication event [14]. Figure 2 (centre) links the genomic locations of paralogous gene clusters: most chromosome regions show shared relationships with 2 other chromosome regions, reflecting the α and β WGDs, as shown by D'Hont et al. [14] (their Supplementary Fig. 12).

### Gene identification

#### Genes and gene ontology

In total, 36,836 genes were predicted (BUSCO score: C: 94.7%; Supplementary Table S3B) with 31,804 (86.34%) functionally annotated with protein domain signatures and 24,436 (66.34%) associated with GO terms (Table 2; Supplementary Table S4). *E. glaucum* has a similar gene space (Supplementary Table S5) to the sequenced *Musa* species *M. acuminata* (35,264), *M. balbisiana* (35,148), *M. itinerans* (32,456), and *M. schizocarpa* (32,809). In the Musaceae (i.e., *M. acuminata, M. balbisiana, M. schizocarpa, E. ventricosum*, and *E. glaucum*), we identified a total of 29,639 orthogroups including 173,025 (88.1%) assigned genes and 23,355 (11.9%) unassigned genes (Fig. 3B and Supplementary Table S5). Between all species, the analysis showed 48% (n = 14,523) of orthogroups were shared (core or softcore genes; increasing to 66%, n = 19,583, if orthogroups missing in only 1 species are discounted as possible annotation artefacts). The analyses highlighted 5% (1,471) of orthogroups that are *Ensete* genus specific and not found in *Musa*. A total of 162 orthogroups were found only in *E. glaucum* (Fig. 3B; lower than the value for *E. ventricosum*, but the latter is a draft genome status without RNA support and with fragmented contigs with likelihood of a large number of redundant predicted genes). The predicted genes of *E. glaucum* were compared to their orthologous genes in *M. acuminata* and Ka/Ks values between orthologous pairs were calculated. Genes with Ka/Ks > 1 were under positive selection (Supplementary Table S6), and GO enrichment was used to summarize the gene functions (Supplementary Fig. S2A), showing that many regulatory biological processes have been positively selected.

#### Gene family expansion and contraction

Using *Musa* species and 2 other monocotyledonous species (in the same clade of the Commelinids), *Phoenix dactylifera* (Arecaceae) and *Oryza sativa* (Poaceae), we explored gene family expansion and contractions in *E. glaucum* (Fig. 3C and Supplementary Table S7). Among 12,384 gene families shared by the MRCA (most recent common ancestor) of these monocotyledons, there were large numbers of gene families expanding (1,498–2,184) or contracting (817–3,444) between the genomes of Musaceae, *Phoenix*, and *Oryza* (Fig. 3C), presumably reflecting substantial differences in plant form between them. Similar, although slightly lower, figures were reported between, e.g., dicotyledons as diverse as *Arabidopsis* (Brassicaceae), *Solanum* (Solanaceae), and *Cuscuta* (Convolvulaceae) [33]. Notably, though, our results show the largest expansion of gene families in the Musaceae (2,184), likely reflecting the WGD events not shared with the Poaceae or Arecaceae (see also [34] in pineapple), and we find additional expansion in *E. glaucum*. Large gene family losses were noted in *O. sativa, P. dactylifera*, and *M. balbisiana* (Supplementary Table S7).

Overall, *E. glaucum* showed enrichment of several GO biological processes (Supplementary Fig. S2B, Supplementary Table S8) compared to *Musa*. Among them, “monosaccharide transmembrane transporter” (equal top hit), “carbohydrate transmembrane transport,” and “carbohydrate transport”; and among molecular functions, “monosaccharide transmembrane transporter activity,” “sugar transmembrane transporter activity,” and “carbohydrate transmembrane transporter activity” were all included in the top 20 enrichments. The genus *Ensete* is notable for its accumulation of starch in the pseudo-stem and leaf bases, with *E. ventricosum* cultivated as a staple starchy food in East Africa [2], and perhaps this is reflected in the enrichment of certain carbohydrate transport GO terms.

#### Transcription Factors

In total, 2,637 putative transcription factor (TF) genes were identified in the *E. glaucum* assembly, representing 7% of all genes (Supplementary Table S9), which were classified by their signature DNA binding domain into 58 TF families (Fig. 3D). Similar to *M. acuminata*, the MYB (myeloblastosis) superfamily of TFs (including 260 MYB TFs plus 109 MYB-related) was the largest family, with between 140 and 210 copies of each of the bHLH, AP2/ERF, NAC, C2H2, WRKY, and bZIP families. The identification and classification of the TFs here provides a framework to explore regulatory networks in plants [35] with their target genes and to identify specific factors involved in important responses. Cenci et al. [36] analysed TFs involved in the regulation of tissue development and responses to biotic and abiotic stresses and, particularly, the NAC plant-specific gene family, while Xiao et al. [37] discuss the importance of an HLH factor involved in starch degradation during fruit ripening. *Ensete* and *Musa* differ in these characteristics, so it will be interesting to analyse differences in TFs responsible.

### Repetitive DNA analysis

#### Repeat identification

**Figure 4:**
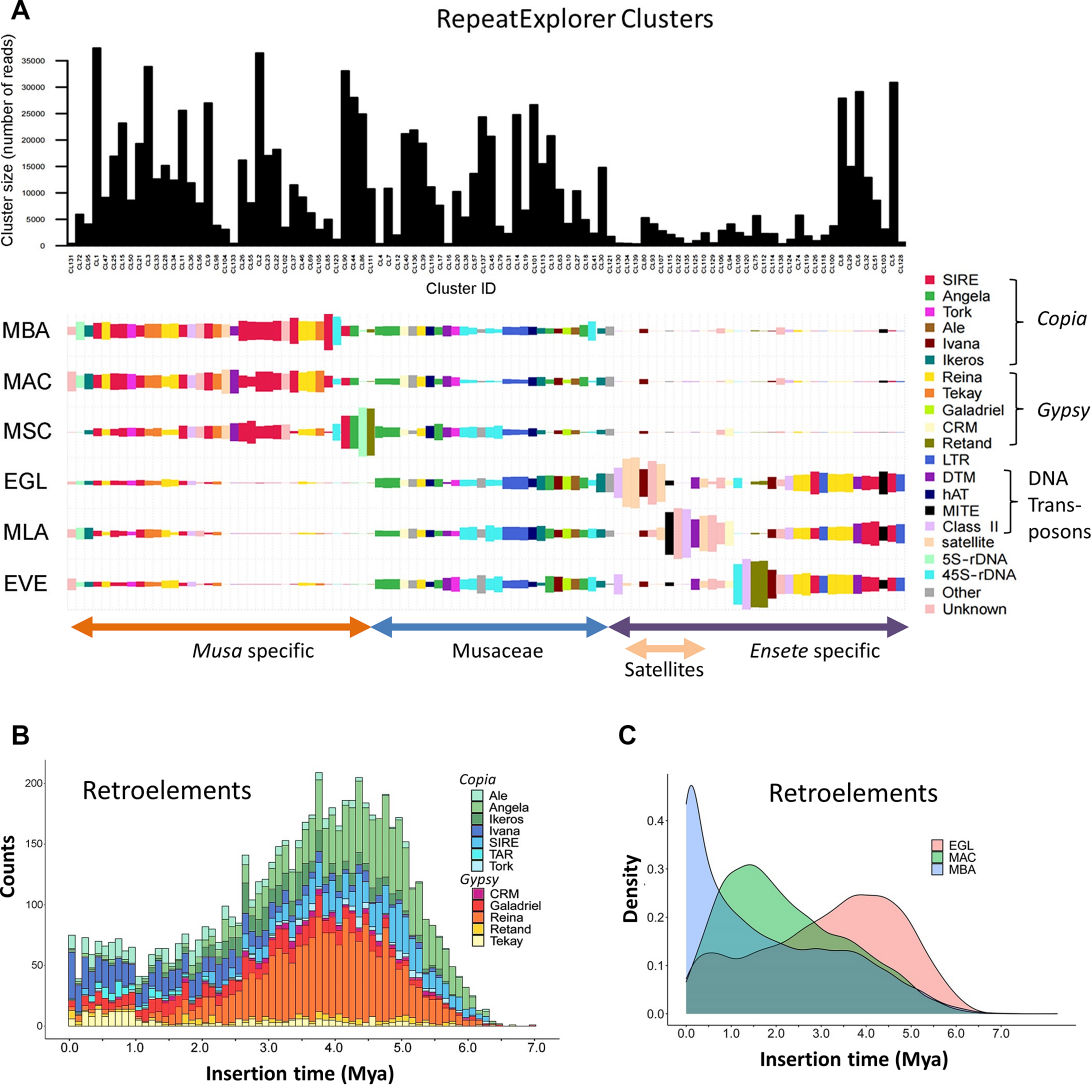
Comparative analysis of repetitive DNA in Musaceae using RepeatExplorer. (A) Bar chart showing the sizes (numbers of reads) of the most abundant individual graph-based read clusters (upper part; black bars) and display of their distribution among 6 Musaceae species (coloured rectangle sizes in lower part proportional to the number of reads in a cluster for each species, based on the annotation of the clusters). Clusters and species were sorted by using hierarchical clustering. EGL: *Ensete glaucum*; EVE: *E. ventricosum*; MAC: *Musa acuminata*; MBA: *M. balbisiana*; MLA: *Musella lasiocarpa*; MSC: *M. schizocarpa*. (B) The distribution of insertion times of LTR retroelements (members of *Copia* and *Gypsy* classes) in *E. glaucum*. (C) The ages of total LTR-retroelement insertions in *E. glaucum, M. acuminata*, and *M. balbisiana*. Mya: million years ago.

**Figure 5:**
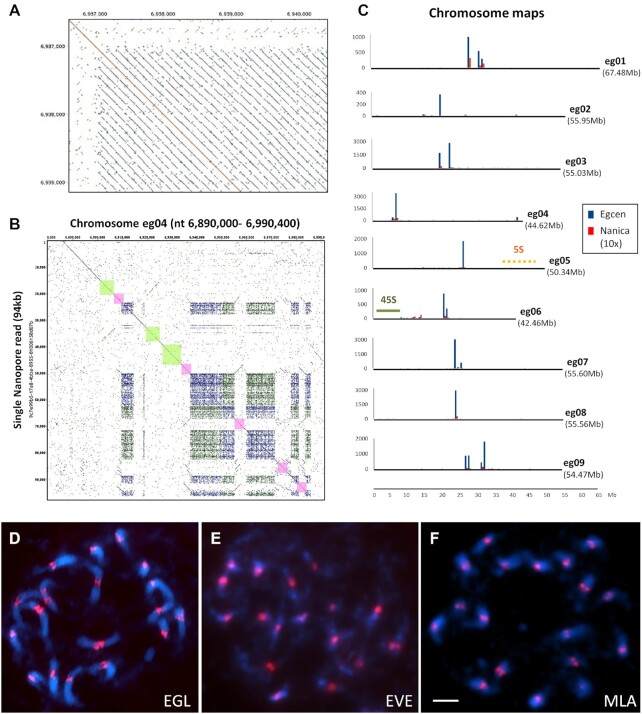
*Ensete glaucum* centromeric repeat structure. (A) Dot plot (self-comparison of sequences) showing start of a 134-bp Egcen tandem array. (B) Dot plot showing part of a chromosomes assembly (eg04) plotted against part of a single ONT read with blocks of the Egcen tandem repeat (appearing as dense rectangles at this scale) interspersed with *Nanica* elements (pink; 5 homologous copies in both orientations) and LTR retroelements (green; 2 non-homologous subfamilies). (C) Bar chart showing frequency distribution of the Egcen centromeric tandem repeat, *Nanica* transposable elements (×10 on axis), and locations of 45S and 5S rDNA along the assemblies for each pseudo-chromosome. Long Egcen arrays occur at 1 or more sites at the centromeric regions of all chromosomes. (D–F) *In situ* hybridization of Egcen probe detected by red fluorescence to cyan-fluorescing DAPI-stained chromosomes of (D) EGL, *Ensete glaucum*; (E) EVE, *E. ventricosum*; and (F) MLA, *Musella lasiocarpa*. The red Egcen signals collocate with the primary centromeric constriction on all 9 pairs of chromosomes. Bar = 5 µm.

**Figure 6:**
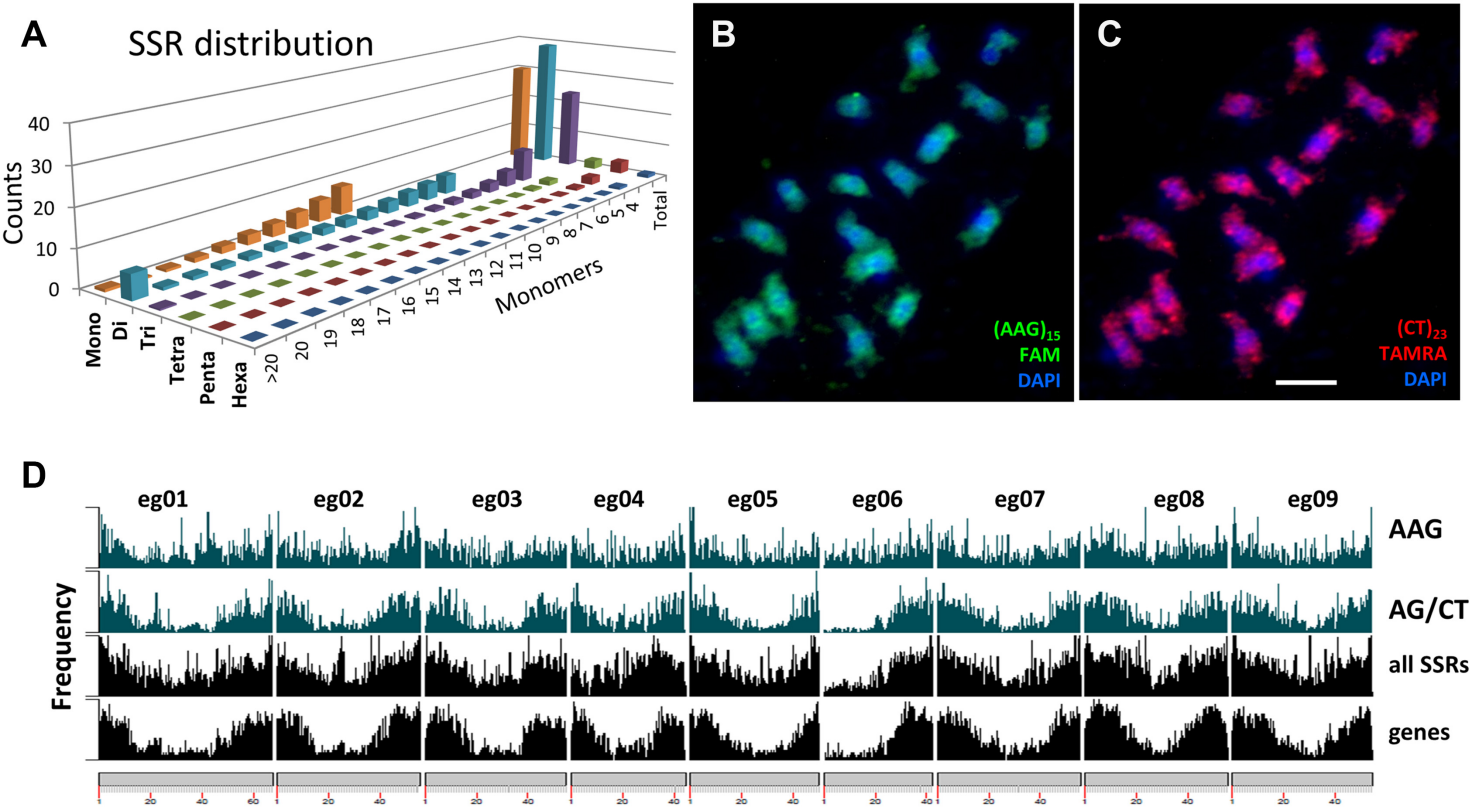
Microsatellite (SSR) distribution in *Ensete glaucum*. (A) Abundance (count) and total number of monomers of microsatellites (SSR) with motifs between 1 and 6 bp long. (B, C) *In situ* hybridization of synthetic microsatellite probes to DAPI-stained (blue) chromosomes, showing (B) AAG is relatively uniformly distributed along chromosomes compared to (C) where the greater abundance of AG/CT in distal chromosome regions is seen. (D) Abundance of AAG, AG, all microsatellites, and genes along the chromosome assemblies. In agreement with the *in situ* hybridization result, AAG is more uniformly distributed, while AG (along with genes and all the microsatellites pooled) show greater abundance in distal chromosome regions except for the arm of chromosome eg06 carrying the 45S rDNA (NOR). Bar = 5 µm.

**Figure 7:**
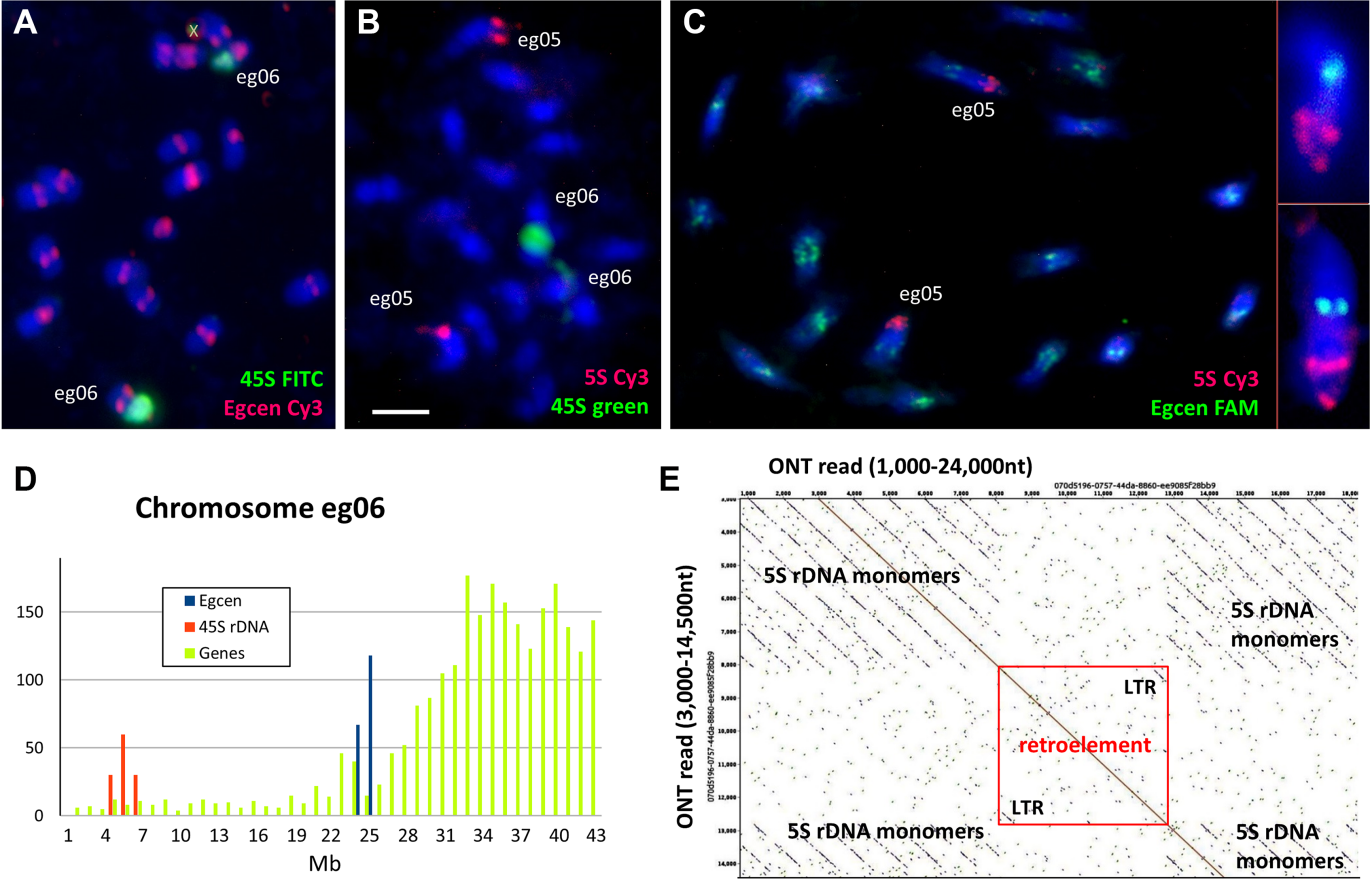
rDNA in *Ensete glaucum*. (A–C) *In situ* hybridization to chromosomes (stained blue with DAPI) showing locations of (A) the 45S rDNA (green) on 1 pair of chromosomes (eg06) while Egcen (red) is located at the centromeres of all 9 chromosome pairs. Unspecific signal is marked by x. (B) Chromosomes showing location of 45S rDNA loci (green on eg06; the 2 sites are on 2 chromosomes that are adjacent to each other and the 2 loci have fused); the 5S rDNA loci (red) are located near the end of 1 chromosome pair [eg05]). (C) The 5S rDNA (red on eg05) in a more dispersed pattern with Egcen (green) at all 9 pairs of centromeres; inset shows 5S rDNA chromosomes at higher magnification. The 5S rDNA signal is dispersed over a longer region of the chromosome, while the 45S rDNA locus is dense and occupies much of the chromosome arm. Bar = 5 µm. (D) Histogram showing density of genes (light green), Egcen (blue), and 45S rDNA copies (red) on chromosome eg06. The arm carrying the 45S rDNA is depleted in protein-coding genes. (E) Part of a single ONT read covering 24 kb spanning part of the 5S rDNA array. The unusually long 1,056-bp tandemly repeated 5S rDNA monomers (14 copies) are interrupted by an LTR retroelement. LTRs, with no homology to the 5S rDNA, are seen (bottom left and top right) in the red box.

A range of different programs were applied for repeat analysis, and, as has been considered previously [38], there were differences in the repeats identified between approaches, and small changes in parameters and reference sequences give substantial changes. Repeated elements in the genome assembly were identified by RepeatMasker (Table 2 and Supplementary Table S10) and amounted to 55% of the genome assembly, the same range as other plant species with similar DNA amount and, particularly, the genus *Musa* [14, 15]. For assembly-free identification of repeats, we used RepeatExplorer [39] to generate graph-based clusters of similar sequence fragments: Illumina sequence reads are available from 6 Musaceae species, allowing assembly-free comparisons (Supplementary Table S11 and Supplementary Fig. S3); while there was a little more variation in proportion of reads in the most abundant clusters, all had between 33% and 46% in the top clusters (>0.01% genomic abundance, as defined in [37]). TEs including LTR and non-LTR retroelements, and class II DNA transposons, were found (Fig. 4 and Supplementary Table S11). Microsatellites and other repeats were further characterized by mining and dot plot analysis, as well as fluorescent *in situ* hybridization (FISH) to chromosomes (Supplementary Tables S12–S14 and Figs 5–7; see below). The organization of repetitive regions in the assembly was sometimes verified by mapping individual ONT long reads to assembled repeat regions (e.g., Fig. 5A), and organization was generally confirmed, except for some long tandem arrays that seem to be collapsed in the assembly owing to high homology between repeat units. A few ONT reads were found that included reversals of tandem arrays (head-to-head or tail-to-tail junctions), potentially artefacts from both strands of the DNA molecule passing sequentially through 1 pore, and these junctions need further investigation.

Figure 4A compares the abundance and species distributions of major repeat classes in the Musaceae using the comparative genome analysis function of RepeatExplorer. All species shared many transposons and rDNA sequences (Fig. 4A, central region). However, genus-specific retroelement variants were identified in *Musa* (Fig. 4A, left) and *Ensete*-with-*Musella* (Fig. 4A, right), showing the separation of the 2 phylogenetic branches, supported by extensive divergence of the repetitive sequence subfamilies, and evolution in copy number. Notably, satellite sequences (Fig. 4A centre-right) were much more abundant and some sequences (see centromere sequence below) were present exclusively in *Ensete*.

#### Transposable elements

The most abundant class of repetitive elements were TEs, in particular LTR retroelements. The distributions of *Copia* and *Gypsy* LTR retroelements along assembled pseudo-chromosomes (Fig. 2 circles d and e) show greater abundance in proximal chromosome regions. Approximately equal numbers of *Copia* and *Gypsy* elements (18 and 19% of the genome assembly, respectively, Table 2) were found. This result contrasts with *M. acuminata*, where *Copia* elements were considerably more frequent (29%) compared to *Gypsy* elements (11% [14]; Supplementary Table S11). The relative change in proportions of the 2 element families, while the overall abundance remains the same, has implications for genome evolution and the expansion or contraction of retrotransposon families, which can be explored in detail using the high-quality genome sequences where the elements are neither truncated nor collapsed.

Analysis of reverse transcriptase (RT) domains identified subfamilies of LTR retroelements, with the families showing different abundances in *E. glaucum* and *M. acuminata* (Supplementary Fig. S4). Insertion times of LTR retroelement subfamilies (Fig. 4B and C and Supplementary Fig. S5) were calculated based on LTR divergence for *E. glaucum* and recalculated for *Musa* to allow for identical software settings (see Material, Methods, and Validation). In *E. glaucum*, both *Copia* and *Gypsy* families show relatively constant activity over the past 2.5 Mya, with the major peak of insertion activity 3.5–5.5 Mya (Fig. 4B and C), corresponding to the half-life of LTR-elements [14]. The dynamic amplification of these elements is emphasized by individual subfamilies having bursts of amplification (Fig. 4B for *E. glaucum* and Supplementary Fig. S5 for *Musa*), with rounds of expansion of different elements. As shown by Wang et al. [15], *M. balbisiana* has the most extensive LTR activity in the past 500,000 years, and *M. acuminata* activity peaks ∼1.5 Mya (Fig. 4C), in both cases with greater activity of *Copia* elements (Supplementary Fig. S5), contrasting with *E. glaucum* with equal activity of both *Gypsy* and *Copia* elements, leading to a higher proportion of *Gypsy* elements within the genome of *E. glaucum* compared to *Musa* (see above, and Supplementary Tables S10 and S11). This is also evidenced by the larger number of *Musa*-specific clusters identified as *Copia* Angela or Sire elements while *Ensete* with *Musella*-specific LTRs include more *Gypsy* Reina and Retand elements (Fig. 4A). Wu et al. [40] discuss the rounds of amplification in *M. itinerans* with an amplification burst after separation from *M. acuminata* ∼5.8 Mya suggesting high turnover of the elements. The results suggest a burst of retroelement amplification (the older ones), sometime after the split of *Musa* and *Ensete*, and again more recently, perhaps after *E. glaucum* split from other *Ensete* species.

#### Tandem (satellite) repeats and centromeric sequences

**Figure 8:**
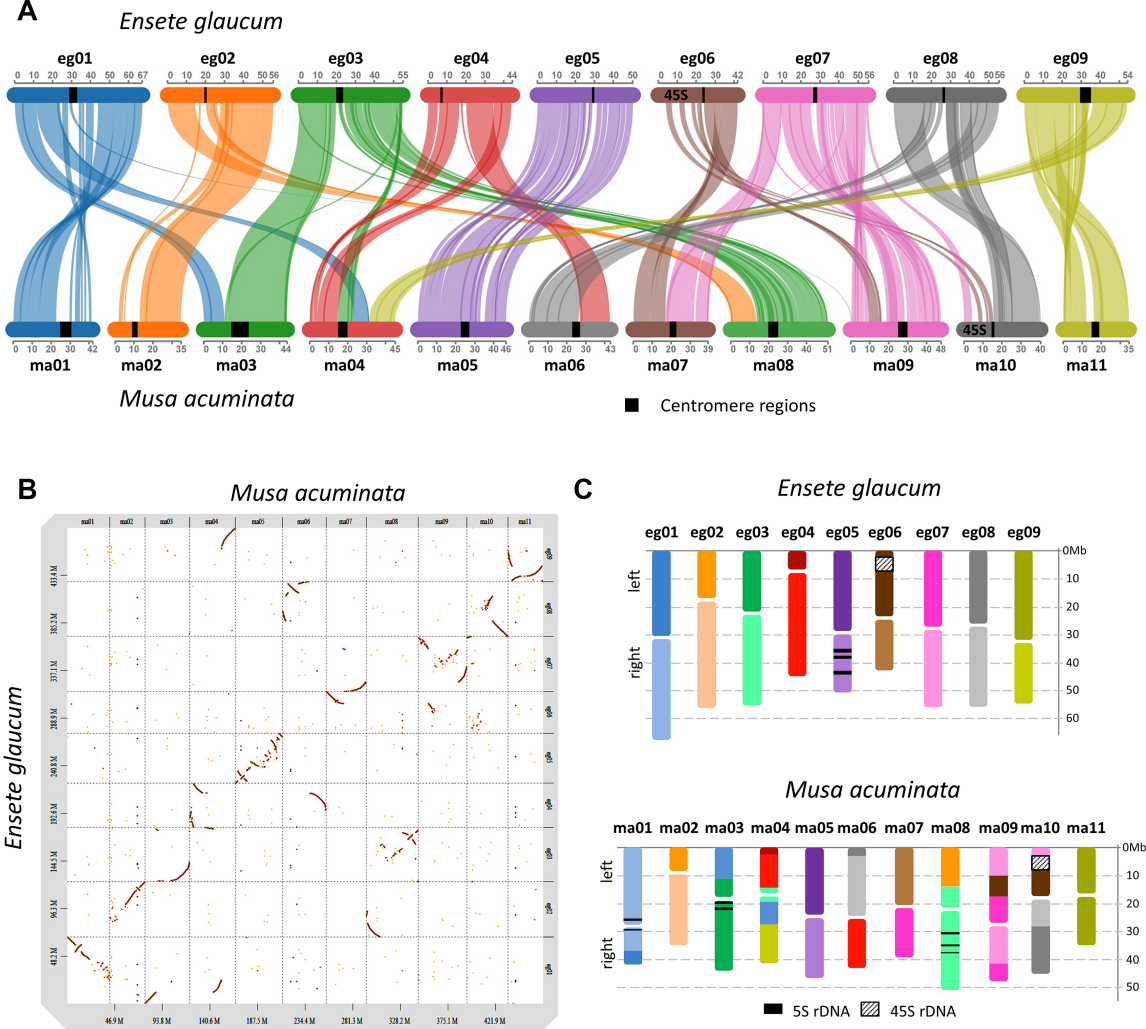
Synteny of *Ensete glaucum* and *Musa acuminata*. (A) Synteny plot (Synvisio) connecting syntenic genes in the 9 chromosomes of *E. glaucum* (egxx) and 11 chromosomes of *M. acuminata* (maxx). Syntenic blocks of high homology are indicated by uniformly coloured areas in the graphs. Only eg05 and ma05 maintain synteny over the full chromosome length, although there are some rearrangements. Three ma chromosomes are represented by part of 1 eg chromosome, while other ma chromosomes are fusions of >1 eg chromosome. (B) Dot plot comparing DNA sequences of *E. glaucum* and *M. acuminata* (for more detailed dot plots see Supplementary Fig. S9). (C) Representation of the syntenic blocks in the karyotypes of *E. glaucum* and *M. acuminata*. Chromosome rearrangements are shown, complementing the Synteny plot, while inversions and relative expansions and contractions of genome regions are clear.

The repeat analysis revealed the presence of an abundant tandemly repeated sequence with a monomer length of ∼134 bp (Fig. 5A). The sequence, named Egcen (*Ensete glaucum* centromere), represents ∼1.3% of the *E. glaucum* genome (45,000 copies) (Table 2, Supplementary Table S12; GenBank: OL310717) and forms arrays that are at places interspersed by the long interspersed nucleotide element (LINE) *Nanica* (described in *M. acuminata* [14]) and other sequences (Fig. 5B, see below). There were 1 or 2 major arrays of Egcen repeats found in the assemblies of all 9 chromosomes (Fig. 5C). *In situ* hybridization of Egcen showed that it was located around the primary, centromeric, constrictions as seen by DAPI (4´,6-diamidino-2-phenylindole) staining (Fig. 5D, see also Fig. 7A and C). The hybridization pattern of the FISH signal on all chromosomes showed variable strength and several sites grouped closely together, corresponding to the pattern seen in the assembly. The location of the Egcen arrays was therefore used to infer the centromere mid-point position in the *E. glaucum* chromosome assemblies (Fig. 2 outer circle, Fig. 8A, Supplementary Table S13).

Egcen was also detected at the centromeres of all *E. ventricosum* and *Musella lasiocarpa* chromosomes (Fig. 5E and F), showing similar distribution patterns with stronger and weaker signals as in *E. glaucum* (Fig. 5D), but it was not seen on *Musa* chromosomes by *in situ* hybridization (example of *M. balbisiana*, Supplementary Fig. S6) nor found in analysis of assemblies of *M. acuminata, M. balbisiana*, or *M. schizocarpa* (Supplementary Fig. S7A). The comparative RepeatExplorer clustering shows multiple satellite sequences found only in the *Ensete* and *Musella* genomes that are not present in the 3 *Musa* species tested (Fig. 4A) and supports Egcen being part of the tandem repeat birth and amplification that has occurred in *Ensete* and *Musella* after the split from *Musa*, and contrasts with the younger insertion times found for *Musa* retroelements (Supplementary Table S12 and Fig. 4C).

Tandem repeats or satellite DNA sequences are found around the centromeres of many plant (and animal) species [41, 42] and may be “centromeric” or “pericentromeric.” No equivalent tandem repeats were found in *Musa* [14, 17, 43], and the *E. glaucum* Egcen is not present either in *Musa* (Supplementary Figs S6 and S7A). However, the centromeric regions of all *M. acuminata* chromosomes have been shown to include multiple copies of a LINE non-LTR retroelement, *Nanica*, both by *in situ* hybridization and bioinformatic analysis [14, 17]. *Nanica*-related sequences were also identified in the *E. glaucum* assembly but with less abundance than in *Musa* (Supplementary Fig. S7B); ∼350 copies were mapped to chromosomes, mostly (but not exclusively) present interspersed within, or adjacent to, Egcen arrays (Fig. 5B and C and Supplementary Fig. S7B).

Assembly across centromeric regions including abundant repeats is difficult, and normally the tandem repeat elements are collapsed. The ONT long-molecule sequences allowed detailed examination of parts of the centromere region of chromosomes. A dot plot of an ONT read (coded 9c7e99b5, 96,300 bp long) aligned to the assembly of eg04 shows the complex organization of the Egcen array (Fig. 5B): this 100-kb region includes a total of 6 Egcen tandem blocks with between 3 and 126 repeats (a total of 385), 5 copies of *Nanica* (some rearranged, pink boxes), and 3 diverse retroelements flanked by LTRs (green boxes). Further copies of the Egcen tandem repeat occur in larger blocks over the following 450,000 bp of the assembly, and no genes were identified in the region.

A characteristic 17-bp long sequence, the canonical CENP-B box, is found within a monomer of a tandem repeat at centromeres of many species including human [44] and has been postulated to be necessary for binding of the centromeric CENP-B proteins regulating formation of centromere-specific chromatin. Within the Egcen sequence, there was a CENP-B related motif:

**Figure:**
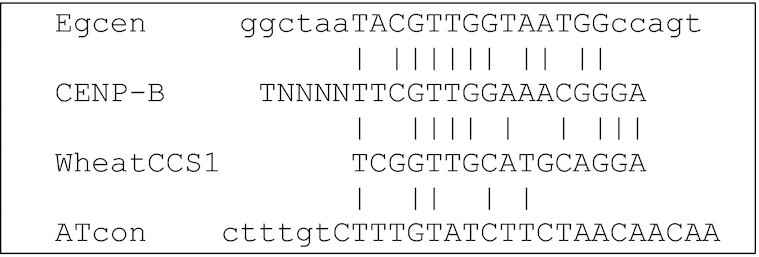


Although similar CENP-B motifs have been found from wheat and *Brachypodium* (CCS1 [45]) to *Arabidopsis* (ATcon [46]; see also review [47]), the relevance of the CENP-B–related box to centromere function remains uncertain, particularly when no similar tandem repeat is present in other species or the related genera such as *Musa* (see above). As is the case in many other species, the relative roles of retroelements, tandem repeats, and interspersed centromeric sequences, leading to recruitment of the centromeric proteins, are uncertain: the exact sequence or sequences that mark the functional centromeres remain enigmatic. The identification of a centromeric tandem repeat and the assembly across all centromere regions in *E. glaucum* together with data from *Musa* will allow protein-binding studies (with chromatin immunoprecipitation [ChIP] analysis) to resolve the functional centromere.

#### Microsatellites (simple sequence repeats)

Microsatellites were searched using Phobos and a simple sequence repeat (SSR) mining pipeline ([48]; perfect SSRs from mono to hexa-nucleotide repeats with 11 to 3 repeat numbers, respectively). SSR abundances, array lengths, and nucleotide base composition (70% were AT-rich) are shown in Fig. 6A and Supplementary Table S14. The overall nature and abundances of microsatellites in *E. glaucum* were generally similar to *M. acuminata, M. balbisiana, M. itinerans*, and *E. ventricosum* (Supplementary Table S14) and reflect that AT-rich microsatellites and dinucleotides (in particular AG/CT) are more frequent in Musaceae, in contrast to GC-rich satellites and trinucleotides being found more often in Poaceae genomes [49].

An average of 1 SSR was found per 4,000 bp, with the density lowest around the centromere and higher at the telomeres (Fig. 6B); they are excluded from the 45S NOR chromosome arm of eg06, and their overall distribution is similar to the distribution of protein-coding genes but contrasts with the more proximal distribution of LTR retroelements and DNA transposons (Fig. 2). Individual microsatellite motifs, however, showed characteristic and different distributions. The abundant microsatellites, CT and AAG, were synthesized as labelled oligonucleotides probes and used as probes for FISH on chromosomes. Both FISH to chromosomes (Fig. 6B and C) and the bioinformatic analysis of the assembly (Fig. 6D) showed that (AAG/CTT) has a relatively uniform distribution along chromosomes, while (AG/CT) shows depletion in centromeric regions and greater abundance in distal parts of chromosomes that are gene-rich (Fig. 2). The constraints on microsatellite spread in the genome are motif-specific, and, if SSR markers were to be used for genetic mapping, those associated with genes (such as AG/CT) would potentially be more useful.

#### 5S and 45S rDNA and rRNA genes

Tandem repeats of the rDNA were predominantly located within extended, complex loci on chromosomes eg05 (5S rDNA) and eg06 (45S rDNA) (Figs 2, 5C, 7, and 8). The 45S rDNA monomer containing the 18S rRNA gene - ITS1 - 5.8S rRNA gene - ITS2 - 26S rRNA gene - NTS (GenBank: OL310719) was 9,984 bp long, typical but slightly longer than other plant species [50–52]. The NTS region includes in most cases 16 copies of a degenerate 180-bp tandem repeat. On the basis of occurrence in the unassembled Illumina reads, there were 587 copies of the 45S rDNA monomer (1.21% of the genome, Table 2 and Supplementary Table S12). Although in the whole genome assembly the rDNA array was collapsed, the strength of the *in situ* hybridization signal using the rDNA sequence from wheat (Fig. 7A and B) is consistent with representing 1% of the genome. The long chromosome arm carrying the 45S NOR locus was depleted in protein-coding genes by 10-fold (mean of 12.6 genes/Mb compared with 127.6/Mb on the short arm; Fig. 7D). The single site of 45S rDNA at the NOR per chromosome set is similar to *M. acuminata* and other *Musa* species [53], although not *Ensete gilletii* (2n = 18), where there are 4 pairs of sites [23].

The 5S rDNA (GenBank: OL310718) comprised the 5S rRNA gene (119 bp long, typical for all plants; e.g., [51]) and intergenic spacer (937 bp), representing 0.078% of the genome or ∼366 copies, with a complete motif length of 1,056 bp (Supplementary Table S12). The 5S rDNA locus lies in the middle of the short arm of chromosome eg05, in 3 parts ∼34.5M, ∼37.5M, and ∼45.5M with multiple interruptions. An example of insertion of a 4.7-kb LTR retrotransposon-related sequence in the 5S rDNA array of tandem repeats is shown in Fig. 7E. In other regions of the *E. glaucum* ONT reads or assembly, the retroelement-related sequence named *Brep*, reported in *Musa* [54], was also found in the 5S rDNA arrays. Garcia et al. [55] show the rather unusual and highly complex structure of 5S rDNA in *M. acuminata* using graph-based clusters of reads with multiple IGS and retroelement components, supporting the complexity reported here in the *E. glaucum* assembly. The multiple hybridization sites evident from the *in situ* hybridization site on 1 pair of chromosomes (Fig. 7B and C), with several, non-continuous, signals visible in the extended prometaphase chromosomes (Fig. 7C, insets), support the non-continuous nature of the 5S rDNA array.

The 5S rDNA monomer length of 1,056 bp was exceptionally long in comparison to any other plant species (typically 400–500 bp long). The first 400 bp of the intergenic spacer had no significant BLAST hits in GenBank, while the second part showed only short regions with weak homology largely to chromosome assemblies of *Musa* species in GenBank. There were no motifs characteristic of retroelements in the 937-bp intergenic spacer. It is unclear why the monomer length for the 5S rDNA in *E. glaucum* should be twice that typical in other species, including *Musa*, and was to be relatively homogeneous over all copies (Fig. 7E). Furthermore, in contrast to the single locus on eg04 of *E. glaucum*, all species of *Musa* examined so far have multiple 5S rDNA sites (2, 3, or 4 per genome), and *E. gilletii* had 3 pairs of sites [23, 53].

Different species in the Triticeae show wide variation in numbers and locations of both 45S and 5S rDNA sites, suggesting multiple and complex evolutionary rearrangements of the chromosome arms [56] even in the absence of chromosomal rearrangements including translocations and inversions. Dubcovsky and Dvorák [57] have considered the 45S rDNA loci as the “nomads of the Triticeae genomes” given their repeated evolutionary changes in position during species radiation without rearrangements of the genes of the linkage groups. The depletion of protein-coding genes in chromosomal regions extending over most of a chromosome arm around the 45S rDNA genes is notable in *Musa* and *Ensete*, so chromosome rearrangements can lead to loci moving, although other recombination, duplication, deletion, or translocation events must occur to alter the numbers of both 5S and 45S loci observed.

### Synteny and chromosome rearrangements to *Musa*

Structural comparisons of the *E. glaucum* genome assembly (x = 9, chromosomes eg01–eg09) were performed with the high-quality assembled genomes of *M. acuminata* (x = 11, ma01–ma11; v4 [17, 58]) based on synteny (Fig. 8A). The comparison was also extended to *M. balbisiana* (mb01–mb11 [15]; Supplementary Fig. S8). Sequence dot plots (Fig. 8B) and comparative karyotypes (Fig. 8C and Supplementary Table S15) of the *E. glaucum* genome against the *M. acuminata* genome were also analysed.

Overall, the genome assemblies of *M. acuminata* and *E. glaucum* are similar in length and gene content (Supplementary Table S5). We observed high identity between segments of the 9 chromosomes of *E. glaucum* and of the 11 chromosomes of the *Musa* (Fig. 8A, Supplementary Fig. S8). Broad centromeric regions with few protein-coding genes (Fig. 2) cannot show syntenic domains. The number of collinear genes was 48,956 between *E. glaucum* and *M. acuminata*, and 39,604 between *E. glaucum* and *M. balbisiana* (by comparison, the A and B genome of *Musa* show 42,854 collinear genes). Chromosomes show rearrangements, inversions, expansions, or contractions by crossed, converging, or spreading lines in the Synvisio plots (Fig. 8A). The dot plot (Fig. 8B; single chromosome comparison in Supplementary Fig. S9) shows that there are some syntenic regions distributed over the same length of chromosomes in both species (diagonal lines showing synteny at 45°, eg08/ma10). In other cases, there is expansion in 1 genome and not in the other (lines of synteny more vertical, eg04/ma04, or nearer horizontal, eg03/ma04). Many syntenic segments showed curved lines (ma03/eg03), showing relative expansion of 1 genome at 1 end of the conserved syntenic block, and expansion of the other genome at the other end.

One complete chromosome, ma05/eg05/mb05, was similar with the same gene content in all 3 species (Fig. 8A and Supplementary Fig. S8), but it showed multiple internal inversions and expansions/contractions. A nested pair of inversions was evident covering 10.4 Mb near the start of the chromosome in *E. glaucum* with respect to *M. acuminata* (8.84-Mb region) in dotp lots (Supplementary Fig. S9A) and by comparison of locations of orthologous genes (Supplementary Fig. S10). In the context of the *E. glaucum* inversions, we could also examine the ancestral structure of *M. acuminata* and *M. balbisiana* reported by Wang et al. [15]. Notably, a major rearrangement involving an inversion between *M. acuminata* ma05 and *M. balbisiana* mb05 [15] was the same inverted region as found in eg05, with an additional nested inversion of 3.1 Mb in eg05 with respect to ma05 (Supplementary Figs S8–S10). Using positions of orthologous genes at the boundaries of syntenic regions, the inversion structure between chromosomes eg05, ma05, and mb05 was clear. Regardless of the ancestral condition, the result indicates that closely similar inversion breakpoints were involved (at the ends of the segment, Supplementary Fig. S10) twice during evolution.

Apart from chromosome 5, an additional 3 whole chromosomes of *M. acuminata* are represented largely by a single, whole-chromosome region/arm of *E. glaucum*, with some rearrangements occurring within the chromosomes (Fig. 8): ma01 is mainly the right arm of eg01; and ma02 is mainly the right arm of eg02 (Fig. 8A); ma11 is entirely the left arm of eg09 (see details in the dot plot of Supplementary Fig. S9B). The other arms of these 3 *E. glaucum* chromosomes (eg01, eg02, and eg09) and the remaining 6 chromosomes are related to blocks of the remaining 7 *Musa* chromosomes. Four *Musa* chromosomes have translocated fusions of segments of 2 *Ensete* chromosomes. ma09 has the intercalary region of eg07, with an intercalary segment of eg06 inserted within the eg07 region. ma10 includes parts of 3 *Ensete* chromosomes, while ma04 has 4 segments from *Ensete* chromosomes (Fig. 8A and C). The 45S rDNA on eg06 and ma10 are surrounded by syntenic regions but are both depleted in protein-coding genes (Fig. 2). In contrast, the 5S rDNA sites are not surrounded by other orthologous genes (see above). The non-reciprocal translocation noted by Wang et al. [15] of a terminal segment between ma03 and mb01 lies within a larger syntenic block shared between ma03 and eg03. This suggests that the translocation occurred in the *M. balbisiana* lineage (Supplementary Fig. S8).

In several chromosomes of both *E. glaucum* and *M. acuminata*, the breakpoints occur in the centromeric regions (e.g., in eg02, eg03, eg07, eg08, and eg09; ma01, ma06, and ma10; Fig. 8A). While some breakpoints occur at or adjacent to centromeres, the exact relationship of any breakpoint to the centromere and Egcen or *Nanica* sequences is diverse. Notably, eg03 is spanning the centromere of 3 *Musa* chromosomes, ma03, ma04, and ma08, and in other cases centromere regions are different despite surrounding synteny (e.g., eg07, ma07, and ma09). Telomeric or subtelomeric regions are conserved between the 2 species in 7 of the 18 *E. glaucum* chromosome arms (e.g., eg01/ma01, eg06/ma07, eg08/ma10; the dot plot homology lines end in the corners of the chromosomes, Fig. 8B and Supplementary Fig. S9B). In other chromosomes, telomeres in *E. glaucum* are in intercalary regions of *Musa* (see eg03/ma03 with inversion, eg08/ma06 in Fig. 8B; and in detail eg08 and ma10 in Supplementary Fig. 9C). The homology of the whole chromosome ma11 and the left arm of eg09 (see above) indicates a fusion/fission event with loss/gain of centromere and telomere function, but they are also predicted for the other rearrangements discussed above.

Song et al. [10] review the data on the basic chromosome number of the Zingiberales, concluding that x = 11 is most reasonable original basic number, with x = 9 as a derived basic number. With chromosome numbers of x = 9, 10, and 11 predominant in Musaceae, this family is particularly suitable to explore the nature and locations of chromosomal fusions and fissions that are predicted to often occur in similar position during karyotype evolution (e.g., in wheat [59]). The availability of high-quality genome assemblies, based on the ONT, Hi-C, and in *Musa* BioNano and Pacific Biosciences technologies, will allow the nature of breakpoints in chromosome fission events to be investigated at the sequence level between the *Musa* x = 11 and *Ensete* x = 9 species, as well as being able to shed light on centromere and telomere function.

## Conclusions

We provide a chromosome-scale assembly of *Ensete glaucum*, a sister genus to *Musa*. This assembly is valuable to infer *Musa* genome evolution, enabling comparison with putative last common ancestors of *M. acuminata* (A genome) and *M. balbisiana* (B genome) at protein and chromosomal levels. Most striking was the multiple rearrangements of chromosome structures between *E. glaucum* and the *Musa* A and B genomes, with only 4 of the 11 *M. acuminata* chromosomes (and only 3 of the 11 in *M. balbisiana*) showing synteny with only 1 or part of 1 *E. glaucum* chromosome. With the new insight into chromosome evolution here, further assemblies (in particular the *Callimusa* section with n = 7, 9, and 10) will enable resolution of ascending or descending dysploidy, in Musaceae, its sister clades in the Zingiberales, and more widely.

As well as the complex chromosome rearrangements, repetitive sequences differ extensively between the *Musa* and *Ensete* genera. There is a major tandem repeat at the centromeres of only the *Ensete* species, showing lack of conservation of this key structural element of chromosomes, although both genera have multiple copies of the *Nanica* retroelement in centromeric regions. *E. glaucum* has only 1 5S rDNA locus, with an unusually long monomer of 1,056 bp.

The complete sequence provides an accurate reference for the genus for gene identification, marker development, genotyping-by-sequencing, and genome-wide association studies and will accelerate our understanding of the molecular bases of traits such as cold tolerance and starch accumulation and allow identification of relevant genes, contributing to the aim of the Earth BioGenome Project [60] to sequence all eukaryotic species. Although not yet fully understood, the role of chromosomal structural variation and sequence copy number variation (both of genes and repetitive DNA) in genotypic and species diversity is increasingly being recognized, and our high-continuity assembly provides a reference for such studies. The work builds towards a complete pangenome of the Musaceae family, defining structural, gene, and genetic diversity, which can be used for genetic improvement across the Musaceae and more widely.

## Material, Methods, and Validation

### Sample collection and distribution

The individual *Ensete glaucum* plant used for genome sequencing and analysis was collected from Puer city, Yunnan province, China and maintained in the South China Botanical Garden, Guangdong province, China (accession No. 19990288; Fig. 1A–E). The distributions of *Ensete* and *Musa* species were identified in databases of Flora of China, South China Botanical Garden, iNaturalist, GBIF [61] (excluding cultivation sites), and regional distribution maps. Figure 1F was then made from POWO [62] (overlaid and colour-adjusted in Adobe Photoshop CC2018).

### DNA extraction and sequencing

Young leaves of *Ensete glaucum* were collected and ground into powder in liquid nitrogen. High molecular weight genomic DNA was extracted using the DNeasy Plant Mini Kit (Qiagen, Hilden, Germany). DNA quality was assessed by agarose gel electrophoresis and NanoDrop 2000c spectrophotometry, followed by Thermo Fisher Scientific Qubit fluorometry.

#### Illumina sequencing

A genomic DNA library with 400-bp fragments was constructed using Truseq Nano DNA HT Sample preparation Kit (Illumina, USA), and 150-bp paired ends were sequenced with Illumina Novaseq (Illumina NovaSeq 6000 Sequencing System, RRID:SCR_020150) by Grandomics Biosciences Co., Ltd. (Wuhan, Hubei, China) (previously known as Nextomics, Wuhan, Hubei, China). After applying Trimmomatic v0.36 (Trimmomatic, RRID:SCR_011848) [63] to trim adaptors, filtering out low-quality reads and further quality control with fastQC v0.11.9 (FastQC, RRID:SCR_014583) [64], 246 million paired reads and 36.88 Gb of data resulted (Table 1).

#### Oxford Nanopore sequencing

ONT (Oxford Nanopore Technologies, Oxford, the UK) sequencing was performed by Grandomics Biosciences Co., Ltd. (Wuhan, Hubei, China): long fragments longer than 12 kb were selected with Sage Sciences BluePippin (Sage Science BluePippin system, RRID:SCR_020505), and the SQK-LSK109 kit (Oxford Nanopore, Oxford, the UK) was used to build a library that was sequenced using PromethION (PromethION, RRID:SCR_017987), flow cell R9.4.1. The base calling was performed with Guppy v2.0.8 and reads mean_q score_template (Phred) > 7 (base call accuracy >80%) were selected. A total of 129 Gb ONT reads (∼250× coverage) was generated. fastp v0.19.7 (fastp, RRID:SCR_016962) [65] was used for quality control including adaptor-trimming, filtering reads with too many Ns or mean q score <7, and resulted in remaining clean data of 109 Gb (Table 1). The mean read length was ∼20 kb, with the longest >120 kb (Supplementary Fig. S11).

#### Hi-C chromatin interaction data

Genomic DNA was extracted from *E. glaucum* for Hi-C analysis and generation of a contact map to anchor contigs onto chromosomes [66, 67]. First, freshly harvested leaves were cut into 2-cm pieces and vacuum infiltrated in nuclei isolation buffer supplemented with 2% formaldehyde. Crosslinking was stopped by adding glycine and additional vacuum infiltration. Fixed tissue was then ground to a powder before resuspending in nuclei isolation buffer to obtain a suspension of nuclei. The purified nuclei were digested with 100 units of DpnII and tagged with biotin-14-dCTP. Biotin-14-dCTP from non-ligated DNA ends was removed owing to the exonuclease activity of T4 DNA polymerase. The ligated DNA was sheared into 300−600 bp fragments and then was blunt-end repaired and A-tailed, followed by purification through biotin-streptavidin-mediated pull down. Finally, the Hi-C libraries were quantified and sequenced using the Illumina HiSeq platform (performed by Grandomics). Low-quality sequences (quality scores <20), adaptor sequences, and sequences shorter than 30 bp were filtered out using fastp v0.19.7 [65].

### Genome and chromosome assembly

ONT data were corrected by Nextdenovo v2.0-beta.1 [68], with setting “read_cutoff = 3k, seed_cutoff = 25k, blocksize = 2g” and the 109-Gb filtered data were assembled by SMARTdenovo (SMARTdenovo, RRID:SCR_017622) [69] with the parameters “wtpre -J 3000, wtzmo -k 21 -z 10 -Z 16 -U -1 -m 0.1 -A 1000, wtclp -d 3 -k 300 -m 0.1 -FT, wtlay -w 300 -s 200 -m 0.1 -r 0.95 -c 1”. To polish the assembly, contigs were refined with Racon (Racon, RRID:SCR_017642) [70], BWA v0.7.17 (BWA, RRID:SCR_010910) [71] was used to map the filtered Oxford Nanopore reads to the assembly, and NextPolish v1.3.1 [72] with parameters “ –consensus -w window -t 4 -m 0.5 -d 30” was used to discard possibly redundant contigs and generate a final assembly; similarity searches were performed with the parameters “identity 0.8 – overlap 0.8”. Finally, BWA v0.7.17 [71] and Pilon v1.21 (Pilon, RRID:SCR_014731) [73] with setting “–changes –vcf –diploid –fix bases –threads 10 –mindepth 10” were used to further correct the assembly using the Illumina Novaseq reads, and 2 rounds of mapping back to the assembly each time with further correction were undertaken. A 494 Mb assembly with 124 contigs was achieved (Table   2 and Supplementary Tables S1 and S2).

Read pairs from the Hi-C data were mapped to the draft assembly using bowtie2 v2.3.2 (bowtie2; RRID:SCR_016368) [74] with the settings “-end-to-end, -very-sensitive and -L 30” to select unique mapped paired-end reads. Valid interaction paired-end reads were identified by HiC-Pro v2.8.1 (HiC-Pro, RRID:SCR_017643) [75] and retained for further analysis while invalid read pairs, including dangling-end, self-cycle, re-ligation, and dumped products were discarded. The scaffolds were further clustered, ordered, and oriented onto pseudo-chromosomes by LACHESIS (LACHESIS, RRID:SCR_017644) [76], with parameters as follows: “CLUSTER_MIN_RE_SITES = 100, CLUSTER_MAX_LINK_DENSITY = 2.5, CLUSTER NONINFORMATIVE RATIO = 1.4, ORDER MIN N RES IN TRUNK = 60, ORDER MIN N RES IN SHREDS = 60”. Finally, regions with obvious discrete chromatin interaction were detected and their placements and orientations were manually adjusted (Supplementary Fig. S12). Validation of the assembly was performed using BUSCO v5 (BUSCO, RRID:SCR_015008) [21] to assess the completeness and presence of 1,614 genes in the embryophyta_odb10 database in “genome” mode (Supplementary Table S3a).

### RNA extraction, sequencing, and transcriptome assembly

Total RNA was extracted from fresh leaves and roots of the same individual of *E. glaucum* that was used for genomic sequencing using RNeasy Plant Mini Kit (Qiagen, Shanghai, China). Illumina libraries were built from 1 µg total RNA of each sample with TruSeq RNA Library Preparation Kit (Illumina, USA) and were then sequenced using Illumina Novaseq platform to generate paired-end reads. A transcriptome assembly was produced using RNA-seq data using Trinity (Trinity, RRID:SCR_013048) [77] with parameters: “–genome_guided_bam EGL.star.bam –max_memory 50G –genome_guided_max_intron 10000” and mapped on the genome with PASA (PASA, RRID:SCR_014656) [78] with setting: “–MIN_PERCENT_ALIGNED = 80 –MIN_AVG_PER_ID = 80”.

#### Genome size estimation

Using the Illumina DNA sequence, genome size was estimated from the 17-mer frequency using Jellyfish v2.0 (Jellyfish, RRID:SCR_005491) [79] with the formula *k*-num/*k*-depth (where *k*-num is the total number of 17-mers, 30,417,960,841; and *k*-depth the highest *k*-mer depth, 54; Table 2). Then 21-mer data were used in findGSE [80] and Genomescope 2.0 (Genomescope R, RID:SCR_017014) [81] to estimate the genome size and heterozygosity (Supplementary Fig. S1).

MGSE v0.4 [22] were also used to estimate the genomic size based on read-mapping coverage. The next-generation sequencing reads were mapped to assembly by BWA v0.7.17. The coverage of single-copy genes (BUSCO genes) was calculated by MGSE.

### Gene annotation

We adopted a combination of *ab initio* gene prediction, homology-based gene prediction, and transcriptome-based gene prediction strategy. RepeatMasker v4.0.9 (RepeatMasker, RRID:SCR_012954) with option “-no_is –xsmall” was used to generate a repeat softmasked genome file. RNA-seq data from leaf and root tissues were mapped to the masked genome assembly with STAR v2.7 (STAR, RRID:SCR_004463) [82] with option: “–outSAMtype BAM SortedByCoordinate –outSAMstrandField intronMotif –outFilterIntronMotifs RemoveNoncanonical”. The RNA alignment was input into BRAKER2 v2.1.5 (BRAKER, RRID:SCR_018964) [83], a combination of GeneMark (GENEMARK, RRID:SCR_011930) [84] and AUGUSTUS (RRID:SCR_008417) [85], to perform *ab initio* gene predictions with the default settings. The gene models from BRAKER2 were input into MAKER v2.31.10 (MAKER, RRID:SCR_005309) [86] as model, and the RNA alignment of *E. glaucum* and proteins from *M. acuminata* v2 were used as Expressed Sequence Tag (EST) and protein evidence, respectively. We also used GeMoMa v2.3 (GeMoMa, RRID:SCR_017646) [87] to perform homology-based gene prediction using *M. acuminata* v2 [16] as reference annotated genome. EvidenceModeler (EvidenceModeler, RRID:SCR_014659) [88] was used to combine *de novo* and homology-based predictions and our transcriptome evidence to produce the final structural gene annotation.

To annotate the function of predicted genes, we performed BLASTP (e-value = 1e−10) (BLASTP, RRID:SCR_001010) from the BLAST+ package [89] for each predicted coding sequence against the databases: UniProtKB/Swiss-Prot, UniProtKB/TrEMBL [90], and NR (non-redundant protein database at NCBI). These sequences are then processed to produce a non-identical (often referred to as pseudo non-redundant) prediction. To assign a putative function to a polypeptide we kept only the best hit on the basis of 3 parameters: (i) Qcov (Query coverage = length high-scoring segment pair [HSP]/length query), (ii) Scov (Subject coverage = length HSP/length subject), and (iii) identity. Additional functional information was added by scanning sequences with InterProScan v5.46 (InterProScan, RRID:SCR_005829) [91]. Blast2GO v6.0.1 (Blast2GO, RRID:SCR_005828) [92] was used to integrate the results of BLAST and InterProScan, and to link the GO (Gene Ontology) terms to genes accordingly (Supplementary Table S4). The functional annotation procedure is given in greater detail at [93].

BUSCO was run in mode “transcriptome” using the embryophyta_odb10 database to assess the gene annotation results and found 1,529 (94.7%) complete BUSCOs (Supplementary Table S3b).

### Gene family analyses

#### Orthogroups identification in Musaceae

Protein-coding genes from *M. acuminata* [16], *M. balbisiana* v1.1 [15], and *M. schizocarpa*v1 [13] were retrieved from the Banana Genome Hub [18]. Protein-coding genes predicted from *E. ventricosum* were downloaded at NCBI Genome (GCA_000818735.3) to allow discrimination of *Ensete*-specific orthogroups and *E. glaucum*–specific orthogroups. Combined with *E. glaucum* protein-coding genes, we used OrthoFinder v2.5.2 (RRID:SCR_017118) [94] and Diamond [95] with default parameters (summary in Supplementary Fig. S5). Visualization (Fig. 3B) was produced with UpsetR [96]. Gene ontology (GO) enrichments were calculated using TopGO [97] with Fisher exact test (Supplementary Fig. S2 and Supplementary Table S8).

#### Gene family expansion and contraction

To identify gene family expansion and contraction, we expanded previous analyses with OrthoFinder by adding a representative of Musaceae sister clades in Palms (*P. dactylifera*, date palm [98]) and Poales (*O. sativa* v7, rice [99]; data downloaded from Phytozome [100]) but omitting *E. ventricosum* owing to gene redundancy. The longest transcripts were kept if alternative splicing occurred. Divergence time estimation with approximate likelihood calculation used MCMCTREE in PAML v4.9j (PAML, RRID:SCR_014932). CAFE (CAFE v4.2.1, RRID:SCR_018924) [101] was used to model the evolution of gene family sizes and stochastic birth and death processes and summarized in the phylogenetic tree (Fig. 3C).

#### Transcription factors

Protein-coding gene sequences for *E. glaucum* and *M. acuminata* v2 were searched in PlantTFDB v5.0 (PLANTTFDB, RRID:SCR_003362) and iTAK online v1.6 [102]. Predicted TFs were verified through a hidden Markov model (HMM) with PFAM searching tools using the cut-off E-value of 0.01 (Fig. 3D and Supplementary Table S9). Genes were verified by PFAM (Pfam, RRID:SCR_004726), CDD (Conserved Domain Database, RRID:SCR_002077), and SMART (SMART, RRID:SCR_005026) databases.

### Whole-genome duplication

To identify the WGD events, we applied WGDI pipeline (whole-genome duplication identification v0.4.7 [103]). The predicted proteins of *E. glaucum* were blasted against themselves and then a collinearity analysis was conducted. The Ks (the synonymous rates of substitution) between genes in paired collinearity gene groups were calculated and the Ks peak was detected. For comparison, the same processes were also applied to *M. acuminata* v2 [16].

#### Synteny analyses

Structural comparisons of the *E. glaucum* genome were performed with *M. acuminata* v4 (designated the A genome) and *M. balbisiana* (B genome). The recent release of *M. acuminata* v4 assembly was preferred in this case because it improved pericentromeric regions and provided telomere-to-telomere gapless chromosomes [17]. Assemblies were aligned with minimap2 (Minimap2, RRID:SCR_018550) [104] and visualized results using D-Genies (D-GENIES, RRID:SCR_018967) v1.2.0 [105]. Protein-coding genes were processed to identify reciprocal best hits with BLASTP (e-value 1e−10) followed by MCScanX (e-value 1e−05, max gaps 25) [106] and results imported in SynVisio [58] for syntenic block visualization. Scale bars and colouring of the chromosome bars was adjusted using Adobe Photoshop CC2018.

The karyotype of *E. glaucum* in Fig. 8C was prepared from lengths of each pseudo-chromosome (Supplementary Table S2) with the estimated centromere position (using the Egcen array midpoints, Supplementary Table S13) to estimate the left (darker coloured) and right (lighter coloured) chromosome arms. Chromosome lengths and centromere positions for *M. acuminata* were taken from [17] (Fig. 2a); syntenic blocks were calculated using the SynVisio diagram (Fig. 8A).

### Repetitive DNA identification and annotation

For repetitive DNA analysis, publicly available programs (see below and [38]) as well as manual searches and sequence comparisons were applied. Geneious v.10.2.6 (Geneious, RRID:SCR_010519) (Biomatters Ltd., Auckland, New Zealand) was used to produce the dotp lots of Figs 5A, B, 7E, and 8B, and Supplementary Fig. S9).

In the assembly, repeated sequences were first searched with REPET v2.5 pipeline [107]. The top 100 repeated sequences were plotted on the 4 reference *Musa* genome assemblies (i.e., *M. acuminata, M. balbisiana, M. schizocarpa*, and *E. glaucum*) using BlastAndDrawDensity.py script described in [17] and available on the GitHub repository [108].

#### Transposable elements

TEs were annotated by EDTA pipeline [109], which integrates various software to discover TEs including LTR retrotransposons [110–112], terminal inverted repeat (TIR) transposons [113], short TIR transposons or miniature inverted transposable elements (MITEs) [114], and Helitrons [115]. According to suggestions in [109], we also adopt RepeatModeler2 (RepeatModeler, RRID:SCR_015027) v2.0.1 [116] to find remaining TEs.

We also discovered repetitive elements through the REPET v2.2 [117] package including TEdenovo and TEannot. The TEdenovo procedure was used on masked assembly to produce a batch of 4,229 TE consensus sequences. From these 2,800 consensus sequences, only those with full-length fragments present in the assembly were kept for further analysis, quantification, and annotation with the TEannot procedure. A first annotation was performed using public Repbase (Repbase, RRID:SCR_021169) release 20.05, followed by *Gypsy/Copia* retroelement family identification using HMMs (hmmsearch version 3) to search consensus for corresponding retro-transposase PFAM domains PF04195 and PF14244, respectively. The above results were then combined and CD-HIT v4.1.8 (CD-HIT, RRID:SCR_007105) [118] was used to reduce redundancy. The LTR retrotransposons were sent to TEsorter [119] to classify into lineage level and RT domain amino acid sequences were extracted. Phylogenetic trees of *Copia* and *Gypsy* were inferred by RT domain alignment results (Supplementary Fig. S4). The proportions of TEs in the assembly are given in Table 2 (Supplementary Tables S11 and S12) and chromosomal distributions in Fig. 2.

To estimate ages of LTR retrotransposons and the time of insertion (Fig. 4B and C and Supplementary Fig. S5), complete elements were found by LTRharvest v1.6.1 (LTRharvest, RRID:SCR_018970) [110] and LTR_retriever (LTR_retriever, RRID:SCR_017623) [111] and then classified by TEsorter v1.3. The estimation of time was based on the divergence of the 5′- and 3′-end LTRs, and these 2 LTRs of every LTR retrotransposon were extracted into separate files with a custom script. The 5′ and 3′ LTRs were aligned by MUSCLE v3.8.1551 [120]. The divergence distances under K2P evolutionary model were calculated by R package ape v5.4-1 (ape, RRID:SCR_017343). The average base substitution rate was selected to be 11.3E−8 [121]. The insertion time *T* was calculated as *T* = *K*/(2*r*), with *r* as the rate of nucleotide substitution and *K* as the divergence distance between LTR pairs. The script to perform the analysis is on GitHub [122].

#### Graph-based clustering of reads using RepeatExplorer

A sample of 2 Gb of the Illumina HiSeq raw reads were used for assembly-free analysis by RepeatExplorer2 [39]. Graph-based clusters of similar sequence fragments were generated under default parameters. Clusters were assigned to repeat classes and retroelement lineages using the automated Repeat Masker and Domain hits provided by the program (Supplementary Table S11). Comparative analysis with sample Illumina sequence reads from 5 other Musaceae species, namely, *M. acuminata* v2 [16], *M. balbisiana* v1.1 [15], and *M. schizocarpa* v1 [13], *E. ventricosum* [20], and *M. lasiocarpa* (Wang, Cui, Rouard, Schwarzacher, Heslop-Harrison, Liu in preparation)) were also analysed (Supplementary Fig. S3) and compared with RepeatExplorer2 following “comparative repeat analysis” protocol. The results were visualized by R script “plot_comparative_clustering_summary.R” (Fig. 4A).

#### SSR tandem repeats

The genome assembly was searched for SSR (microsatellite) motifs using the SSR mining pipeline developed by Biswas et al. [48]. Searches were standardized for mining perfect SSRs from mono- to hexanucleotide repeats (minimum repeat number of 12 for mononucleotides, 8 for di-, 5 for tri-, tetra-, and penta-, and 4 repeats for penta- and hexanucleotides). SSR abundance and nature was analysed on the basis of density in the genome (∼1 per 4,000 bp), array length (Fig. 7B and Supplementary Table S14; SSR search parameters minimum lengths mono = 1*12 = 12nt, di = 8*2 = 16nt, tri = 3*5 = 15nt, tetra = 4*5 = 15nt, penta = 5*4 = 20nt and hexa = 6*4 = 24nt; total SSR count 123884; Class I>20nt and Class II≤20nt), nucleotide base composition of the SSR loci (70% were AT-rich), and abundance of each motif.

### Fluorescent *in situ* hybridization

Chromosome preparation and FISH were performed as described by Schwarzacher and Heslop-Harrison [123] with minor modifications. Plants of *E. glaucum, E. ventricosum, M. lasiocarpa* (purchased commercially), and *M. balbisiana* “Butuhan” (ITC1074) [124] were grown in the glasshouse at the University of Leicester, UK. Actively growing root tips were treated with 2 mM 8-hydroxyquinoline and fixed with 96% ethanol:glacial acetic acid (3:1). For chromosome preparations, roots were digested with a modified enzyme solution (32 U/mL cellulose, Sigma-Aldrich C1184; 20 U/mL “Onozuka” RS cellulose; 35 U/mL pectinase from *Aspergillus niger*, Sigma-Aldrich P4716; 20 U/mL Viscozyme, Sigma-Alderich V2010) in 10 mM citric acid/sodium citrate buffer (pH 4.6) for 3–5 h at 37°C and then kept in buffer for 12–30 h at 4°C. Meristems were dissected in 60% acetic acid and routinely 2–6 slide preparations were made from each root. Slides were stored at −20°C until FISH.

The 45S rDNA probe was labelled by random priming (Invitrogen) with digoxigenin dUTP or biotin dUTP (Roche) using the linearized clone pTa71 [125] containing the 45S rDNA repeat unit of *Triticum aestivum*. A 50–100 ng quantity of labelled probe was used per slide and detection of hybridization sites was carried out with fluorescein-conjugated anti-digoxigenin (Roche) or streptavidin conjugated to Alexa-647 (Molecular Probes, Invitrogen). The remaining probes were designed from the consensus sequence of the centromeric repeat Egcen (Fig. 5) and the 5S rDNA (Fig. 7) or as SSRs (Fig. 6); as directly labelled oligonucleotides (200–500 ng per slide) they needed no further detection and were as follows:

CenCy3: EGL_2640R: [Cyanine3]GAC CGT CGC ATT TTT TGG CGA AAC CAT GCT CGT ACG ACT TCC CAT GGG CTA AAA CGT TAG GA
CenFAM: EGL_G2640L: [6FAM]GGC CTA TAT TTT GAA ATT CCG AGA CGG TGC ATG AAA AAC CGA TCG AAA CGA AAC ATT GCG
5S_4M_Cy3: [Cyanine3]TCA GAA CTC CGA AGT TAA GCG TGC TTG GGC GAG AGT AGT AC
5S_3R_Cy3: [Cyanine3]AGT ACT AGG ATG GGT GAC CCC CTG GGA AGT CCT CGT GTT GC
5S_6L_Cy3: [Cyanine3]GCG ATC ATA CCA GCA CTA AAG CAC CGG ATC CCA TCA GAA CTC C
(AAG)15_FAM: [6FAM]AAG AAG AAG AAG AAG AAG AAG AAG AAG AAG AAG AAG AAG AAG AAG
(CT)23_TAMRA: [TAMRA]CTC TCT CTC TCT CTC TCT CTC TCT CTC TCT CTC TCT CTC TCT CTC T

For hybridization, probes were prepared in 40% (v/v) formamide, 20% (w/v) dextran sulphate, 2× SSC (sodium chloride sodium citrate), 0.03 μg of salmon sperm DNA, 0.12% SDS (sodium dodecyl sulfate), and 0.12 mM EDTA (ethylenediamine-tetra acetic acid). Chromosomes and 40–50 µL of probe mixture were denatured together at 72°C for 8 mins, cooled down slowly, and allowed to hybridize overnight at 37°C. Post-hybridization washes were at 42°C in 0.1× SSC, giving a stringency of 80–85% for the short oligo probes and 70–75% for the 45S rDNA probe. Chromosomes were counterstained with 4 µg/mL DAPI and mounted in CitifluorAF. Slides were examined using Nikon Eclipse 80i microscope and images were captured with a DS-QiMc monochrome camera, and NIS-Elements v2.34 (Nikon, Tokyo, Japan) assigning colour and merging channels. Overlays of hybridization signal (shown in green or red) and DAPI images (in cyan or blue) were enhanced with Adobe Photoshop CC2018 using only cropping and functions that treat all pixels of the image. Seven FISH runs with different combinations of probes and replicates were performed, and between 5 and 15 metaphases per slide (99 metaphases in total from 15 slides) were analysed in detail.

## Data Availability

All supporting data can be found in the *GigaScience* database [127].

Raw sequence reads (RNA-seq, Illumina HiSeq, ONT, and Hi-C) were deposited in NCBI under accession No. PRJNA736572. Specifically, ONT raw reads: SRX11350424 and SRX11350425; RNA-seq raw reads, as follows: leaf: SRX11350426; root: SRX11350427; genomic Illumina short-read data: SRX11350423; raw reads of the Hi-C library: SRX11350428 and SRX11350429. The raw read data were also deposited in the Genome Sequence Archive (GSA) of the China National Center for Bioinformation (accession code: CRA004283).

The assembled genome was also deposited to GenBank in NCBI and can be accessed via accession No. JAHSUZ000000000. Genome assembly, gene and TE annotation data, and transcriptomic data are also available on the Banana Genome Hub (http://banana-genome-hub.southgreen.fr/) for download or exploration via a dedicated Genome Browser (Jbrowse) and syntenic browser (SynVisio).

## Additional Files

**Supplementary Figure S1**. Genomescope analysis of heterozygosity.

**Supplementary Figure S2**. GO enrichment terms.

**Supplementary Figure S3**. RepeatExplorer clustering summary in Musaceae species.

**Supplementary Figure S4**. LTR retroelement trees EGL and MAC.

**Supplementary Figure S5**. Gypsy and Copia insertion times in *Musa* and *E. glaucum*.

**Supplementary Figure S6:** Egcen FISH to *Musa* chromosomes.

**Supplementary Figure S7**. EgCen and *Nanica* in assemblies of *E. glaucum* and *Musa*.

**Supplementary Figure S8**. Synteny of *E. glaucum* with *Musa* A and B genome.

**Supplementary Figure S9**. Dot plots of individual chromosomes.

**Supplementary Figure S10**. Inversions on chromosome 5.

**Supplementary Figure S11**. Length distribution of ONT reads.

**Supplementary Figure S12**. Hi-C interaction contact map.

**Supplementary Table S1**. Contig statistics based on assembly of ONT sequencing data.

**Supplementary Table S2**. Chromosome lengths and number of contigs anchored in *Ensete glaucum* assembly.

**Supplementary Table S3**. Quality assessment of the gene annotation of *Ensete glaucum* using BUSCOs v5.

**Supplementary Table S4**. Complete gene list: homology and GO.

**Supplementary Table S5**. Statistics for shared orthogroups (OG) and gene clustering among *E. glaucum, E. ventricosum, Musa acuminata, M. balbisiana*, and *M. schizocarpa* genomes.

**Supplementary Table S6**. Positively selected genes and their annotation.

**Supplementary Table S7**. Result of gene family size change analysis.

**Supplementary Table S8. (a)** Top 20 GO molecular function enrichments for *E. glaucum* and shared *E. glaucum/E. ventricosum* gene families; (b) Top 20 GO biological pathway enrichments for *E. glaucum* and shared *E. glaucum/E. ventricosum* gene families.

**Supplementary Table S9**. Comparison of transcriptional factors between *Ensete glaucum* and *Musa acuminata*.

**Supplementary Table S10**. Transposable elements and other repeat proportions comparison in assembly (RepeatMasker).

**Supplementary Table S11**. Repeat content (RepeatExplorer) comparison between different Musaceae genomes.

**Supplementary Table S12**. Abundance of major tandemly repeated DNA repeats in Illumina raw reads.

**Supplementary Table S13**. Inferred centromere positions from locations of interrupted tandem arrays of the Egcen centromeric sequence on the chromosome assemblies.

**Supplementary Table S14**. Comparative survey of microsatellite sequences in *Ensete glaucum* genome with other sister species.

giac027_GIGA-D-21-00354_Original_Submission

giac027_GIGA-D-21-00354_Revision_1

giac027_Response_to_Reviewer_Comments_Original_Submission

giac027_Reviewer_1_Report_Original_Submission Boas Pucker -- 11/27/2021 Reviewed

giac027_Reviewer_1_Report_Revision_1 Boas Pucker -- 2/4/2022 Reviewed

giac027_Reviewer_2_Report_Original_Submission Ning Jiang, Ph. D -- 12/9/2021 Reviewed

giac027_Supplemental_Files

## Abbreviations

BLAST: Basic Local Alignment Search Tool; bp: base pairs; BUSCO: Benchmarking Universal Single-Copy Orthologs; BWA: Burrows-Wheeler Aligner; CAFE: Computational Analysis of gene Family Evolution; CTAB: cetyl trimethylammonium bromide; DAPI: 4´,6-diamidino-2-phenylindole; Egcen: *Ensete glaucum* centromere sequence; FISH: fluorescence *in situ*hybridization; Gb: gigabase pairs; GC: guanine-cytosine; GeMoMa: Gene Model Mapper; GO: gene ontogeny; Hi-C: high-throughput chromosome conformation capture; HMM: hidden Markov model; ITS: internal transcribed spacer of rDNA; kb: kilobase pairs; KEGG: Kyoto Encyclopedia of Genes and Genomes; LACHESIS: Ligating Adjacent Chromatin Enables Scaffolding In Situ; LINE: long interspersed nucleotide element; LTR: long terminal repeat; Mb: megabase pairs; ML: maximum likelihood; Mya: million years ago; NCBI: National Center for Biotechnology Information; NR: RefSeq non-redundant proteins; NOR: nucleolar organizing region; NTS: non-transcribed spacer of rDNA; ONT: Oxford Nanopore Technologies; PAML: Phylogenetic Analysis by Maximum Likelihood; PASA: Program to Assemble Spliced Alignments; rDNA: ribosomal DNA; RNA-seq: RNA sequencing; RT: reverse transcriptase; SRA: Sequence Read Archive; SSR: simple sequence repeat; TE: transposable element; TF: transcription factor; WGD: whole-genome duplication.

## Consent for Publication

The origin of *E. glaucum* plants is given in Materials and Methods. They were collected in China and conserved in the South China Botanical Garden, Chinese Academy of Sciences, with appropriate agreements. No live material was exported out of the country. Other plants for chromosome preparations were obtained from the International Transit Centre, ITC genebank, with official Standard Material Transfer Agreement (SMTA), acknowledged in the manuscript.

## Competing Interests

The authors declare that they have no competing interests.

## Funding

This work was supported by grants from National Science Foundation of China> (32070359), Guangdong Basic and Applied Basic Research Foundation (2021A1515012410), Overseas Distinguished Scholar Project of SCBG (Y861041001), and Undergraduate Innovation Training Program of Chinese Academy of Sciences (KCJH-80107-2020-004-97). M.R. acknowledges the support of the CGIAR Research Program on Roots, Tubers and Bananas (RTB). MKB, TS and PHH acknowledge partial support of GCRF Foundation Awards for Global Agricultural and Food Systems Research, entitled, ‘Modelling and genomics resources to enhance exploitation of the sustainable and diverse Ethiopian starch crop enset and support livelihoods’ [Grant No. BB/P02307X/1].

## Authors' Contributions

Q.L. and J.S.H.H. designed the project and with M.R. and Z.W. contributed to project coordination. Z.W. and Q.H. collected samples and conducted DNA and RNA extraction. T.S. and J.S.H.H. conducted FISH experiments. Z.W. carried out genome assemblies; Z.W., M.R., G.D., and M.K.B. conducted gene annotation and comparative genomic analyses; Z.W., T.S., J.S.H.H., and F.C.B. conducted repetitive sequence analysis. All authors contributed to writing and editing the manuscript.

## References

[1] Wu Z, Raven PH, Hong D. Musaceae. In: Flora of China. Science Press: Beijing and Missouri Botanical Garden Press: St. Louis. 2000:297–8.

[2] Borrell JS, Biswas MK, Goodwin M, et al. Enset in Ethiopia: a poorly characterized but resilient starch staple. Ann Bot. 2019;123(5):747–66. 30715125 10.1093/aob/mcy214 PMC6526316

[3] Zhao T, Zwaenepoel A, Xue J-Y, et al. Whole-genome microsynteny-based phylogeny of angiosperms. Nat Commun. 2021;12(1):3498. 34108452 10.1038/s41467-021-23665-0 PMC8190143

[4] Christelová P, Valárik M, Hřibová E, et al. A multi gene sequence-based phylogeny of the Musaceae (banana) family. BMC Evol Biol. 2011;11(1):doi:10.1186/1471-2148-11-103. PMC3102628 21496296

[5] Janssens SB, Vandelook F, De Langhe E, et al. Evolutionary dynamics and biogeography of Musaceae reveal a correlation between the diversification of the banana family and the geological and climatic history of Southeast Asia. New Phytol. 2016;210(4):1453–65. 26832306 10.1111/nph.13856 PMC5066818

[6] Cheesman EE. Classification of the bananas: the genus *Ensete* Horan. Kew Bull. 1947;2(2):97–106.

[7] Simmonds NW. Notes on banana taxonomy. Kew Bull. 1960;14(2):198–212.

[8] Li H-W . The Musaceae of Yunnan[J]. Acta Phytotaxonomica Sinica. 1978;16(3):54–64.

[9] Ochiai Y. From forests to homegardens: a case study of *Ensete glaucum* in Myanmar and Laos. Tropics. 2012;21(2):doi:10.3759/TROPICS.21.59.

[10] Song J-J, Liao J-P, Tang Y-J, et al. Chromosome numbers in Orchidantha (Lowiaceae) and their biogeographic and systematic implications. Ann Bot Fennici. 2004;41:429–33.

[11] Majumdar K, Sarkar A, Deb D, et al. Distribution record of *Ensete glaucum* (Roxb.) Cheesm. (Musaceae) in Tripura, Northeast India: a rare wild primitive banana. Asian J Conserv Biol. 2013;2:164–7.

[12] Yang Q-S, Gao J, He W-D, et al. Comparative transcriptomics analysis reveals difference of key gene expression between banana and plantain in response to cold stress. BMC Genomics. 2015;16(1):doi:10.1186/s12864-015-1551-z. PMC4461995 26059100

[13] Belser C, Istace B, Denis E, et al. Chromosome-scale assemblies of plant genomes using nanopore long reads and optical maps. Nat Plants. 2018;4(11):879–87. 30390080 10.1038/s41477-018-0289-4

[14] D'Hont A, Denoeud F, Aury J-M, et al. The banana (*Musa acuminata*) genome and the evolution of monocotyledonous plants. Nature. 2012;488(7410):213. 22801500 10.1038/nature11241

[15] Wang Z, Miao H, Liu J, et al. *Musa balbisiana* genome reveals subgenome evolution and functional divergence. Nat Plants. 2019;5(8):810–21. 31308504 10.1038/s41477-019-0452-6 PMC6784884

[16] Martin G, Baurens F-C, Droc G, et al. Improvement of the banana “*Musa acuminata*” reference sequence using NGS data and semi-automated bioinformatics methods. BMC Genomics. 2016;17(1):doi:10.1186/s12864-016-2579-4. PMC4793746 26984673

[17] Belser C, Baurens F-C, Noel B, et al. Telomere-to-telomere gapless chromosomes of banana using nanopore sequencing. Commun Biol. 2021;4(1):doi:10.1038/s42003-021-02559-3. PMC8423783 34493830

[18] Droc G, Lariviere D, Guignon V, et al. The Banana Genome Hub. Database (Oxford). 2013:doi:10.1093/database/bat035. PMC3662865 23707967

[19] Yemataw Z, Muzemil S, Ambachew D, et al. Genome sequence data from 17 accessions of *Ensete ventricosum*, a staple food crop for millions in Ethiopia. Data Brief. 2018;18:285–93. 29896517 10.1016/j.dib.2018.03.026 PMC5996239

[20] Harrison J, Moore KA, Paszkiewicz K, et al. A draft genome sequence for *Ensete ventricosum*, the drought-tolerant “tree against hunger.” Agronomy. 2014;4(1):13–33.

[21] Simão FA, Waterhouse RM, Ioannidis P, et al. BUSCO: assessing genome assembly and annotation completeness with single-copy orthologs. Bioinformatics. 2015;31(19):3210–2. 26059717 10.1093/bioinformatics/btv351

[22] Pucker B. Mapping-based genome size estimation. bioRxiv 2019:doi:10.1101/607390. PMC12079912 40369445

[23] Bartos J, Alkhimova O, Dolezelová M, et al. Nuclear genome size and genomic distribution of ribosomal DNA in *Musa* and *Ensete* (Musaceae): taxonomic implications. Cytogenet Genome Res. 2005;109(1-3):50–7. 15753558 10.1159/000082381

[24] Wang R, Yang Y, Jing Y, et al. Molecular mechanisms of mutualistic and antagonistic interactions in a plant-pollinator association. Nat Ecol Evol. 2021;5(7):974–-86. 34002050 10.1038/s41559-021-01469-1

[25] González AV, Gómez-Silva V, Ramírez MJ, et al. Meta-analysis of the differential effects of habitat fragmentation and degradation on plant genetic diversity. Conserv Biol. 2020;34(3):711–20. 31605401 10.1111/cobi.13422

[26] Liu A-Z, Kress WJ, Wang HF, et al. Insect pollination of *Musella* (Musaceae), a monotypic genus endemic to Yunnan, China. Plant Syst Evol. 2002;235(1):135–46.

[27] Sardos J, Breton C, Perrier X, et al. Wild to domesticates: genomes of edible diploid bananas hold traces of several undefined genepools. bioRxiv 2021:doi:10.1101/2021.01.29.428762.

[28] Martin G, Cardi C, Sarah G, et al. Genome ancestry mosaics reveal multiple and cryptic contributors to cultivated banana. Plant J. 2020;102(5):1008–25. 31930580 10.1111/tpj.14683 PMC7317953

[29] Maughan PJ, Lee R, Walstead R, et al. Genomic insights from the first chromosome-scale assemblies of oat (*Avena* spp.) diploid species. BMC Biol. 2019;17(1):doi:10.1186/s12915-019-0712-y. PMC6874827 31757219

[30] Marrano A, Britton M, Zaini PA, et al. High-quality chromosome-scale assembly of the walnut (*Juglans regia* L.) reference genome. Gigascience. 2020;9(5):doi:10.1093/gigascience/giaa050. PMC7238675 32432329

[31] Yang X, Kang M, Yang Y, et al. A chromosome-level genome assembly of the Chinese tupelo *Nyssa sinensis*. Sci Data. 2019;6(1): doi:10.1038/s41597-019-0296-y. PMC6877568 31767848

[32] Voillemot M, Pannell JR. Inbreeding depression is high in a self-incompatible perennial herb population but absent in a self-compatible population showing mixed mating. Ecol Evol. 2017;7(20):8535–44. 29075469 10.1002/ece3.3354 PMC5648656

[33] Sun G, Xu Y, Liu H, et al. Large-scale gene losses underlie the genome evolution of parasitic plant *Cuscuta australis*. Nat Commun. 2018;9(1):doi:10.1038/s41467-018-04721-8. PMC6041341 29992948

[34] Redwan RM, Saidin A, Kumar SV. The draft genome of MD-2 pineapple using hybrid error correction of long reads. DNA Res. 2016;23(5):427–39. 27374615 10.1093/dnares/dsw026 PMC5066169

[35] Franco-Zorrilla JM, López-Vidriero I, Carrasco JL, et al. DNA-binding specificities of plant transcription factors and their potential to define target genes. Proc Natl Acad Sci U S A. 2014;111(6):2367–72. 24477691 10.1073/pnas.1316278111 PMC3926073

[36] Cenci A, Guignon V, Roux N, et al. Genomic analysis of NAC transcription factors in banana (*Musa acuminata*) and definition of NAC orthologous groups for monocots and dicots. Plant Mol Biol. 2014;85(1-2):63–80. 24570169 10.1007/s11103-013-0169-2 PMC4151281

[37] Xiao Y-Y, Kuang J-F, Qi X-N, et al. A comprehensive investigation of starch degradation process and identification of a transcriptional activator MabHLH6 during banana fruit ripening. Plant Biotechnol J. 2018;16(1):151–64. 28500777 10.1111/pbi.12756 PMC5785343

[38] Lerat E. Identifying repeats and transposable elements in sequenced genomes: how to find your way through the dense forest of programs. Heredity (Edinb). 2010;104(6):520–33. 19935826 10.1038/hdy.2009.165

[39] Novák P, Neumann P, Macas J. Global analysis of repetitive DNA from unassembled sequence reads using RepeatExplorer2. Nat Protoc. 2020;15(11):3745–76. 33097925 10.1038/s41596-020-0400-y

[40] Wu W, Yang Y-L, He W-M, et al. Whole genome sequencing of a banana wild relative *Musa itinerans* provides insights into lineage-specific diversification of the *Musa* genus. Sci Rep. 2016;6(1):doi:10.1038/srep31586. PMC4987669 27531320

[41] Biscotti MA, Olmo E, Heslop-Harrison JSP. Repetitive DNA in eukaryotic genomes. Chromosome Res. 2015;23(3):415–20. 26514350 10.1007/s10577-015-9499-z

[42] Heslop-Harrison JSP, Schwarzacher T. Organisation of the plant genome in chromosomes. Plant J. 2011;66(1):18–33. 21443620 10.1111/j.1365-313X.2011.04544.x

[43] Čížková J, Hřibová E, Humplíková L, et al. Molecular analysis and genomic organization of major DNA satellites in banana (*Musa* spp.). PLoS One. 2013;8(1):e54808. 23372772 10.1371/journal.pone.0054808 PMC3553004

[44] Suntronpong A, Kugou K, Masumoto H, et al. CENP-B box, a nucleotide motif involved in centromere formation, occurs in a New World monkey. Biol Lett. 2016;12(3):20150817. 27029836 10.1098/rsbl.2015.0817 PMC4843215

[45] Aragón-Alcaide L, Miller T, Schwarzacher T, et al. A cereal centromeric sequence. Chromosoma. 1996;105(5):261–8. 8939818 10.1007/BF02524643

[46] Heslop-Harrison JS, Murata M, Ogura Y, et al. Polymorphisms and genomic organization of repetitive DNA from centromeric regions of *Arabidopsis* chromosomes. Plant Cell. 1999;11(1):31–42. 9878630 10.1105/tpc.11.1.31 PMC144094

[47] Lermontova I, Sandmann M, Mascher M, et al. Centromeric chromatin and its dynamics in plants. Plant J. 2015;83(1):4–17. 25976696 10.1111/tpj.12875

[48] Biswas MK, Natarajan S, Biswas D, et al. LSAT: Liliaceae Simple Sequences Analysis Tool, a web server. Bioinformation. 2018;14(4):181–2. 29983488 10.6026/97320630014181 PMC6016758

[49] Biswas MK, Darbar JN, Borrell JS, et al. The landscape of microsatellites in the enset (*Ensete ventricosum*) genome and web–based marker resource development. Sci Rep. 2020;10(1):15312. 32943659 10.1038/s41598-020-71984-x PMC7498607

[50] Liu Q, Li X, Zhou X, et al. The repetitive DNA landscape in *Avena*(Poaceae): chromosome and genome evolution defined by major repeat classes in whole-genome sequence reads. BMC Plant Biol. 2019;19(1):226. 31146681 10.1186/s12870-019-1769-z PMC6543597

[51] Goffová I, Fajkus J. The rDNA loci—intersections of replication, transcription, and repair pathways. Int J Mol Sci. 2021;22(3):1302. 33525595 10.3390/ijms22031302 PMC7865372

[52] Tulpová Z, Kovařík A, Toegelová H, et al. Anatomy, transcription dynamics and evolution of wheat ribosomal RNA loci deciphered by a multi-omics approach. bioRxiv 2021:doi:10.1101/2020.08.29.273623. PMC12806993 35092350

[53] Osuji JO, Crouch J, Harrison G, et al. Molecular cytogenetics of *Musa* species, cultivars and hybrids: location of 18S-5.8S-25S and 5S rDNA and telomere-like sequences. Ann Bot. 1998;82(2): 243–8.

[54] Baurens F-C, Noyer J-L, Lanaud C, et al. Assessment of a species-specific element (Brep 1) in banana. Theor Appl Genet. 1997;95:922–31.

[55] Garcia S, Wendel JF, Borowska-Zuchowska N, et al. The utility of graph clustering of 5S ribosomal DNA hhomoeologs in plant allopolyploids, homoploid hybrids, and cryptic introgressants. Front Plant Sci. 2020;11:doi:10.3389/fpls.2020.00041. PMC7025596 32117380

[56] Castilho A, Heslop-Harrison JS. Physical mapping of 5S and 18S-25S rDNA and repetitive DNA sequences in *Aegilops umbellulata*. Genome. 1995;38(1):91–6. 18470155 10.1139/g95-011

[57] Dubcovsky J, Dvorák J. Ribosomal RNA multigene loci: nomads of the Triticeae genomes. Genetics. 1995;140(4):1367–77. 7498776 10.1093/genetics/140.4.1367 PMC1206700

[58] Bandi V, Gutwin C. Interactive exploration of genomic conservation. In: 46th Graphics Interface Conference on Proceedings of Graphics Interface 2020. Waterloo, Canada: Canadian Human-Computer Communications Society; 2020:74–83.

[59] Li W, Challa GS, Zhu H, et al. Recurrence of chromosome rearrangements and reuse of DNA breakpoints in the evolution of the Triticeae genomes. G3 (Bethesda). 2016;6(12):3837–47. 27729435 10.1534/g3.116.035089 PMC5144955

[60] Lewin HA, Richards S, Lieberman Aiden E, et al. The Earth BioGenome Project 2020: Starting the clock. Proc Natl Acad Sci U S A. 2022;119(4):e2115635118. 35042800 10.1073/pnas.2115635118 PMC8795548

[61] GBIF.org: GBIF. 10.15468/dl.f9meez. (2021). Accessed 2021 April 25.

[62] Kew Science. Plants of the World Online. http://www.plantsoftheworldonline.org. Accessed 3 September 2021.

[63] Bolger AM, Lohse M, Usadel B. Trimmomatic: a flexible trimmer for Illumina sequence data. Bioinformatics. 2014;30(15):2114–20. 24695404 10.1093/bioinformatics/btu170 PMC4103590

[64] Andrews S. Babraham Bioinformatics—FastQC A Quality Control Tool for High Throughput Sequence Data. https://www.bioinformatics.babraham.ac.uk/projects/fastqc/. Accessed: November, 20, 2019.

[65] Chen S, Zhou Y, Chen Y, et al. fastp: an ultra-fast all-in-one FASTQ preprocessor. Bioinformatics. 2018;34(17):i884–90. 30423086 10.1093/bioinformatics/bty560 PMC6129281

[66] Belton J-M, McCord RP, Gibcus JH, et al. Hi-C: a comprehensive technique to capture the conformation of genomes. Methods. 2012;58(3):268–76. 22652625 10.1016/j.ymeth.2012.05.001 PMC3874846

[67] Belaghzal H, Dekker J, Gibcus JH. Hi-C 2.0: An optimized Hi-C procedure for high-resolution genome-wide mapping of chromosome conformation. Methods. 2017;123. 10.1016/j.ymeth.2017.04.004 PMC5522765 28435001

[68] NextDenovo. https://github.com/Nextomics/NextDenovo. Accessed 2019 Oct 26.

[69] Liu H, Wu S, Li A, et al. SMARTdenovo: a de novo assembler using long noisy reads. Gigabyte. 2021;1:doi:10.46471/gigabyte.15. PMC9632051 36824332

[70] Vaser R, Sović I, Nagarajan N, et al. Fast and accurate de novo genome assembly from long uncorrected reads. Genome Res. 2017;27(5):737–46. 28100585 10.1101/gr.214270.116 PMC5411768

[71] Li H, Durbin R. Fast and accurate long-read alignment with Burrows-Wheeler transform. Bioinformatics. 2010;26(5):589–95. 20080505 10.1093/bioinformatics/btp698 PMC2828108

[72] Hu J, Fan J, Sun Z, et al. NextPolish: a fast and efficient genome polishing tool for long-read assembly. Bioinformatics. 2020;36(7):2253–5. 31778144 10.1093/bioinformatics/btz891

[73] Walker BJ, Abeel T, Shea T, et al. Pilon: an integrated tool for comprehensive microbial variant detection and genome assembly improvement. PLoS One. 2014;9(11):e112963. 25409509 10.1371/journal.pone.0112963 PMC4237348

[74] Langmead B, Salzberg SL. Fast gapped-read alignment with Bowtie 2. Nat Methods. 2012;9(4):357–9. 22388286 10.1038/nmeth.1923 PMC3322381

[75] Servant N, Varoquaux N, Lajoie BR, et al. HiC-Pro: an optimized and flexible pipeline for Hi-C data processing. Genome Biol. 2015;16(1):doi:10.1186/s13059-015-0831-x. PMC4665391 26619908

[76] Burton JN, Adey A, Patwardhan RP, et al. Chromosome-scale scaffolding of de novo genome assemblies based on chromatin interactions. Nat Biotechnol. 2013;31(12):1119–25. 24185095 10.1038/nbt.2727 PMC4117202

[77] Grabherr MG, Haas BJ, Yassour M, et al. Trinity: reconstructing a full-length transcriptome without a genome from RNA-Seq data. Nat Biotechnol. 2011;29(7):644–52. 21572440 10.1038/nbt.1883 PMC3571712

[78] Haas BJ, Delcher AL, Mount SM, et al. Improving the *Arabidopsis* genome annotation using maximal transcript alignment assemblies. Nucleic Acids Res. 2003;31(19):5654–66. 14500829 10.1093/nar/gkg770 PMC206470

[79] Marçais G, Kingsford C. A fast, lock-free approach for efficient parallel counting of occurrences of k-mers. Bioinformatics. 2011;27(6):764–70. 21217122 10.1093/bioinformatics/btr011 PMC3051319

[80] Sun H, Ding J, Piednoël M, et al. findGSE: estimating genome size variation within human and *Arabidopsis* using k-mer frequencies. Bioinformatics. 2018;34(4):550–57. 29444236 10.1093/bioinformatics/btx637

[81] Ranallo-Benavidez TR, Jaron KS, Schatz MC. GenomeScope 2.0 and Smudgeplot for reference-free profiling of polyploid genomes. Nat Commun. 2020;11(1):doi:10.1038/s41467-020-14998-3. PMC7080791 32188846

[82] Dobin A, Davis CA, Schlesinger F, et al. STAR: ultrafast universal RNA-seq aligner. Bioinformatics. 2013;29(1):15–21. 23104886 10.1093/bioinformatics/bts635 PMC3530905

[83] Brůna T, Hoff KJ, Lomsadze A, et al. BRAKER2: automatic eukaryotic genome annotation with GeneMark-EP+ and AUGUSTUS supported by a protein database. NAR Genom Bioinform. 2021;3(1):lqaa108. 33575650 10.1093/nargab/lqaa108 PMC7787252

[84] Lomsadze A, Burns PD, Borodovsky M. Integration of mapped RNA-Seq reads into automatic training of eukaryotic gene finding algorithm. Nucleic Acids Res. 2014;42(15):e119. 24990371 10.1093/nar/gku557 PMC4150757

[85] Stanke M, Diekhans M, Baertsch R, et al. Using native and syntenically mapped cDNA alignments to improve de novo gene finding. Bioinformatics. 2008;24(5):637–44. 18218656 10.1093/bioinformatics/btn013

[86] Campbell MS, Law M, Holt C, et al. MAKER-P: a tool kit for the rapid creation, management, and quality control of plant genome annotations. Plant Physiol. 2014;164(2):513–24. 24306534 10.1104/pp.113.230144 PMC3912085

[87] Keilwagen J, Hartung F, Grau J. GeMoMa: Homology-based gene prediction utilizing intron position conservation and RNA-seq data. Methods Mol Biol. 2019;1962:161–77. 31020559 10.1007/978-1-4939-9173-0_9

[88] Haas BJ, Salzberg SL, Zhu W, et al. Automated eukaryotic gene structure annotation using EVidenceModeler and the Program to Assemble Spliced Alignments. Genome Biol. 2008;9(1):R7. 18190707 10.1186/gb-2008-9-1-r7 PMC2395244

[89] Camacho C, Coulouris G, Avagyan V, et al. BLAST+: architecture and applications. BMC Bioinformatics. 2009;10:421. 20003500 10.1186/1471-2105-10-421 PMC2803857

[90] Magrane M, UniProt Consortium. UniProt Knowledgebase: a hub of integrated protein data. Database (Oxford). 2011;2011:doi:10.1093/database/bar009. PMC3070428 21447597

[91] Zdobnov EM, Apweiler R. InterProScan - an integration platform for the signature-recognition methods in InterPro. Bioinformatics. 2001;17(9):847–8. 11590104 10.1093/bioinformatics/17.9.847

[92] Conesa A, Götz S, García-Gómez JM, et al. Blast2GO: a universal tool for annotation, visualization and analysis in functional genomics research. Bioinformatics. 2005;21(18):3674–6. 16081474 10.1093/bioinformatics/bti610

[93] Droc G: ensete_annotation. https://github.com/gdroc/ensete_annotation. (2021). Accessed 25 October 2021.

[94] Emms DM, Kelly S. OrthoFinder: solving fundamental biases in whole genome comparisons dramatically improves orthogroup inference accuracy. Genome Biol. 2015;16(1):doi:10.1186/s13059-015-0721-2. PMC4531804 26243257

[95] Buchfink B, Xie C, Huson DH. Fast and sensitive protein alignment using DIAMOND. Nat Methods. 2015;12(1):59–60. 25402007 10.1038/nmeth.3176

[96] Lex A, Gehlenborg N, Strobelt H, et al. UpSet: visualization of intersecting sets. IEEE Trans Visual Comput Graphics. 2014;20(12):1983–92. 10.1109/TVCG.2014.2346248 PMC4720993 26356912

[97] Alexa A, Rahnenfuhrer J. topGO: enrichment analysis for gene ontology. R package version 2.24.0. 2010. https://bioconductor.org/packages/release/bioc/html/topGO.html. Accessed: October, 9, 2020.

[98] Hazzouri KM, Gros-Balthazard M, Flowers JM, et al. Genome-wide association mapping of date palm fruit traits. Nat Commun. 2019;10(1):4680. 31615981 10.1038/s41467-019-12604-9 PMC6794320

[99] Ouyang S, Zhu W, Hamilton J, et al. The TIGR Rice Genome Annotation Resource: improvements and new features. Nucleic Acids Res. 2007;35(Database issue):D883–7. 17145706 10.1093/nar/gkl976 PMC1751532

[100] Goodstein DM, Shu S, Howson R, et al. Phytozome: a comparative platform for green plant genomics. Nucleic Acids Res. 2012;40(D1):D1178–86. 22110026 10.1093/nar/gkr944 PMC3245001

[101] Han MV, Thomas GWC, Lugo-Martinez J, et al. Estimating gene gain and loss rates in the presence of error in genome assembly and annotation using CAFE 3. Mol Biol Evol. 2013;30(8):1987–97. 23709260 10.1093/molbev/mst100

[102] Zheng Y, Jiao C, Sun H, et al. iTAK: a program for genome-wide prediction and classification of plant transcription factors, transcriptional regulators, and protein kinases. Mol Plant. 2016;9(12):1667–70. 27717919 10.1016/j.molp.2016.09.014

[103] Sun P, Jiao B, Yang Y, et al. WGDI: A user-friendly toolkit for evolutionary analyses of whole-genome duplications and ancestral karyotypes. bioRxiv 2021:doi:10.1101/2021.04.29.441969. 36307977

[104] Li H. Minimap2: pairwise alignment for nucleotide sequences. Bioinformatics. 2018;34(18):3094–100. 29750242 10.1093/bioinformatics/bty191 PMC6137996

[105] Cabanettes F, Klopp C. D-GENIES: dot plot large genomes in an interactive, efficient and simple way. PeerJ. 2018;6:doi:10.7717/peerj.4958. PMC5991294 29888139

[106] Wang Y, Tang H, DeBarry JD, et al. MCScanX: a toolkit for detection and evolutionary analysis of gene synteny and collinearity. Nucleic Acids Res. 2012;40(7):e49. 22217600 10.1093/nar/gkr1293 PMC3326336

[107] Amselem J, Cornut G, Choisne N, et al. RepetDB: a unified resource for transposable element references. Mob DNA. 2019;10(1):doi:10.1186/s13100-019-0150-y. PMC6350395 30719103

[108] Belser C . Pahang-associated-data. GitHub. https://github.com/institut-de-genomique/Pahang-associated-data. (2021). Accessed 25 October 2021.

[109] Ou S, Su W, Liao Y, et al. Benchmarking transposable element annotation methods for creation of a streamlined, comprehensive pipeline. Genome Biol. 2019;20(1):275. 31843001 10.1186/s13059-019-1905-y PMC6913007

[110] Ellinghaus D, Kurtz S, Willhoeft U. LTRharvest, an efficient and flexible software for de novo detection of LTR retrotransposons. BMC Bioinformatics. 2008;9(1):doi:10.1186/1471-2105-9-18. PMC2253517 18194517

[111] Ou S, Jiang N. LTR_retriever: a highly accurate and sensitive program for identification of long terminal repeat retrotransposons. Plant Physiol. 2018;176(2):doi:10.1186/1471-2105-9-18. PMC5813529 29233850

[112] Xu Z, Wang H. LTR_FINDER: an efficient tool for the prediction of full-length LTR retrotransposons. Nucleic Acids Res. 2007;35(Web Server):W265–8. 17485477 10.1093/nar/gkm286 PMC1933203

[113] Su W, Gu X, Peterson T. TIR-Learner, a new ensemble method for TIR transposable element annotation, provides evidence for abundant new transposable elements in the maize genome. Mol Plant. 2019;12(3):447–60. 30802553 10.1016/j.molp.2019.02.008

[114] Shi J, Liang C. Generic Repeat Finder: a high-sensitivity tool for genome-wide *de novo* repeat detection. Plant Physiol. 2019;180(4):1803–15. 31152127 10.1104/pp.19.00386 PMC6670090

[115] Xiong W, He L, Lai J, et al. HelitronScanner uncovers a large overlooked cache of Helitron transposons in many plant genomes. Proc Natl Acad Sci U S A. 2014;111(28):10263–8. 24982153 10.1073/pnas.1410068111 PMC4104883

[116] Flynn JM, Hubley R, Goubert C, et al. RepeatModeler2 for automated genomic discovery of transposable element families. Proc Natl Acad Sci U S A. 2020;117(17):9451–7. 32300014 10.1073/pnas.1921046117 PMC7196820

[117] Flutre T, Duprat E, Feuillet C, et al. Considering transposable element diversification in de novo annotation approaches. PLoS One. 2011;6(1):e16526. 21304975 10.1371/journal.pone.0016526 PMC3031573

[118] Fu L, Niu B, Zhu Z, et al. CD-HIT: accelerated for clustering the next-generation sequencing data. Bioinformatics. 2012;28(23):3150–2. 23060610 10.1093/bioinformatics/bts565 PMC3516142

[119] Zhang R-G, Wang Z-X, Ou S, et al. TEsorter: lineage-level classification of transposable elements using conserved protein domains. bioRxiv 2019:doi:10.1101/800177.

[120] Edgar RC. MUSCLE: multiple sequence alignment with high accuracy and high throughput. Nucleic Acids Res. 2004;32(5):1792–7. 15034147 10.1093/nar/gkh340 PMC390337

[121] Ma J, Bennetzen JL. Rapid recent growth and divergence of rice nuclear genomes. Proc Natl Acad Sci U S A. 2004;101(34):12404–10. 15240870 10.1073/pnas.0403715101 PMC515075

[122] Wang Z . LTR-insertion-time-estimation. https://github.com/wangziwei08/LTR-insertion-time-estimation/. (2021). Accessed 25 October 2021.

[123] Schwarzacher T, Heslop-Harrison JS. Practical In Situ Hybridization. Oxford, UK: BIOS Scientific Publishers Ltd; 2000.

[124] Ruas M, Guignon V, Sempere G, et al. MGIS: managing banana (*Musa* spp.) genetic resources information and high-throughput genotyping data. Database (Oxford). 2017;2017:doi:10.1093/database/bax046. PMC5502358 29220435

[125] Gerlach WL, Bedbrook JR. Cloning and characterization of ribosomal RNA genes from wheat and barley. Nucleic Acids Res. 1979;7(7):1869–85. 537913 10.1093/nar/7.7.1869 PMC342353

[126] Manchester SR, Kress WJ. Fossil bananas (Musaceae): *Ensete oregonense* sp. nov. from the Eocene of western North America and its phytogeographic significance. Am J Bot. 1993;80(11):1264–72.

[127] Wang Z, Rouard M, Biswas MK, et al. Supporting data for “A chromosome-level reference genome of *Ensete glaucum* gives insight into diversity and chromosomal and repetitive sequence evolution in the Musaceae”. GigaScience Database. 2022; 10.5524/102198. PMC9055855 35488861

