# Supplementary material for: A chromosome-level reference genome of Ensete glaucum gives insight into diversity and chromosomal and repetitive sequence evolution in the Musaceae
Source: Gigascience. 2022 Apr 30;11:giac027. doi: 10.1093/gigascience/giac027 (PMC9055855; doi:10.1093/gigascience/giac027)
Supplement: giac027_GIGA-D-21-00354_Revision_1 [file giac027_giga-d-21-00354_revision_1.pdf]

GigaScience

A chromosome-level reference genome of Ensete glaucum gives insight into diversity, chromosomal and repetitive sequence evolution in the Musaceae

--Manuscript Draft--

**Manuscript Number:**	GIGA-D-21-00354R1
**Full Title:**	A chromosome-level reference genome of Ensete glaucum gives insight into diversity, chromosomal and repetitive sequence evolution in the Musaceae
**Article Type:**	Data Note

Funding Information:

National Natural Science Foundation of China (32070359)	Dr Qing Liu
CGIAR (Roots, Tubers and Bananas)	Dr Mathieu Rouard
Guangdong Basic and Applied Basic Research Foundation (2021A1515012410)	Dr Qing Liu
Overseas Distinguished Scholar Project of SCBG (Y861041001)	Professor JS (Pat) Heslop-Harrison
Undergraduate Innovation Training Program of Chinese Academy of Sciences (KCJH-80107-2020-004-97)	Dr Qing Liu

Abstract:

Abstract

Background: Ensete glaucum (2 n = 2 x = 18) is a giant herbaceous monocotyledonous plant in the small Musaceae family along with banana (Musa). A high-quality reference genome sequence assembly of E. glaucum offers a vital genomic resource for functional and evolutionary studies of Ensete , the Musaceae, and more widely in the Zingiberales.

Findings: Using a combination of Illumina and Oxford Nanopore Technologies (ONT) sequencing, genome-wide chromosome conformation capture (Hi-C), and RNA survey sequence, supported by bioinformatic analysis of raw reads and molecular cytogenetic fluorescent in situ hybridization, we report a high-quality 481.5Mb genome assembly with 9 pseudo-chromosomes and 36,836 genes. A total of 55% of the genome is composed of repetitive sequences with predominantly LTR-retroelements (37%) and DNA transposons (7%). The 5S and 45S rDNA were each present at one locus, on chromosomes 5 and 6 respectively. The 5S rDNA had an exceptionally long monomer length of 1,056 bp, more than twice that of the monomers at multiple loci in Musa . A tandemly repeated satellite (1.1% of the genome, with no similar sequence in Musa) was present around all nine centromeres, together with a few copies of a LINE retroelement found at higher frequency at Musa centromeres. The assembly, including centromeric positions, enabled us to characterize in detail the chromosomal rearrangements occurring between E. glaucum and the x = 11 species of Musa . Only one E. glaucum chromosome has the same gene content as M. acuminata although rearranged. Three E. glaucum chromosomes represent part of only one M. acuminata chromosome, while the remaining chromosomes involve multiple, complex but clearly defined evolutionary rearrangements in the change between x = 9 and x = 11.

Conclusions: The advance towards a Musaceae pangenome including E. glaucum, tolerant of extreme environments, makes a complete set of gene alleles, copy number variation (CNV), and a reference for structural variation available for crop breeding and understanding environmental responses . The chromosome-scale genome assembly show the nature of chromosomal fusion and translocation events during speciation, and features of rapid repetitive DNA change in terms of copy number, sequence and genomic location, critical to understanding its role in diversity and s evolution.

Corresponding Author: Ziwei Wang
South China Botanical Garden

Powered by Editorial Manager® and ProduXion Manager® from Aries Systems Corporation

	Guangzhou, CHINA
**Corresponding Author Secondary Information:**	
**Corresponding Author's Institution:**	South China Botanical Garden
**Corresponding Author's Secondary Institution:**	
**First Author:**	Ziwei Wang
**First Author Secondary Information:**	
**Order of Authors:**	Ziwei Wang
	Mathieu Rouard
	Manosh Kumar Biswas
	Gaetan Droc
	Dongli Cui
	Nicolas Roux
	Franc-Christophe Baurens
	Xue-Jun Ge
	Trude Schwarzacher
	JS (Pat) Heslop-Harrison
	Qing Liu
**Order of Authors Secondary Information:**	
**Response to Reviewers:**	26 January 2020<br>Dear Editor Hongfang Zhang,<br>We thank the editor and reviewers for their assessment of our manuscript "A chromosome-level reference genome of Ensete glaucum gives insight into diversity, chromosomal and repetitive sequence evolution in the Musaceae" (GIGA-D-21-00354) and the opportunity to prepare a revised manuscript for publication in GigaScience.<br>We are grateful for the extensive comments which were very valuable for improving our manuscript. We have now addressed all of them as outlined below, and we have carefully revised the manuscript, figures, tables and supplementary data accordingly.<br>A clean version of the manuscript is submitted.<br>All authors have approved the revised submission and as before declare no conflict of interest.<br><br>One referee query whether the manuscript should be a “research article” and not a “data note”; this would remain obviously your editorial decision.<br><br>We hope that our revision meets the requirement for publication in GigaScience, and are looking forward to hearing from you in due course.<br>With regards<br>Ziwei Wang, Qing Liu, Mathieu Rouard, Trude Schwarzacher and Pat Heslop Harrison (in the name of all authors)<br>wangziwei@scbg.ac.cn, liuqing@scib.ac.cn, m.rouard@cgiar.org, ts32@le.ac.uk, and phh4@le.ac.uk<br><br><br>Wang et al. "A chromosome-level reference genome of Ensete glaucum gives insight into diversity, chromosomal and repetitive sequence evolution in the Musaceae" (GIGA-D-21-00354)<br>Response to reviewers’ reports<br>Reviewers’ comments are copied and our answer follows after each comment.<br><br>Reviewer #1: Comments to the authors<br>Wang et al. generated a chromosome-scale genome sequence assembly of Ensete

Powered by Editorial Manager® and ProduXion Manager® from Aries Systems Corporation

glaucum based on ONT long reads. This is a valuable resource for comparison against various Musaceae species. This assembly will certainly help to identify genes underlying agronomic traits in Musaceae. Important data sets are already well integrated into the banana genome hub and available to the community. The authors harnessed this highly contiguous assembly for analyses of synteny against Musa acuminata and for the investigation of repeats/TEs. Overall, the quality of this work is high and the manuscript is well written.
Answer: We thank the reviewer for the positive comments.
I am not sure why this submission is classified as a data note, because it could also pass as a research article.
Answer: Our article was originally submitted as a Research Article, but following Editor's request, we had changed it to a Data Note. We agree with the reviewer as we analysed extensively the assembly and propose some interpretation with regards to the evolution of Musaceae.

I noticed a few issues and provided some specific comments that might be helpful to further improve the quality of this work:
Answer: We thank the referee for the detailed and helpful comments.

1) There are many numbers in the abstract. I would recommend to reduce this to the most important ones. For example, the BUSCO results could be removed.
Answer: We agree with this comment and have deleted the BUSCO value, and length and proportion of centromere satellites in the abstract.

2) There is only one short paragraph about existing genome sequences. I would recommend to extend this and to mention the banana genome hub as the central community resource.
Answer: As suggested, we have extended this section to include detail about the available Musa genome assemblies and the banana genome hub (see p. 5, lines 85-90).

3) Please indicate if the coverage estimations are based on the haploid or diploid genome size (Table 1).
Answer: Genome size is based on haploid measurement, as is the convention but should have been stated. Added now to the Table legend.

4) Please provide additional details about the BUSCO results (C, S, D, F, M) in line114 and/or in Table 2.
Answer: Additional BUSCO details have been added to the text summarizing the data in Table S3. See Page 7; lines 125–127.

5) I find the sentence in line 120/121 confusing when reading for the first time. This suggests to me that more sequence was anchored than present in the initial assembly. The sentence is correct, but it might be better to present the total assembly size first and to describe the anchored proportion in a separate sentence.
Answer: We agree and have changed the sentence to read (Page 7; lines 129-131): 'The contig-level assembly size is 495,175,598 bp, and 97.2% of these contigs are anchored to 9 pseudo-chromosomes after Hi-C scaffolding, resulting in a 481,507,213 bp final genome assembly.'

6) It would be helpful to clearly distinguish between the genome (DNA) and the genome sequence (the assembly). That would make it easier to understand the discussion of differences between both (e.g. collapsed repeats).
Answer: We agree. We have made clear where we mean "assembly" and where we mean genome throughout the manuscript.

7) Genome size estimation is always tricky. I would recommend to run several tools and to provide the estimated range (findGSE, gce, MGSE, GenomeScope, ...). It is also important to run the k-mer-based approaches with different k-mer sizes. Apparently, GenomeScope was used for the heterozygosity analysis, but not for the genome size estimation. That is surprising.
Answer: Thank you for this comment. We fully agree with the reviewer that genome size estimation 'is always tricky' and we faced this situation here. There is discussion of genome size measurements "Comparison of Musa acuminata assemblies" in Belser

et al. 2021 with respect to Musa acuminata. We had used several size estimation tools and approaches (with different k-mer sizes) as are conventionally used, and our estimations, and the difference from the assembly and between methods are within the range normally found; our results reveal nothing unusual nor noteworthy. We believe it is best to show results from a similar approach to those published for assemblies of other species, rather than giving extensive comparisons of different methods, given that no one is clearly better than any other. The ancestral genome duplication (evident in the central circle of Fig. 1) influences most methods and means that small changes in parameters change estimates. GenomeScope had been one of several methods of genome size estimation considered.

Following the referee's comment, we have once more generated 17-25mer as input data (see below). We find that findGSE results are quite stable around 588-590 Mb, while GenomeScope results are lower and unstable; it seems as the mer get larger, the estimated size continuously gets larger. The web program version of GenomeScope with k=17, gave a value that was an outlier being below the assembled contig size (as shown in Fig. S1, 468,990,370bp). We also tried MGSE, and the coverage estimates based on mean and median (see below) span the genome size estimates using k-mers.

After careful reconsideration, we believe as before that an estimated haploid genome size of 563,295,571bp as we had reported in Table 2 is the most appropriate estimate. We had based this estimate on the 17-mer peak frequency of Illumina DNA sequencing (with the formula k-num/k-depth where k-num is the total number of 17-mers, 30,417,960,841; and k-depth the highest k-mer depth, 54; see Materials, Methods and Validation section) rather than using findGSE or GenomeScope software. It is also consistent with the gene coverage in raw reads analysed by MGSE as suggested by the referee.

The section in the MS has been updated; see page 7, lines 136-142.

Genome size estimation using different software programs

findGSE:

Genome size estimate for jellyfish.all.findGSE.17.kmers.fa.histo: 588760154 bp.
Genome size estimate for jellyfish.all.findGSE.18.kmers.fa.histo: 581881350 bp.
Genome size estimate for jellyfish.all.findGSE.19.kmers.fa.histo: 585421172 bp.
Genome size estimate for jellyfish.all.findGSE.20.kmers.fa.histo: 588031514 bp.
Genome size estimate for jellyfish.all.findGSE.21.kmers.fa.histo: 588939614 bp.
Genome size estimate for jellyfish.all.findGSE.22.kmers.fa.histo: 590416567 bp.
Genome size estimate for jellyfish.all.findGSE.23.kmers.fa.histo: 590204947 bp.
Genome size estimate for jellyfish.all.findGSE.24.kmers.fa.histo: 590395417 bp.
Genome size estimate for jellyfish.all.findGSE.25.kmers.fa.histo: 590573612 bp.

GenomeScope run on local computer:

GenomeScope analyzing jellyfish.all.17.kmers.fa.histo p=2 k=17 outdir=17
Model converged het:0.115 kcov:55.7 err:0.00236 model fit:1.09 len:242980381
GenomeScope analyzing jellyfish.all.18.kmers.fa.histo p=2 k=18 outdir=18
Model converged het:0.00182 kcov:27.6 err:0.0017 model fit:0.984 len:497383705
GenomeScope analyzing jellyfish.all.19.kmers.fa.histo p=2 k=19 outdir=19
Model converged het:0.00184 kcov:27.3 err:0.00173 model fit:0.97 len:502344401
GenomeScope analyzing jellyfish.all.20.kmers.fa.histo p=2 k=20 outdir=20
Model converged het:0.00176 kcov:27 err:0.00172 model fit:0.964 len:506689085
GenomeScope analyzing jellyfish.all.21.kmers.fa.histo p=2 k=21 outdir=21
Model converged het:0.00172 kcov:26.8 err:0.00171 model fit:0.958 len:510593088
GenomeScope analyzing jellyfish.all.22.kmers.fa.histo p=2 k=22 outdir=22
Model converged het:0.00167 kcov:26.5 err:0.00168 model fit:0.952 len:514042691
GenomeScope analyzing jellyfish.all.23.kmers.fa.histo p=2 k=23 outdir=23
Model converged het:0.00158 kcov:26.3 err:0.00166 model fit:0.949 len:517304358
GenomeScope analyzing jellyfish.all.24.kmers.fa.histo p=2 k=24 outdir=24
Model converged het:0.00153 kcov:26.1 err:0.00163 model fit:0.945 len:520395633
GenomeScope analyzing jellyfish.all.25.kmers.fa.histo p=2 k=25 outdir=25
Model converged het:0.00148 kcov:25.8 err:0.0016 model fit:0.94 len:523313733

GenomeScope run on server:

468990370 bp for web-version of GenomeScope k=21 Figure S1
407601233 bp for web-version of GenomeScope k=17 het 0,.156

Powered by Editorial Manager® and ProduXion Manager® from Aries Systems Corporation

MGSE based on BUSCO gene site coverage:
" processing: result/EGL.NGS.sort.bam.cov
average coverage in reference regions (mean):62.9484059496
average coverage in reference regions (median):75.0"

With the mean coverage as 62.95x and the median as 75x, and 36.878GB of Illumina sequence, this represents 585,830,000 bp or 491,797,000 bp genome sizes, consistent with the k-mer estimate but perhaps distorted by the ancient whole genome duplication, heterozygous genes and recent duplications.

8) Statistics about the pseudochromosomes in Table 2 could be removed. For example, it is not necessary to say that the L50 number of 9 chromosomes is 5.
Answer: We have removed the N50 and L50 lines in Table 2. We had previously given them to facilitate numeric comparisons with published values for less complete assemblies.

9) Please explain the difference in BUSCO results between predicted genes and BUSCO run in genome mode. Which genes are missing in the annotation? Table S3 suggests that the automatic BUSCO annotation (genome mode) is superior to the annotation generated in this study (analyzed in transcriptome mode).
Answer: The two BUSCO results were used to evaluate different aspects. The “genome” mode in BUSCO was to assess the genome assembly completeness; it uses assembly Fasta files as input and it de novo searches the BUSCO genes in the assembly. The transcriptome mode here, on the other hand, was to evaluate our gene annotation quality; we used CDS translated from predicted genes as input, so they are relatively independent. The genome mode BUSCO is commonly superior to the annotation transcriptome mode (see e.g. https://doi.org/10.1016/j.cell.2020.09.043; Table 2).

10) Some statements about the CENs and telomeres would be interesting. These could give a good impression of the assembly results. Estimating their copy numbers could help to explain the difference between assembly size and estimated genome size.
Answer: There are lengthy statements about CENs under the heading Tandem (satellite) repeats and centromeric sequences (seeWe did not analyse the assemblies at telomeres; as with the head-to-head junctions (see point 17), we found problems with the ONT technology, now reported by Tan et al. 12 Jan 2022 (Identifying and correcting repeat calling errors in nanopore sequencing of telomeres BioRXiv https://doi.org/10.1101/2022.01.11.475254). So, with also the cell-to-cell variation, full-length telomere assemblies could not be made. There were some short telomere sequences within a few ONT reads, both mapped internally and terminally on the chromosome assemblies, and these sequences are deposited). We have added telomeres, along with the rDNA, into the comment about the difference between assembly lengths see Page 7; lines 131-132.

11) Are there any genetic markers that could be used to check the assembly accuracy?
Answer: There are extensive publications showing various types of DNA-based markers in Musa and Ensete ventricosum, but not E. glaucum. Both the high level of synteny observed and presence of genes in the BUSCO analysis suggests that many SNP-based markers will work, and provides a check of assembly and its accuracy. The synvisio and dotplot analyses in Fig. 8 further prove extensive synteny between Musa acuminata and E. glaucum. The SSR analysis in Fig. 6 and Table S14 indicates the conservation of frequency of SSR motifs (in combination with our previous analysis of SSR in Musa and E. ventricosum, Biswas et al. 2019). There is no genetic map for any Ensete species to check marker or sequence ordering, but the Hi-C contact matrix (Figure S12) gives another independent check of assembly accuracy. Given our high-quality sequence, future work is more likely to use survey sequencing rather than more limited and laborious genome-wide marker surveys.

12) In my opinion, the section "Gene distribution and whole-genome duplication analysis" could be removed. Genes are never equally distributed across a genome and repeats/TEs are usually clustered around the centromeres. Therefore, this part does not add any novel insights. The second paragraph comes to the conclusion that all

Musaceae share the same WGDs. This seems obvious to me. Was there a different expectation?
Answer: We have deleted the heading and merged it with the previous section now called ‘Genome size, heterozygosity and organization’. We agree there are no novelties here, but it is important to cross-check and document known (expected) results and show these are seen, before we contrast features that differ between the species (see also point 18). Ensete is unique (unexpected) with the centromeric repeat and different LTR retrotransposons as emphasised later.

13) Orthogroup identification could be complemented with a synteny analysis. A comparison to Musa acuminata (https://doi.org/10.1038/s42003-021-02559-3) could help to check the accuracy of the orthogroups.
Answer: Thank you for the suggestion. Orthogroup identification using OrthoFinder (and MCL more generally) is a robust method that have been used extensively in literature, including GreenPhylDB by Guignon et al, 2021(shared authors with our paper). So, we trusted the accuracy and direct comparability to published analyses of results for subsequent analyses. Combining MCL based approaches and microsynteny is indeed a promising strategy for accuracy, although to our knowledge popular tools or pipeline doing both is not that easy to apply.

14) The statement "Genes with Ka/Ks > 1 were under positive selection (Supplementary Table S6)." does not fit well to the rest of this paragraph. Given that there are >35k genes, some would show values >1 by chance. Some statistical test would be needed to find out which genes are actually under positive selection. What is the conclusion from the identification of such genes? Any enrichment of particular functions?
Answer: This is a good point, but analysing which genes are there by chance or due to biology is a lengthy analysis. We have added an additional figure (Figure S2A) to show GO enriched terms of positively selective genes. These summarise the extensive enrichment of biological regulatory processes. We have added this fact in the main manuscript and point out the novel features. see page 10, lines203).Further analysis will be required to examine the details and consequences of genes under positive selection and their enrichment, which are not the focus of this manuscript.

15) The statement about the sugar transporters is interesting. This would be a good chance to connect these comparative genomics results with the transcriptome analyses.
Answer: Again, we agree with the comment, but as a genome-wide and structural analysis of the genome, study of individual gene groups and pathways is beyond the scope of this manuscript.

16) Transcription factor families are mentioned, but not discussed. It is not surprising that MYBs are the largest TF gene family. However, it would be interesting to know if there are any striking differences compared to M. acuminata (https://doi.org/10.1371/journal.pone.0239275). Some MYBs like the anthocyanin regulators respond to sugar treatments. Is there a connection to the large number of sugar transporters? Any duplications/deletions compared to M. acuminata? This could be another opportunity to better connect different aspects of this study.
Answer: Transcription family analysis in a comparative context is certainly important and something we work on (e.g. Cenci A, Rouard M. Evolutionary analyses of GRAS transcription factors in angiosperms. Frontiers in Plant Science. 2017 Mar 2;8:273) and will be studied further in more extensive work, but would be beyond the scope of the current paper.
17) It is interesting to read that head-to-head and tail-to-tail repeats appeared collapsed. Previous studies identified that these arrangements of repeats are associated with low local read quality (e.g. https://doi.org/10.1093/nar/gkaa206, https://doi.org/10.1186/s12864-021-07877-8). I would not expect that both strands of the DNA molecules are sequenced. The authors might want to check this and provide additional explanation.
Answer: We agree we “would not expect”, but there is discussion (unpublished, uncitable and not archived) on the ONT/Nanopore user forum about the phenomenon of a read including a substantial reverse-complement fragment. As suggested, we do see a rapid (although limited) change in read quality between the forward- and reverse-complement components of a single read (giving some support to the artefactual

Powered by Editorial Manager® and ProduXion Manager® from Aries Systems Corporation

nature). We feel it is important to indicate that caution is required in analysis but this is not the place for a detailed study of ONT technology and the base-calling software.

18) I am surprised that TEs were the most abundant class of repeats. Could this be caused by treating at all the different TEs as one group? CENs should appear with a much higher copy number than individual TEs or TE families.
Answer: We are not surprised at the high proportion of TEs which is largely as expected from Musa and most other species. The differences in abundance between classes in Musaceae is unexpected. Many assemblies collapse the TEs so they may be hugely under-represented in the assembly but not in the reads; see comparison of Musa acuminata in Belser et al. 2021 with 246 Mbp of the genome (52.6%) as TEs in V4, compared to 152 Mbp in V2. The length (and genome proportion) of CENs, typically several megabases, far lower than the proportion of retroelements in the genome, is typical of many species.

19) The centromeric patterns could be compared to the situation in Arabidopsis thaliana: https://www.science.org/doi/10.1126/science.abi7489.
Answer: We think it is important to analyse, with the contrast in presence or absence of a centromeric tandem repeat (cf. human vs African Green Monkey). We had already carried out the first analysis of the Arabidopsis centromeric repeat (Heslop-Harrison et al 1999, now confirmed; and cited in the present manuscript with respect to the CENP-B box) but we think a theoretical bioinformatic analysis to compare Ensete and Arabidopsis would need to be complemented by CENH3, ChIP and methylation analysis. It will also need to be in a comparative context of our Musaceae and Arabidopsis (including the former Cardaminopsis) studies of centromeric tandem repeats, and include recent findings in wheat, rice and maize. Again we feel that this is beyond the scope of the present paper.

20) Are SSR less frequent around the centromeres and on the NOR chromosome arm or is this just a lack of detection in these regions?
Answer: SSRs are less common around centromeres and the NOR arm; we used both genome sequence analysis (informatics) and chromosomal in situ hybridization (cytology) (Figure 6B, C): they are independent methods showing the same distribution.

21) Why is AG/CT more abundant than other SSRs? This could be compared to other species.
Answer: We have recently discussed this issue, including comparison with Ensete ventricosum (first pass Illumina assembly only), Musa and many other species (Biswas et al. 2019 - their supplementary figure S2). The extension to Ensete glaucum is made in Fig. 6 and Table S14 and we have now extended the discussion. Page 18; Lines 374-378.

22) References for the length of 45S rDNA length in other species are missing.
Answer: We added three comparator references. However, neither number of copies nor full-length assemblies have been made in many other species with genome assemblies using a directly comparable and accurate unselected read-mapping approach. Analysis of genome assemblies, and particularly programs such as RepeatMasker, give wrong results. Hence addition of comparators using rigorous methods requires extensive (although straightforward) analysis of large amounts of sequence data from other species, beyond the scope of our Musaceae work.

23) How many 45S rDNA copies can be inferred from the ONT reads. The coverage is way higher thus this estimation should be more reliable.
Answer: The coverage of Illumina reads is more than adequate to give an accurate estimate and does not change significantly whether 10% or 100% of the data are used. The challenge with ONT reads determining the number of copies in each read, but the result is consistent with Illumina read analysis.

24) NOR chromosome arm is depleted of protein encoding genes, but there should be plenty of rRNA genes. Please specify this in the sentence.
Answer: Phrasing is corrected to say protein-coding genes are depleted in the NOR arm as figure 7D shows. The tandemly repeated rRNA genes are restricted to a short chromosomal region of this arm (about 3-4Mb of 24Mb, although somewhat collapsed

	in the assembly) and it is notable that the remainder (non rDNA part) of the arm is also depleted of genes.<br>The rDNA monomer is about 10 kb in length and includes transcribed and non-transcribed spacers. rDNA units are interspersed by other repeats (tandem repeats and retroelements) and hence leave little space for other genes. From Figure 2 (circos plots), we also see that the remaining arm is very rich in repeats corresponding to the low gene density; and the synteny analysis of Fig 8A shows the same low gene density for the Musa acuminata NOR arm (compare eg06 and ma10). We can speculate why this is, but we feel that stating the fact is all we can do at this point.<br><br>25) The synteny section is lengthy. The statements in context of previous studies are good, but removing some purely descriptive parts might make it more interesting. The corresponding figures show everything and could stand on their own.<br>Answer: Chromosome structural evolution is the most important, novel, and rigorous analysis which the whole-chromosome assembly allows us, for the first time, to carry out. There are very few equivalents for any plant or animal, and it is therefore emphasized. We feel the entire narrative (results and discussion) is needed for the benefit of the reader.<br>26) What is the value of genotyping-by-sequencing if not combined with GWAS?<br>Answer: Genotyping-by-sequencing has been useful to conduct intraspecific diversity analyses and was also proven as an efficient way to study chromosome structure of banana cultivars as done in Cenci et al. (shared authors with our paper) in https://doi.org/10.1093/aob/mcaa032<br><br>27) Which ONT flow cell type? Which Guppy version?<br>Answer: The flow cell version was R9.4.1 and the guppy version was 2.0.8. We have added this information to manuscript. (see page26, line 568).<br>28) It does not become clear how the Hi-C library was prepared (line 562). What is the improvement? Please explain this here.<br>Answer: We added the detail. (see page 26; lines 576-587).<br><br>29) Please add the detailed parameters of the assembly and polishing.<br>Answer: More details are added in the manuscript in the relevant M&M sections about the assembly. (see pages 27 and 28)<br><br>30) BWA reference is missing. Why was BWA not used for the mapping of the Hi-C reads?<br>Answer: Thank you for noticing; we have added the reference. We used bowtie2 for mapping the Hi-C reads rather than BWA because bowtie2 is the default mapper of HiC-pro.<br><br>31) The statement in line 592/593 suggests that Hi-C was used for validation. However, it was also used for correction in the previous step. Anyways, this result should be moved from the method to the result section.<br>Answer: As stated, the Hi-C and ONT data were used for the primary assembly; we do not state Hi-C was used for 'validation' as that would be a circular argument (in contrast, other published assemblies use Hi-C after assembly for validation and correction). However, the Hi-C contact map is shown in the Materials and Methods as its generation was a key point of the Methods, and it can be usefully compared with other publications.<br>32) Trinity assembly and PASA steps lack details.<br>Answer: More details are added in the manuscript. (see page 28; lines 625-628)<br><br>33) Parameters of STAR mapping and gene prediction steps are missing.<br>Answer: we have now added the information. Software programs where we used specific parameters, we have added these; the rest were used with default settings.<br><br>34) There is some discrepancy concerning the Musa acuminata genome assembly versions. It seems that v2 is used in some cases and v4 in others. Please check this.<br>Answer: All the analyses were performed initially with the released version 2 of Musa acuminata 'DH Pahang'. Late in the preparation of the manuscript the version 4 was published (involving common co-authors), and we felt that this new resource could be more appropriate for the analyses of chromosome structure as the location of centromeric regions was improved. Therefore, we used V4 for these analysis

Powered by Editorial Manager® and ProduXion Manager® from Aries Systems Corporation

(represented in Figure 8) and made it clear in the manuscript in the material and methods/Synteny section (see page 32; lines 708-711) . For the other analyses, each time the version 2 is appropriately indicated.

35) Please make the customized script available via github (line 732) if this is different from the one mentioned in line 737.
Answer: The script was available on github at https://github.com/wangziwei08/LTR-insertion-time-estimation which gives more details. There was an issue with the link in reference 114, which is fixed now.

36) Are the TE results consistent if a different 2Gb subsets of the illumina data are analyzed?
Answer: Good question, but YES, results are consistent. Actually, we used several subsets, and smaller sets with this species and others, and the results do not change within one set of reads once more than c. 1Gb of data are used (also evident from the RepeatExplorer papers of Neuman, Macas et al.).

37) How were the centromere positions determined? I think that I have missed that in the method section. It must be connected to the CEN repeats, but the precise approach could be explained in more detail.
Answer: The centromere region is unequivocally identified by the presence of the repeat Egcen that localises to all chromosomes by FISH at the cytological primary constrictions that are seen as gaps by DAPI staining; this has been described in the result section and Figure 5D-F legend.
We used BLAST of the Egcen consensus to the assemblies, noting the array positions and represented results as bars on graphs for each chromosome in Figure 5B. Single or double arrays correspond to the chromosomal FISH picture (Figure 5D-F). For calculating an inferred centromere mid position (Table S13) we used the start and end of the Egcen arrays and calculated the midpoint for each array with the mean if there are two arrays assuming one is on the right and one on the left of the centromere region. In Figure 8A, a large bar represents the centromere region and in Figure 8B a gap is drawn to indicate that the centromere is not at one particular nucleotide.
This is now explained in the Figure legends to Fig 5 and Table S13 in more detail.

38) The read data sets are not released thus I cannot check if all raw data sets were included. It would be particularly important to have the FAST5 files of the ONT data to study base modifications in the future.
Answer: We agree strongly about having access to FAST5 files (and indeed have asked authors of other papers for these data but most have deleted them). The possibility of upload of FAST5 raw data to NCBI is a new capability but currently the function is not working correctly. The files are available on hard disk by mail, and will be uploaded when the function is available. The raw read dataset of ONT, NGS, Hi-C and RNA have released on NCBI, under BioProject: PRJNA736572 (SRR15039764 - SRR15039770).
39) The link to the banana genome hub appears to be broken in the data availability statement. The data sets on the genome hub look fine.
Answer: Thank you for pointing out this issue. There was a typo with the hyperlink that is now fixed to redirect to a dedicated page to download the datasets.
http://banana-genome-hub.southgreen.fr/

40) The terms "core" and "pseudo-core" in Fig. 3 are not frequently used in the literature. These genes seem to have different degrees of dispensability and might be conditionally dispensable (https://pubmed.ncbi.nlm.nih.gov/24548794/; https://doi.org/10.1186/s13007-021-00718-5).
Answer: We agree with the reviewer that this can be a tricky concept. The pseudo-core concept was used to describe a set of genes similar to what is defined as softcore in the Brachypodium pangenome by Gordon et al, 2017 https://doi.org/10.1038/s41467-017-02292-8. The term is intended to highlight the uncertainty with the number of genes and the challenge of assembling and annotating a particular gene model correctly in every sequenced genome. We changed it to softcore in the manuscript wherever it applied.

41) There seems to be some variation in the genome size estimation. I would recommend to present the results of multiple k-mer sizes (e.g. 17-25). The distribution

of the resulting values might help to estimate the true genome size.
Answer: Please see our answer under point 7.

42) The presented sugar transporters are not among the top enriched GO terms (S2). Therefore, I am afraid that this analysis is not very informative. Could it be that the "enriched" GOs are just a "random" set?
Answer: Carbohydrate metabolism is an important feature of Musaceae but the extensive and detailed analysis of the range of genes, including validation of GO term enrichment, is beyond the scope of the present genome assembly work (see also point 14). Fig. S2 is now revised.

43) Why is E. glaucum not presented as S5C? A direct comparison would make more sense.
Answer: E. glaucum result is now added as Fig S5C; it was not presented in Fig S5 before because it is resented in figure 4B, albeit with a different colour code.

44) S10: I would recommend to identify the precise break points. Next, it would be good to validate the accuracy of the assembly by finding individual reads that actually support the situation in E. glaucum. This would help to exclude an assembly artifact as reason for the difference.
Answer: Figure S10 was a contraction of a much more extensive figure where individual ONT reads were mapped to the exact translocation breakpoints. As with Fig. 5B, ONT reads were able to validate the assembly. The Hi-C contact plot of Fig. S12 also independently validates the accuracy of the assembly since discontinuities in the major diagonal would be evident. We have extended Figure S9 to add (D) the M. balbisiana to mb05 to eg05 dotplot showing the single inversion, and S10B to include some ONT reads spanning the breakpoint. The breakpoint of inversion between E. glaucum and M. balbisiana is after bp 4,478,642 (Fig. S10B) and 7,669,296.

45) It might be better to use a three letter abbreviation of the species ("Egl" instead of "Eg") in the gene IDs to avoid ambiguities in future genome sequencing projects.
Answer: We agree with the reviewer's remark that a 3-letter nomenclature for locus tags and chromosome names would have been more appropriate. It is not practical now to change everything as data are already online for users in the browser (banana-genome-hub) Possibly, if there is a new version of the assembly or gene annotation, it might be an opportunity to apply it.

46) The method section states that short DNA fragments below 12kb were removed. S11 suggests that two libraries were sequences: one with depletion of the short fragments and one without it. Please check this. Generally, I would recommend to try a different gDNA extraction protocol and to use SRE instead of BluePippin.
Answer: We thank the reviewer for the suggestion. We aimed to remove DNA fragments below 12kb, using two approaches. Some DNA molecules may get fragmented during sequencing or entering the sequencer. Fig. S11 shows the high proportion of ONT reads >20kbp for assembly, and we had 229x coverage which is enormously high (even if some reads are shorter – allowing for their removal). Musaceae DNA extraction is difficult: numerous publications in the 1990s and early 2000s discuss reasons, providing "improved" or "optimized" protocols. The DNA isolation approaches we used were already optimized and gave sufficient long-molecule DNA for long read sequencing.

47) The north of eg06 looks suspicious in the Hi-C analysis (S12). There is also no substantial synteny with any of the Musa chromosomes (S8). Could this be an indication that there are errors in the assembly?
Answer: We discuss the interesting picture with eg06, which is likely to be entirely caused by the low protein-coding gene density, presence of the NOR/45S rDNA tandem repeats, and other repeats (see also answer to point 24). The lack of protein-coding genes in this region of eg06 means no substantial syntenic relationships are shown - synteny need syntenic genes.

48) Table S1: What is the point in showing that all contigs are larger than 1, 2, and 5kb?
Answer: This was included for easy and direct comparison with tables in previous publications, although, some like L50, are of no meaning with a highly contiguous

Powered by Editorial Manager® and ProduXion Manager® from Aries Systems Corporation

sequence. We have deleted these lines now.

49) 445 bHLHs in M. acuminata is almost twice the number of bHLHs detected in E. glaucum. Some other TF families also show this large difference, but other families show almost equal numbers. It could be interesting to further investigate this. The HB-KNOX value of M. acuminata is missing.
Answer: We agree that it would be interesting to analyse the differences in the bHLH family (including additional manual curation), and this will be studied in future more extensive work. See also point 16 regarding transcription factors.

Minor comments:

line 70/71: Some countries are named multiple times. Please change this.
Answer: Done

line 113: chromosomes > pseudochromosomes
Answer: Done

line273/274: Please check this sentence.
Answer: we corrected the sentence to read: "In E. glaucum, both Copia and Gypsy families show relatively constant activity over the last 2.5 My, with further major peaks of insertion activity at 3.5 to 5.5 Mya, (Fig. 4B, C) corresponding to the half-life of LTR-elements [14]"

line 428: Please rephrase "translated proteins" and SynVisio should only be named in the method section.
Answer: agreed and done.
line 436: "protein-coding genomes" ?
Answer: corrected into protein-coding genes.

line 464: "second (right)" ... should be replaced by north/south or q/p nomenclature. This also affects some following sentences.
Answer: With largely sub-metacentric chromosomes (and small differences between species 'reversing' arms), we do not think the designation p/q is appropriate (and N/S also indicates the relative sizes with N conventionally the short p arm). The key figures also show horizontal chromosomes with left and right arms as they were assembled.

line 625: "Musa acuminata" is a species name
Answer: Done

line 639: blast > BLAST
Answer: Done

line 731: of of > of
Answer: Done

line 811: RNA-sequencing > RNA-seq (I have not seen a section about RNA sequencing)
Answer: RNA-seq were generated for the purpose to increase the gene annotation and not for gene expression studies. It is hence mentioned in M&M and not discussed in detail in the result/discussion section.

S10: "E glaucum" > "E. glaucum"
Answer: This mistake in Figure S10 has now been corrected.

Reviewer #2: Comments to the authors

In this study, the authors described the generation of a high-quality reference genome of Ensete glaucum, which is one of the most cold-hardy species in the Musaceae. It is also well known for its drought tolerance. The authors compared the expansion and contraction of gene families and the composition of repeats among related species. The genome assembly, analysis, and annotation are certainly useful for comparative genomic studies as well as future breeding practice. Everything seems to make sense to me. Certainly, the results are descriptive, but this is more than sufficient for a data

Powered by Editorial Manager® and ProduXion Manager® from Aries Systems Corporation

	note.<br><br>Answer: We thank the referee for the very positive comments.
**Additional Information:**	
**Question**	**Response**
Are you submitting this manuscript to a special series or article collection?	No
**Experimental design and statistics**<br><br>Full details of the experimental design and statistical methods used should be given in the Methods section, as detailed in our Minimum Standards Reporting Checklist. Information essential to interpreting the data presented should be made available in the figure legends.<br><br>Have you included all the information requested in your manuscript?	Yes
**Resources**<br><br>A description of all resources used, including antibodies, cell lines, animals and software tools, with enough information to allow them to be uniquely identified, should be included in the Methods section. Authors are strongly encouraged to cite Research Resource Identifiers (RRIDs) for antibodies, model organisms and tools, where possible.<br><br>Have you included the information requested as detailed in our Minimum Standards Reporting Checklist?	Yes
**Availability of data and materials**<br><br>All datasets and code on which the conclusions of the paper rely must be either included in your submission or deposited in publicly available repositories (where available and ethically appropriate), referencing such data using a unique identifier in the references and in	Yes

the "Availability of Data and Materials" section of your manuscript.<br><br>Have you have met the above requirement as detailed in our Minimum Standards Reporting Checklist?	

Powered by Editorial Manager® and ProduXion Manager® from Aries Systems Corporation

Manuscript
Click here to access/download;Manuscript;1b_Ensete_Glaucum_Genome_r

A chromosome-level reference genome of *Ensete glaucum* gives insight into diversity, chromosomal and repetitive sequence evolution in the Musaceae

Ziwei Wang[1,2,3], Mathieu Rouard[4,5], Manosh Kumar Biswas[6], Gaetan Droc[5,7,8], Dongli Cui[1,2,3], Nicolas Roux[4], Franc-Christophe Baurens[5,7,8], Xue-Jun Ge[1,2], Trude Schwarzacher[1,6], Pat (J.S.) Heslop-Harrison[1,6,*] and Qing Liu[1,2,*]

[1]Key Laboratory of Plant Resources Conservation and Sustainable Utilization / Guangdong Provincial Key Laboratory of Applied Botany, South China Botanical Garden, Chinese Academy of Sciences, Guangzhou, 510650, China

[2]Center of Conservation Biology, Core Botanical Gardens, Chinese Academy of Sciences, Guangzhou, China

[3]College of Life Sciences, University of the Chinese Academy of Sciences, Beijing 100049, China

[4]Bioversity International, Parc Scientifique Agropolis II, 34397 Montpellier Cedex 5, France

[5]French Institute of Bioinformatics (IFB) - South Green Bioinformatics Platform, Alliance Bioversity and CIAT, CIRAD, INRAE, IRD, F-34398 Montpellier, France

[6]Department of Genetics and Genome Biology, University of Leicester, Leicester, LE1 7RH, UK

[7]CIRAD, UMR AGAP Institut, F-34398 Montpellier, France

[8]UMR AGAP Institut, Univ Montpellier, CIRAD, INRAE, Institut Agro, F-34398 Montpellier, France

* Correspondence: liuqing@scib.ac.cn (QL); phh4@le.ac.uk (JSHH)

Running title: A chromosome scale reference genome of *Ensete glaucum*

Ziwei Wang [0000-0002-5590-2437];

Mathieu Rouard [0000-0003-0284-1885];
Manosh Kumar Biswas [0000-0001-9228-1927];
Gaetan Droc [0000-0003-1849-1269];
Dongli Cui [0000-0003-0254-1364];
Nicolas Roux [0000-0002-8309-3120];
Franc-Christophe Baurens [0000-0002-5219-8771];
Xue-Jun Ge [0000-0002-5008-9475];
Trude Schwarzacher [0000-0001-8310-5489];
JS (Pat) Heslop-Harrison [0000-0002-3105-2167];
Qing Liu [0000-0003-2712-6028].

Abstract

Background: *Ensete glaucum* (2*n* = 2*x* = 18) is a giant herbaceous monocotyledonous plant in the small Musaceae family along with banana (*Musa*). A high-quality reference genome sequence assembly of *E. glaucum* offers a vital genomic resource for functional and evolutionary studies of *Ensete*, the Musaceae, and more widely in the Zingiberales.

Findings: Using a combination of Illumina and Oxford Nanopore Technologies (ONT) sequencing, genome-wide chromosome conformation capture (Hi-C), and RNA survey sequence, supported by bioinformatic analysis of raw reads and molecular cytogenetic fluorescent *in situ* hybridization, we report a high-quality 481.5Mb genome assembly with 9 pseudo-chromosomes and 36,836 genes. A total of 55% of the genome is composed of repetitive sequences with predominantly LTR-retroelements (37%) and DNA transposons (7%). The 5S and 45S rDNA were each present at one locus, on chromosomes 5 and 6 respectively. The 5S rDNA had an exceptionally long monomer length of 1,056 bp, more than twice that of the monomers at multiple loci in *Musa*. A tandemly repeated satellite (1.1% of

the genome, with no similar sequence in *Musa*) was present around all nine centromeres, together with a few copies of a LINE retroelement found at higher frequency at *Musa* centromeres. The assembly, including centromeric positions, enabled us to characterize in detail the chromosomal rearrangements occurring between *E. glaucum* and the x = 11 species of *Musa*. Only one *E. glaucum* chromosome has the same gene content as *M. acuminata* although rearranged. Three *E. glaucum* chromosomes represent part of only one *M. acuminata* chromosome, while the remaining chromosomes involve multiple, complex but clearly defined evolutionary rearrangements in the change between x = 9 and x = 11.

Conclusions: The advance towards a Musaceae pangenome including *E. glaucum,* tolerant of extreme environments, makes a complete set of gene alleles, copy number variation (CNV), and a reference for structural variation available for crop breeding and understanding environmental responses**.** The chromosome-scale genome assembly show the nature of chromosomal fusion and translocation events during speciation, and features of rapid repetitive DNA change in terms of copy number, sequence and genomic location, critical to understanding its role in diversity and s evolution.

Keywords: Centromeres; Chromosome scale assembly; *Ensete glaucum*; Musaceae evolution; Nanopore; Pangenome; Repetitive DNA; Retrotransposons; Synteny; Translocations

Background

The genus *Ensete* Bruce ex Horaninow (Musaceae) includes 10 species of giant, herbaceous monocotyledonous plants, native to tropical Africa and Asia [1]. Among them, the African species *E. ventricosum* (Welw.) Cheesman (enset) is an important food crop for more than 20 million people in Ethiopia [2]. Its sister genus *Musa*, grown throughout the tropics for food and fibre, includes diploid species, triploids and hybrids of *M. acuminata* and *M. balbisiana*, with banana cultivars. Sisters to the grasses (Poales) and palms (Arecales) in monocots, both *Ensete* and *Musa*, along with a third genus *Musella*, belong to Musaceae in the order Zingiberales (gingers and bananas) [3]. Following rapid diversification of the Zingiberales at the Cretaceous/ Tertiary boundary (>65 Mya) the crown node age of the Musaceae family is soon after, with the *Musa* genus diverging from *Ensete* and *Musella* about 40 Mya [4,5].

Ensete glaucum (Roxb.) Cheesman (NCBI: txid482298), like other species in *Ensete*, is monocarpic with a dilated and characteristically glaucous basal pseudo-stem (Fig. 1A-E), with a small number of large seeds (10 mm in diameter) in elongated, banana-like fruits, borne in hands with a terminal flower and is diploid with $2n = 2x = 18$ chromosomes [1,6–10]. *E. glaucum* is widely distributed in Asia (Fig. 1F) and has records from Burma, China, India, Indonesia, Laos, Myanmar, Vietnam, Philippine, Papua New Guinea, Thailand, Solomon Islands [11].

Originating in the tropics and subtropics at lower elevations, most species in Musaceae lack cold acclimation. Cold stress is one of the key limitations in extending banana planting and production to higher altitudes and beyond the tropics [12]. In contrast to other Musaceae species, *Ensete glaucum* can be found above 1000m in the mountains of Yunnan in China, where the temperature often drops lower than 0°C, with limited rainfall in winter. As one of the most cold-resistant and perhaps the most drought-tolerant species in Musaceae, *E. glaucum*

is a potential gene and germplasm resource for abiotic stress tolerance in banana breeding, likely to be required for the adaptation to a more variable and extreme climate in the future.

Whole genome assemblies (genome sequences) are published for some species of *Musa* with pseudo-chromosome-level data [13-17] using long-molecule sequencing with an N50 of more than 42Mbp for *M. acuminata* [17], *M. balbisiana* [15] and *M. schizocarpa* [13]). The assemblies and annotations are available on the banana genome hub, a community website that brings together genomic data, with genome browsers, extensive search facilities. and comparison features [18]. Draft genome assemblies in *Ensete* species are limited to accessions of *E. ventricosum*, but these are with tens of thousands of contigs with an N50 lengths mostly between 10,000 and 21,000 bp and no pseudo-chromosome assignments [19,20]. Effective analysis, introduction and utilization of genetic resources present in wild species of *Ensete*, based around genome assemblies, are a need for banana improvement and understanding the genome evolution in Musacea. We applied Illumina, Oxford Nanopore Technologies (ONT), and chromosome conformation capture (Hi-C) sequencing to generate a high-quality chromosome-level assembly of the *Ensete glaucum* genome. We aimed to use the chromosome sequence to show the genome structure and gene composition, as well as revealing the repetitive DNA organization. The structural variations of *Ensete glaucum* (x = 9) were studied in a comparative context with *Musa* (x = 11) species, showing the evolutionary history of the family. The study aims to be useful in expanding the genepool available not only to banana and enset breeders, but also for plant conservation of biodiversity in ecologically sensitive or threatened areas, and for fundamental research on chromosome and genome evolution.

Analyses, Results and Discussion

De novo chromosome-scale genome assembly

A *de novo* chromosome level assembly of *Ensete glaucum* was made by combining high-coverage ONT long-read sequencing, Illumina 150bp paired-end sequences, and Hi-C chromosome conformation capture sequence data (Table 1). From the initial assembly (with N50 of 10.256 Mb, Table 2 and Supplementary Table S1), we assembled nine pseudo-molecules, eg01 to eg09 (Fig. 2 and Supplementary Table S2), corresponding to the chromosome number (2*n*=18) and observed chromosome morphology.

Table 1: Statistics of whole-genome sequence assembly and transcriptome analysis of *Ensete glaucum* using Illumina, ONT, and Hi-C. Sizes and coverage are based on the unreplicated haploid genome (1C).

Type	Method	Number of reads	Clean data (Gb)	Read length (bp)	Assembly coverage (×)
Genome	Illumina	245,852,534	36.88	2 × 150	74
	ONT	4,357,035	109	38,885 (N50)	220
	Hi-C	319,793,734	48	2 × 150	
Transcriptome	Illumina (Leaf)	62,410,840	94	2 × 150	
	Illumina (Root)	13,712,542	21	2 × 150	

A Hi-C/ONT-only assembly was constructed first, by using an OLC (overlap layout-consensus)/string graph method with corrected reads. Contigs were refined using Illumina short reads, and after discarding redundant contigs, the final genome assembly was 481Mb long, with 9 pseudo-chromosomes between 42,457,113 and 67,484,389bp long. BUSCO analysis [21] was used to assess the assembly in ‘genome’ mode showing 98.3% complete single and

duplicated Embryophyta core gene sets from the embryophyta_odb10 database (Table 2, Supplementary Table S3a): of the 1614 genes tested for, 1526 are complete and single-copy BUSCOs (S), 61 are complete and duplicated BUSCOs (D), 12 are fragmented BUSCOs (F), 15 are missing BUSCOs (M). Few genes were fragmented or missing. *E. glaucum* chromosome designations were chosen to follow major regions of synteny with *Musa acuminata* chromosomes [16,17].

Genome size, heterozygosity and organization

The contig-level assembly size is 495,175,598 bp, and 97.2% of these contigs are anchored to 9 pseudo-chromosomes after Hi-C scaffolding, resulting in a 481,507,213 bp final chromosome level genome assembly. Some arrays of tandem repeats, including the rDNA (see below) and telomeres, were collapsed and chromosome termini were not fully assembled. Around 55% of the assembled genome was estimated to be repeat sequences (RepeatMasker; Table 2). The genome size was estimated as 563,295,571bp (highest 17-mer peak frequency). Presumably because of sensitivity of parameters to the evolutionary whole genome duplications (Fig. 1, centre) and more recent duplications, slightly higher estimates were made by findGSE software (k=21: 588,939,614 bp; range from k=17 to k=25, 582 Mbp to 591 Mbp), and lower estimates by GenomeScope (468,990,370 bp for k=21; or 407,601,233 bp for k=17, Fig. S1). MGSE [22] gave a mean coverage of reference regions of 62.948 and median coverage of 75.00, corresponding to genome size estimates of 587,786,744bp and 493,333,333bp. The total genome size of *E. glaucum* (x = 9) is similar to that of the x = 11 *Musa* species (see [17]) using sequencing methods, and to estimates of both genera by flow cytometry [23].

Table 2: Statistics of *Ensete glaucum* genome assembly and annotation. RepeatMasker did not identify satellite sequences. 5S and 45S rDNA and the centromeric sequence Egcen were identified manually in assemblies and the abundance measured in raw read data; microsatellite abundance was calculated from the assemblies. (See Supplementary Tables S1, S2, S3, S10, S12, and S14).

**Genome assembly**	**Value**
k-mer estimation of genome size (17-mer)	563,295,571 bp
Total contig length	495,175,598 bp
% of estimated genome	87.9 %
Anchored into chromosomes	481,507,213 bp
GC content	38.21 %
Contigs	
Number	124
N50 length	10,255,891 bp
Longest	31,226,749 bp
Pseudo-chromosomes	
Number	9
Shortest	42,457,113 bp
Complete BUSCOs (C) of genome	98.3 %
**RepeatMasker repetitive DNA**	
Transposable elements	
LTR retroelements	37.20 %
Copia	17.64 %
Gypsy	19.25 %
LINEs	0.77 %
Class II DNA transposons	7.18 %
Unclassified dispersed repeats	8.74 %
Simple repeats & low complexity	1.13 %
Total repeats (RepeatMasker)	55.02 %
**Tandem repeat content**	
45S rDNA	1.21 %
5S rDNA	0.08 %
Egcen satellite	1.32 %
Microsatellites (<8bp motif)	0.59%
**Protein-coding genes**	
Number	36836
Average number of exons per gene	4.86
Average exons length per gene	1114 bp
Average intron length	2816 bp
Average length of predicted proteins	371 aa
Complete BUSCOs (C) of predicted genes	94.7%
**Functional annotation**	
NR	31599 (85.78%)
InterPro	30160 (81.88%)
GO	24436 (66.34%)
KO	11192 (30.38%)
Total	31804 (86.34%)

The heterozygosity rate of *E. glaucum* was 0.164% (Supplementary Fig. S1 estimated with k=21 using GenomeScope). Heterozygosity in plants is influenced by mating-systems and pollination [24], life span, habitat fragmentation and cultivation [25]. There is relatively little known about the breeding system and pollination of *Ensete* species (see [26]) although we observed insects (including the hornet *Vespa bicolor*, a widespread pollinator in southern China) visiting flowers (Fig. 1C). Our low level of heterozygosity is within the range found in individual plants in populations of *M. acuminata* ssp. *banksii* (0.02% to 0.34% in 24 individuals, [27]; and 0.13% to 0.23%, [28]), and in other wild monocotyledonous species including two (most likely self-pollinating) diploid oat species (0.07% heterozygosity in *Avena atlantica* and 0.12% *A. eriantha* [29]); it is however, low compared to other species (e.g. walnut, *Juglans nigra* 1.0%, [30]; *Nyssa sinensis* 0.87%, [31]) and in particular, many *Musa* species, some with known hybrid genome composition [28]. The low value seen in species including *E. glaucum* here, is consistent with frequent self-pollination and inbreeding, or a population bottleneck of this monocarpic tropical plant [32].

Genes were unevenly distributed along chromosomes (Fig. 2 circle b), and generally depleted in broad centromeric regions; few genes were found on the short arm of the more acrocentric chromosome eg04 and the Nucleolus Organizing Regions (NOR) bearing chromosome arm of eg06 (see rDNA below). The centromeric, gene poor regions, are rich in repeats (Fig. 2 circle c), transposable elements (*Copia* and *Gypsy* LTR retroelements, Fig. 2 circles d, e, and, less markedly, DNA transposons, Fig. 2 circle f), as observed in many species (including *Musa* [14,15]).

The *K*s (the synonymous rates of substitution) between genes in paired collinearity gene groups were calculated between *E. glaucum* and *M. acuminata* to see whether they share the same three WGD events [14]. The two genomes have a nearly identical *K*s density distribution (Fig. 3A), both having two peaks at around 0.55 and 0.9. This result indicates that Musaceae

share the same WGD events. The more recent peak at 0.55 most likely represents the α and β duplications, while the peaks at 0.9 may represent the more ancient γ duplication event [14]. Fig. 2 (center) links the genomic locations of paralogous gene clusters: most chromosome regions show shared relationships with three other chromosome regions, reflecting the α and β whole genome duplications, as shown by D'Hont et al. [14] (their Supplementary Figure 12).

Gene identification

Genes and gene ontology

In total, 36,836 genes were predicted (BUSCO score: C:94.7%, Supplementary Table S3B) with 31,804 (86.34%) functionally annotated with protein domain signatures and 24,436 (66.34%) associated with GO terms (Table 2; Supplementary Table S4). *E. glaucum* has a similar gene space (Supplementary Table S5) to the sequenced Musa species *M. acuminata* (35,264), *M. balbisiana* (35,148), *M. itinerans* (32,456) and *M. schizocarpa* (32,809). In the Musaceae (i.e., *M. acuminata*, *M. balbisiana*, *M. schizocarpa*, *E. ventricosum* and *E. glaucum*), we identified a total of 29,639 orthogroups including 173,025 (88.1%) assigned genes and 23,355 (11.9%) unassigned genes (Fig. 3B and Supplementary Table S5). Between all species, the analysis showed 48% (n=14,523) of assigned genes were shared (core or softcore genes; rising to 66%, n=19,583 if genes missing in only one species are discounted as possible annotation artefacts). The analyses highlighted 5% (1471) of orthogroups that are *Ensete* genus specific and not found in *Musa*. A total of 162 orthogroups were found only in *E. glaucum* (Fig. 3B; lower than the value for *E. ventricosum* but the latter is a draft genome status without RNA support and with fragmented contigs with likelihood of a large number of redundant predicted genes). The predicted genes of *Ensete glaucum* were compared to their orthologous genes in *M. acuminata* and Ka/Ks values between orthologous pairs were calculated. Genes with Ka/Ks > 1 were under positive selection (Supplementary Table S6). and GO enrichment were used to

summarize the gene functions (Supplementary Fig. S2A) showing many regulatory biological processes have been positively selected.

Gene family expansion and contraction

Using *Musa* species and two other monocotyledonous species (in the same clade of the Commelinids), *Phoenix dactylifera* (Arecaceae) and *Oryza* sativ*a* (Poaceae), we explored gene family expansion and contractions in *E. glaucum* (Fig. 3C and Supplementary Table S7). Among 12,384 gene families shared by the MRCA (Most Recent Common Ancestor) of these monocotyledons, there were large numbers of gene families expanding (1498 to 2184) or contracting (817 to 3444) between the genomes of Musaceae, *Phoenix* and *Oryza* (Fig. 3C), presumably reflecting substantial differences in plant form between them. Similar, although slightly lower, figures were reported between, for example, dicotyledons as diverse as *Arabidopsis* (Brassicaceae), *Solanum* (Solanaceae) and *Cuscuta* (Convolvulaceae) [33]. Notably, though, our results show the largest expansion of gene families in the Musaceae (2184), likely reflecting the whole genome duplication events not shared with the Poaceae or Arecaceae (see also [34] in pineapple), and we find additional expansion in *E. glaucum*. Large gene family losses were noted in *Oryza sativa*, *Phoenix dactylifera* and *Musa balbisiana* (Supplementary Table S7).

Overall, *E. glaucum* showed enrichment of several GO biological processes (Supplementary Fig. S2B, Supplementary Table S8) compared to *Musa*. Among them, "monosaccharide transmembrane transporter" (equal top hit), "carbohydrate transmembrane transport" and "carbohydrate transport"; and among molecular functions, "monosaccharide transmembrane transporter activity", "sugar transmembrane transporter activity", and "carbohydrate transmembrane transporter activity" were all included in the top 20 enrichments. The genus *Ensete* is notable for its accumulation of starch in the pseudo-stem and leaf bases

with *E. ventricosum* cultivated as a staple starchy food in East Africa [2], and perhaps this is reflected in the enrichment of certain carbohydrate transport GO terms.

Transcription Factors (TFs)

In total, 2,637 putative TF genes were identified in the *E. glaucum* assembly, representing 7% of all genes (Supplementary Table S9), which were classified by their signature DNA Binding Domain (DBD) into 58 TF families (Fig. 3D). Similar to *M. acuminata*, the MYB (myeloblastosis) superfamily of transcription factors (including 260 MYB TFs plus 109 MYB-related) was the largest family, with between 140 and 210 copies of each of the bHLH, AP2/ERF, NAC, C2H2, WRKY and bZIP families. The identification and classification of the TFs here provides a framework to explore regulatory networks in plants [35] with their target genes, and to identify specific factors involved in important responses. Cenci et al. [36] analyzed transcription factors involved in the regulation of tissue development and responses to biotic and abiotic stresses and, particularly, the NAC plant-specific gene family, while Xiao et al [37] discuss the importance of a HLH factor involved in starch degradation during fruit ripening. *Ensete* and *Musa* differ in these characteristics so it will be interesting to analyze differences in transcription factors responsible.

Repetitive DNA analysis

Repeat identification

A range of different programs were applied for repeat analysis, and, as has been considered previously [38], there were differences in the repeats identified between approaches, and small changes in parameters and reference sequences give substantial changes. Repeated elements in the genome assembly were identified by RepeatMasker (Table 2 and Supplementary Table S10) and amounted to 55% of the genome assembly, the same range as other plant species with similar DNA amount and, particularly, the genus *Musa* [14,15]. For assembly-free

identification of repeats, we used RepeatExplorer [39] to generate graph-based clusters of similar sequence fragments: Illumina sequence reads are available from six Musaceae species, allowing assembly-free comparisons (Supplementary Table S11 and Supplementary Fig. S3); while there was a little more variation in proportion of reads in the most abundant clusters, all had between 33% and 46% in the top clusters (>0.01% genomic abundance, as defined in [37]). Transposable elements including LTR and non-LTR retroelements, and class II DNA transposons, were found (Fig 4 and Supplementary Table S11). Microsatellites and other repeats were further characterized by mining and dotplot analysis as well as fluorescent *in situ* hybridization to chromosomes (Supplementary Tables S12-S14 and Figs 5-7; see below). The organization of repetitive regions in the assembly was sometimes verified by mapping individual ONT long reads to assembled repeat regions (eg. Fig. 5A), and organization was generally confirmed, except for some long tandem arrays which seem to be collapsed in the assembly due to high homology between repeat units. A few ONT reads were found which included reversals of tandem arrays (head-to-head or tail-to-tail junctions), potentially artefacts from both strands of the DNA molecule passing sequentially through one pore, and these junctions need further investigation.

Figure 4A compares the abundance and species-distributions of major repeat classes in the Musaceae using the comparative genome analysis function of RepeatExplorer. All species shared many transposons and rDNA sequences (Fig 4A, central region). However, genus-specific retroelement variants were identified in *Musa* (Fig. 4A, left) and *Ensete*-with-*Musella* (Fig. 4A, right), showing the separation of the two phylogenetic branches, supported by extensive divergence of the repetitive sequence sub-families, and evolution in copy number. Notably, satellite sequences (Fig. 4A center-right) were much more abundant and some sequences (see centromere sequence below) were present exclusively in *Ensete*.

Transposable Elements

The most abundant class of repetitive elements were transposable elements, in particular LTR retroelements. The distributions of *Copia* and *Gypsy* LTR retroelements along assembled pseudo-chromosomes (Fig. 2 circles d and e) show greater abundance in proximal chromosome regions. Approximately equal numbers of *Copia* and *Gypsy* elements (18 and 19% of the genome assembly respectively, Table 2) were found. This result contrasts with *M. acuminata,* where *Copia* elements were considerably more frequent (29%) compared to *Gypsy* elements (11%;[14]; Supplementary Table S11). The relative change in proportions of the two element families, while the overall abundance remains the same, has implications for genome evolution and the expansion or contraction of retrotransposon families which can be explored in detail using the high-quality genome sequences where the elements are neither truncated nor collapsed.

Analysis of RT domains identified subfamilies of LTR retroelements, with the families showing different abundances in *E. glaucum* and *M. acuminata* (Supplementary Fig. S4). Insertion times of LTR retroelement subfamilies (Figs 4B, C and Supplementary Fig. S5) were calculated based on LTR divergence for *E. glaucum* and recalculated for *Musa* to allow for identical software settings (see Material and Methods). In *E. glaucum,* both *Copia* and *Gypsy* families show relatively constant activity over the last 2.5 My, with the major a peak of insertion activity 3.5 to 5.5 Mya, (Fig. 4B, C) corresponding to the half-life of LTR-elements [14]. The dynamic amplification of these elements is emphasized by individual sub-families having bursts of amplification (Fig. 4B for *E. glaucum* and Supplementary Fig. S5 for *Musa*), with rounds of expansion of different elements. As shown by Wang et al. [15], *M. balbisiana* has the most extensive LTR activity in the last 500,000 years, and *M. acuminata* activity peaks around 1.5 Mya (Fig. 4C), in both cases with greater activity of *Copia* elements (Supplementary Fig. S5), contrasting with *E. glaucum* with equal activity of both *Gypsy* and *Copia* elements

leading to a higher proportion of *Gypsy* elements within the genome of *E. glaucum* compared to *Musa* (see above, and Supplementary Tables S10 and S11). This is also evidenced by the larger number of *Musa* specific clusters identified as *Copia* Angela or Sire elements while *Ensete* with *Musella* specific LTRs include more *Gypsy* Reina and Retand elements (Fig. 4A). Wu et al. [40] discuss the rounds of amplification in *M. itinerans* with an amplification burst after separation from *M. acuminata* about 5.8 Mya suggesting high turnover of the elements. The results suggest a burst of retroelement amplification (the older ones), sometime after the split of *Musa* and *Ensete,* and again more recently, perhaps after *E. glaucum* split from other *Ensete* species.

Tandem (satellite) repeats and centromeric sequences

The repeat analysis revealed the presence of an abundant tandemly repeated sequence with a monomer length of c. 134bp (Fig. 5A). The sequence, named Egcen (*Ensete glaucum* centromere), represents about 1.3% of the *E. glaucum* genome (45,000 copies) (Table 2, Supplementary Table S12; GenBank: OL310717), and forms arrays that are at places interspersed by the LINE element *Nanica* (described in *M. acuminata* [14]) and other sequences (Fig. 5B, see below). One or two major arrays of Egcen repeats were found in the assemblies of all nine chromosomes (Fig. 5C). *In situ* hybridization of Egcen showed it was located around the primary, centromeric, constrictions as seen by DAPI staining (Fig 5D, see also Figs. 7A and C). The hybridization pattern of the FISH signal on all chromosomes showed variable strength and several sites grouped closely together, corresponding to the pattern seen in the assembly. The location of the Egcen arrays was therefore used to infer the centromere mid-point position in the *E. glaucum* chromosome assemblies (Fig 2 outer circle, Fig. 8A, Supplementary Table S13).

Egcen was also detected at the centromeres of all *E. ventricosum* and *Musella lasiocarpa* chromosomes (Fig. 5E, F), showing similar distribution patterns with stronger and weaker

signals as in *E. glaucum* (Fig. 5D), but it was not seen on *Musa* chromosomes by *in situ* hybridization (example of *M. balbisiana*, Supplementary Fig. S6) nor found in analysis of assemblies of *M. acuminata, M. balbisiana* or *M. schizocarpa* (Supplementary Fig. S7A). The comparative RepeatExplorer clustering shows multiple satellite sequences found only in the *Ensete* and *Musella* genomes that are not present in the three *Musa* species tested (Fig. 4A) and supports Egcen being part of the tandem repeat birth and amplification that has occurred in *Ensete* and *Musella* after the split from *Musa*, and contrasts with the younger insertion times found for *Musa* retroelements (Supplementary Table S12 and Fig. 4C).

Tandem repeats or satellite DNA sequences are found around the centromeres of many plant (and animal) species [41,42] and may be 'centromeric' or 'pericentromeric'. No equivalent tandem repeats were found in *Musa* [14,17,43] and the *E. glaucum* Egcen is not present either in *Musa* (Supplementary Figs S6 and, S7A). However, the centromeric regions of all *M. acuminata* chromosomes have been shown to include multiple copies of a LINE non-LTR retroelement, *Nanica*, both by *in situ* hybridization and bioinformatic analysis [14,17]. *Nanica*-related sequences were also identified in the *E. glaucum* assembly, but with less abundance than in *Musa* (Supplementary Fig. S7B); about 350 copies were mapped to chromosomes, mostly (but not exclusively) present interspersed within, or adjacent, to Egcen arrays (Fig. 5B, C and Supplementary Fig. S7B).

Assembly across centromeric regions including abundant repeats is difficult and normally the tandem repeat elements are collapsed. The ONT long-molecule sequences allowed detailed examination of parts of the centromere region of chromosomes. A dotplot of an ONT read (coded 9c7e99b5, 96,300bp long) aligned to the assembly of eg04 shows the complex organization of the Egcen array (Fig. 5B): this 100kb region includes a total of six Egcen tandem blocks with between 3 and 126 repeats (a total of 385), five copies of *Nanica* (some rearranged, pink boxes), and three diverse retroelements flanked by LTRs (green boxes).

Further copies of the Egcen tandem repeat occur in larger blocks over the following 450,000bp of the assembly, and no genes were identified in the region.

A characteristic 17bp long sequence, the canonical CENP-B box, is found within a monomer of a tandem repeat at centromeres of many species including human [44] and has been postulated to be necessary for binding of the centromeric CENP-B proteins regulating formation of centromere-specific chromatin. Within the Egcen sequence, there was a CENP-B related motif:

```
Egcen     ggctaaTACGTTGGTAATGGccagt
                | |||||| || ||
CENP-B     TNNNNTTCGTTGGAAACGGGA
                |  |||| |  | |||
WheatCCS1       TCGGTTGCATGCAGGA
                |  ||  | |
ATcon    ctttgtCTTTGTATCTTCTAACAACAA
```

Although, similar CENP-B motifs have been found from wheat and *Brachypodium* (*CCS1*, [45]) to *Arabidopsis* (ATcon, [46]; see also review [47]), the relevance of the CENP-B related box to centromere function remains uncertain, particularly when no similar tandem repeat is present in other species or the related genera such as *Musa* (see above). As is the case in many other species, the relative roles of retroelements, tandem repeats, and interspersed centromeric sequences, leading to recruitment of the centromeric proteins, are uncertain: the exact sequence or sequences that mark the functional centromeres, remain enigmatic. The identification of a centromeric tandem repeat and the assembly across all centromere regions in *E. glaucum* together with data from *Musa* will allow protein binding studies (with Chromatin Immunoprecipitation ChIP analysis) to resolve the functional centromere.

Microsatellites (Simple sequence repeats, SSRs)

Microsatellites were searched using Phobos and an SSR mining pipeline ([48]; perfect SSRs from mono to hexa-nucleotide repeats with 11 to 3 repeat numbers respectively). SSR

abundances, array lengths, and nucleotide base composition (70% were AT-rich) are shown in Fig. 6A and Supplementary Table S14. The overall nature and abundances of microsatellites in *E. glaucum* were generally similar to *M. acuminata*, *M. balbisiana*, *M. itinerans* and *E. ventricosum* (Table S14), and reflect that AT-rich microsatellites and dinucleotides (in particular AG/CT) are more frequent in Musaceae, in contrast to GC-rich satellites and tri-nucleotide being found more often in Poaceae genomes [49].

An average of one SSR was found per 4000bp, with the density lowest around the centromere and higher at the telomeres (Fig. 6B); they are excluded from the 45S NOR chromosome arm of eg06, and their overall distribution is similar to the distribution of protein-coding genes but contrasts with the more proximal distribution of LTR retroelements and DNA transposons (Fig. 2). Individual microsatellite motifs, however, showed characteristic and different distributions. The abundant microsatellites, CT and AAG, were synthesized as labelled oligonucleotides probes and used as probes for FISH on chromosomes. Both the bioinformatic analysis of the assembly (Fig. 6B) and FISH to chromosomes (Fig. 6C, D) showed (AAG/CTT) has a relatively uniform distribution along chromosomes, while (AG/CT) shows depletion in centromeric regions and greater abundance in distal parts of chromosomes that are gene-rich (Fig.2). The constraints on microsatellite spread in the genome are motif-specific, and, if SSR markers were to be used for genetic mapping, those associated with genes (such as AG/CT) would potentially be more useful.

5S and 45S rDNA and rRNA genes

Tandem repeats of the rDNA were predominantly located within extended, complex, loci on chromosomes eg05 (5S rDNA) and eg06 (45S rDNA) (Figs 2, 5C, 7, and 8). The 45S rDNA monomer containing the 18S rRNA gene - ITS1 - 5.8S rRNA gene - ITS2 - 26S rRNA gene - NTS (GenBank: OL310719) was 9984 bp long, typical but slightly longer than other plant species [50, 51, 52]. The NTS region includes in most cases 16 copies of a degenerate 180bp

tandem repeat. Based on occurrence in the unassembled Illumina reads, there were 587 copies of the 45S rDNA monomer (1.21% of the genome, Tables 2 and Supplementary Table S12). Although in the whole genome assembly the rDNA array was collapsed, the strength of the *in situ* hybridization signal using the rDNA sequence from wheat (Figs 7A, B) is consistent with representing 1% of the genome. The long chromosome arm carrying the 45S NOR locus was depleted in protein-coding genes by 10-fold (average of 12.6 genes/Mb compared to 127.6/Mb on the short arm; (Fig. 7D). The single site of 45S rDNA at the NOR (Nucleolar Organizing Region) per chromosome set is similar to *Musa acuminata* and other *Musa* species [53], although not *E. gilletii* (2n = 18), where there are 4 pairs of sites [23].

The 5S rDNA (GenBank: OL310718) comprised the 5S rRNA gene (119bp long, typical for all plants; eg [51]) and intergenic spacer (937bp), representing 0.078% of the genome or approximately 366 copies, with a complete motif length of 1056bp (Supplementary Table S12). The 5S rDNA locus lies in the middle of the short arm of chromosome eg05, in three parts around 34.5M, 37.5M and 45.5M with multiple interruptions. An example of insertion of a 4.7kb LTR retrotransposon-related sequences in the 5S rDNA array of tandem repeats is shown in Fig 7E. In other regions of the *E. glaucum* ONT reads or assembly, the retroelement-related sequences named *Brep,* reported in *Musa* [54], was also found in the 5S rDNA arrays. Garcia et al. [55] show the rather unusual and highly complex structure of 5S rDNA in *M. acuminata* using graph-based clusters of reads with multiple IGS and retroelement components, supporting the complexity reported here in the *E. glaucum* assembly. The multiple hybridization sites evident from the *in situ* hybridization site on one pair of chromosomes (Fig. 7B, C), with several, non-continuous, signals visible in the extended prometaphase chromosomes (Fig. 7C, insets) support the non-continuous nature of the 5S rDNA array.

The 5S rDNA monomer length of 1056bp was exceptionally long in comparison to any other plant species (typically 400-500 bp long). The first 400bp of the intergenic spacer had no

significant BLAST hits in GenBank, while the second part showed only short regions with weak homology largely to chromosome assemblies of *Musa* species in GenBank. There were no motifs characteristic of retroelements in the 937bp intergenic spacer. It is unclear why the monomer length for the 5S rDNA in *E. glaucum* should be twice that typical in other species, including *Musa*, and was to be relatively homogeneous over all copies (Fig. 7E). Furthermore, in contrast to the single locus on eg04 of *E. glaucum*, all species of *Musa* examined so far have multiple 5S rDNA sites (2, 3 or 4 per genome), and *E. gilletii* had 3 pairs of sites [23,53].

Different species in the Triticeae show wide variation in numbers and locations of both 45S and 5S rDNA sites, suggesting multiple and complex evolutionary rearrangements of the chromosome arms [56] even in the absence of chromosomal rearrangements including translocations and inversions. Dubcovsky and Dvorák [57] have considered the 45S rDNA loci as the "nomads of the Triticeae genomes" given their repeated evolutionary changes in position during species radiation without rearrangements of the genes of the linkage groups. The depletion of protein-coding genes in chromosomal regions extending over most of a chromosome arm around the 45S rDNA genes is notable in *Musa* and *Ensete*, so chromosome rearrangements can lead to loci moving, although other recombination, duplication, deletion or translocation events must occur to alter the numbers of both 5S and 45S loci observed.

Synteny and chromosome rearrangements to *Musa*

Structural comparisons of the *E. glaucum* genome assembly (x = 9, chromosomes eg01 to eg09) were performed with the high-quality assembled genomes of *M. acuminata* (x = 11, ma01 to ma11; v4, [17, 58]) based on synteny (Fig. 8A). The comparison was also extended to *M. balbisiana* (mb01 to mb11;[15]; Supplementary Fig. S8). Sequence dotplots (Fig. 8B) and comparative karyotypes (Fig. 8C and Supplementary Table S15) of the *E. glaucum* genome against the *M. acuminata* genome were also analyzed.

Overall, the genome assemblies of *M. acuminata* and *E. glaucum* are very similar in length and gene content (Supplementary Table S5). We observed high identity between segments of the 9 chromosomes of *E. glaucum* and of the 11 chromosomes of the *Musa* (Fig 8A, Supplementary Fig. S8). Broad centromeric regions with few protein-coding genes (Fig. 2) cannot show syntenic domains. The number of collinear genes was 48,956 between *E. glaucum* and *M. acuminata,* and 39,604 between *E. glaucum* and *M. balbisiana* (by comparison, the A and B genome of *Musa* show 42,854 collinear genes). Chromosomes show rearrangements, inversions, expansions or contractions by crossed, converging or spreading lines in the Synvisio plots (Fig. 8A). The dotplot (Fig. 8B; single chromosome comparison in Supplementary Fig. S9) shows that there are some syntenic regions distributed over the same length of chromosomes in both species (diagonal lines showing synteny at 45°, eg08/ma10). In other cases, there is expansion in one genome and not in the other (lines of synteny more vertical, eg04/ma04, or nearer horizontal, eg03/ma04). Many syntenic segments showed curved lines (ma03/eg03), showing relative expansion of one genome at one end of the conserved syntenic block, and expansion of the other genome at the other end.

One complete chromosome, ma05/eg05/mb05, was similar with the same gene content in all three species (Fig. 8A and Supplementary Fig. S8), but it showed multiple internal inversions and expansions/contractions. A nested pair of inversions was evident covering 10.4Mb near the start of the chromosome in *E. glaucum* with respect to *M. acuminata* (8.84Mb region) in dotplots (Supplementary Fig. S9A) and by comparison of locations of orthologous genes (Supplementary Fig. S10). In the context of the *E. glaucum* inversions, we could also examine the ancestral structure of *M. acuminata* and *M. balbisiana* reported by Wang et al. [15]. Notably, a major rearrangement involving an inversion between *M. acuminata* ma05 and *M. balbisiana* mb05 [15] was the same inverted region as found in eg05, with an additional nested inversion of 3.1Mb in eg05 with respect to ma05 (Supplementary Figs S8, S9, S10).

Using positions of orthologous genes at the boundaries of syntenic regions, the inversion structure between chromosomes eg05, ma05 and mb05 was clear. Regardless of the ancestral condition, the result indicates closely similar inversion breakpoints were involved (at the ends of the segment, Supplementary Fig. S10) twice during evolution.

Apart from chromosome 5, three further whole chromosomes of *M. acuminata* are represented largely by a single, whole chromosome regions/arms of *E. glaucum*, with some rearrangements occurring within the chromosomes (Fig. 8): ma01 is mainly the right arm of eg01; and ma02 is mainly the right arm of eg02 (Fig. 8A); ma11 is entirely the left arm of eg09 (see details in the dot blot of Supplementary Fig. S9B). The other arms of these three *E. glaucum* chromosomes (eg01, eg02 and eg09) and the remaining six chromosomes are related to blocks of the remaining seven *Musa* chromosomes. Four *Musa* chromosomes have translocated fusions of segments of two *Ensete* chromosomes. ma09 has the intercalary region of eg07, with an intercalary segment of eg06 inserted within the eg07 region. ma10 includes parts of three *Ensete* chromosomes, while ma04 has four segments from *Ensete* chromosomes (Figs 8A, C). The 45S rDNA on eg06 and ma10 are surrounded by syntenic regions but are both depleted in protein-coding genes (Fig. 2). In contrast, the 5S rDNA sites are not surrounded by other orthologous genes (see above). The non-reciprocal translocation noted by Wang et al. [15] of a terminal segment between ma03 and mb01 lies within a larger syntenic block shared between ma03 and eg03. This suggests that the translocation occurred in the *M. balbisiana* lineage (Supplementary Fig. S8).

In several chromosomes of both *E. glaucum* and *M. acuminata*, the breakpoints occur in the centromeric regions (e.g., in eg02, eg03, eg07, eg08 and eg09; ma01, ma06, and ma10; Fig 8A). While some breakpoints occur at or adjacent to centromeres, the exact relationship of any breakpoint to the centromere and Egcen or *Nanica* sequences is diverse. Notably, eg03 is spanning the centromere of three *Musa* chromosomes, ma03, ma04 and ma08, and in other

cases centromere regions are different despite surrounding synteny (e.g., eg07, ma07 and ma09). Telomeric or sub-telomeric regions are conserved between the two species in 7 of the 18 *E. glaucum* chromosome arms (e.g., eg01/ma01, eg06/ma07, eg08/ma10; the dotplot homology lines end in the corners of the chromosomes, Figs 8B and Supplementary Fig. S9B). In other chromosomes, telomeres in *E. glaucum* are in intercalary regions of *Musa* (see eg03/ma03 with inversion, eg08/ma06 in Fig 8B; and in detail eg08 and ma10 in Supplementary Fig. 9C). The homology of the whole chromosome ma11 and the left arm of eg09 (see above) indicates a fusion/fission event with loss/gain of centromere and telomere function, but they are also predicted for the other rearrangements discussed above.

Song et al. [10] review the data on the basic chromosome number of the Zingiberales, concluding that x = 11 is most reasonable original basic number, with x = 9 as a derived basic number. With chromosome numbers of x = 9, 10 and 11 predominant in Musaceae, this family is particularly suitable to explore the nature and locations of chromosomal fusions and fissions that are predicted to often occur in similar position during karyotype evolution (e.g. in wheat [59]). The availability of high-quality genome assemblies, based on the ONT, Hi-C and in *Musa* BioNano and PacBio technologies, will allow the nature of breakpoints in chromosome fission events to be investigated at the sequence level between the *Musa* x=11 and *Ensete* x=9 species, as well as being able to shed light to centromere and telomere function.

Conclusions

We provide a chromosome-scale assembly of *Ensete glaucum*, a sister genus to *Musa*. This assembly is valuable to infer *Musa* genome evolution, enabling comparison with putative last common ancestors of *M. acuminata* (A genome) and *M. balbisiana* (B genome) at protein and chromosomal levels. Most striking was the multiple rearrangements of chromosome structures between *E. glaucum* and the *Musa* A and B genomes, with only 4 of the 11 *M. acuminata*

chromosomes (and only 3 of the 11 in *M. balbisiana*) showing synteny with only one or part of one *E. glaucum* chromosome. With the new insight into chromosome evolution here, further assemblies (in particular the *Callimusa* section with n=7, 9 and 10) will enable resolution of ascending or descending dysploidy, in Musaceae, its sister clades in the Zingiberales and more widely.

As well as the complex chromosome rearrangements, repetitive sequences differ extensively between the *Musa* and *Ensete* genera. There is a major tandem repeat at the centromeres of only the *Ensete* species, showing lack of conservation of this key structural element of chromosomes, although both genera have multiple copies of the *Nanica* retroelement in centromeric regions. *E. glaucum* has only one 5S rDNA locus, with an unusually long monomer of 1056bp.

The complete sequence provides an accurate reference for the genus for gene identification, marker development, Genotyping-By-Sequencing (GBS), and Genome Wide Association Studies (GWAS) and will accelerate our understanding of the molecular bases of traits such as cold tolerance and starch accumulation and allow identification of relevant genes, contributing to the aim of the Earth BioGenome Project [60] to sequence all eukaryotic species. Although not yet fully understood, the role of chromosomal structural variation and sequence copy number variation (both of genes and repetitive DNA) in genotypic and species diversity is increasingly being recognized, and our high continuity assembly provides a reference for such studies. The work builds towards a complete pangenome of the Musaceae family, defining structural, gene and genetic diversity which can be used for genetic improvement across the Musaceae and more widely.

Material, Methods and Validation

Sample collection and distribution

The individual *Ensete glaucum* plant used for genome sequencing and analysis was collected from Puer city, Yunnan province, China and maintained in the South China Botanical Garden, Guangdong province, China (accession no. 19990288; Fig. 1A-E). The distribution of *Ensete* and *Musa* species were identified in databases of Flora of China, South China Botanical Garden, iNaturalist, GBIF [61] (excluding cultivation sites) and regional distributions maps. Fig. 1F was then made from POWO [62] (overlaid and color-adjusted in Adobe Photoshop CC2018).

DNA extraction and sequencing

Young leaves of *Ensete glaucum* were collected and ground into powder in liquid nitrogen. High molecular weight genomic DNA was extracted using the DNeasy Plant Mini Kit (Qiagen, Hilden, Germany). DNA quality was assessed by agarose gel electrophoresis and NanoDrop 2000c spectrophotometry, followed by Thermo Fisher Scientific Qubit fluorometry.

Illumina sequencing

A genomic DNA library with 400bp fragments was constructed using Truseq Nano DNA HT Sample preparation Kit (Illumina, USA), and 150bp paired ends were sequenced with Illumina Novaseq (Illumina NovaSeq 6000 Sequencing System, RRID:SCR_020150) by Grandomics Biosciences Co., Ltd. (Wuhan, China) (previously known as Nextomics, Wuhan, China). After applying Trimmomatic v0.36 (Trimmomatic, RRID:SCR_011848) [63] to trim adaptors, filtering out low quality reads and further quality control with fastQC v0.11.9 (FastQC, RRID:SCR_014583) [64], 246 million paired reads and 36.88Gb of data resulted (Table 1).

Oxford Nanopore (ONT) sequencing

ONT (Oxford Nanopore, Oxford, UK) sequencing was performed by Grandomics Biosciences Co., Ltd. (Wuhan, China): long fragments longer than 12 kb were selected with Sage Sciences BluePippin (Sage Science BluePippin system, RRID:SCR_020505), and the SQK-LSK109 kit (Oxford Nanopore) was used to build a library that was sequenced using PromethION (PromethION, RRID:SCR_017987), flow cell R9.4.1. The base calling was performed with Guppy v2.0.8 and reads mean_q score_template (Phred) > 7 (base call accuracy >80%) were selected. A total of 129 Gb ONT reads (~ 250X coverage) was generated. fastp v0.19.7 (fastp, RRID:SCR_016962) [65] was used for quality control including adaptor-trimming, filtering reads with too many Ns or mean q score lower than 7 and resulted in remaining clean data of 109 Gb (Table 1). The mean read length was c. 20kb, with the longest >120kb (Supplementary Fig. S11).

Hi-C chromatin interaction data

Genomic DNA was extracted from *E. glaucum* for Hi-C analysis and generation of a contact map to anchor contigs onto chromosomes [66, 67]. Firstly, freshly harvested leaves were cut into 2 cm pieces and vacuum infiltrated in nuclei isolation buffer supplemented with 2% formaldehyde. Crosslinking was stopped by adding glycine and additional vacuum infiltration. Fixed tissue was then ground to a powder before re-suspending in nuclei isolation buffer to obtain a suspension of nuclei. The purified nuclei were digested with 100 units of *Dpn*II and tagged with biotin-14-dCTP. Biotin-14-dCTP from non-ligated DNA ends was removed owing to the exonuclease activity of T4 DNA polymerase. The ligated DNA was sheared into 300−600 bp fragments, and then was blunt-end repaired and A-tailed, followed by purification through biotin-streptavidin-mediated pull down. Finally, the Hi-C libraries were quantified and sequenced using the Illumina HiSeq platform (performed by Grandomix, loc. cit.). Low-quality

sequences (quality scores<20), adaptor sequences, and sequences shorter than 30 bp were filtered out using fastp v0.19.7 [65].

Genome and chromosome assembly

ONT data were corrected by Nextdenovo v2.0-beta.1 [68], with setting “read_cutoff = 3k，seed_cutoff = 25k， blocksize = 2g” and the 109Gb filtered data were assembled by SMARTdenovo (SMARTdenovo, RRID:SCR_017622) [69] with the parameters "wtpre -J 3000，wtzmo -k 21 -z 10 -Z 16 -U -1 -m 0.1 -A 1000，wtclp -d 3 -k 300 -m 0.1 -FT，wtlay -w 300 -s 200 -m 0.1 -r 0.95 -c 1". To polish the assembly, contigs were refined with Racon (Racon, RRID:SCR_017642) [70], BWA v0.7.17 (BWA, RRID:SCR_010910) [71] was used to map the filtered Oxford Nanopore reads to the assembly and NextPolish v1.3.1 [72] with parameters " --consensus -w window -t 4 -m 0.5 -d 30" was used to discard possibly redundant contigs and generate a final assembly; similarity searches were performed with the parameters “identity 0.8 – overlap 0.8”. Finally, BWA v0.7.17 [71] and Pilon v1.21 (Pilon, RRID:SCR_014731) [73] with setting "--changes --vcf --diploid --fix bases --threads 10 --mindepth 10" were used to further correct the assembly using the Illumina Novaseq reads, and two rounds of mapping back to the assembly each time with further correction were undertaken. A 494Mb assembly with 124 contigs was achieved (Table 2 and Supplementary Tables S1, S2).

Read pairs from the Hi-C data were mapped to the draft assembly using bowtie2 v2.3.2 (bowtie2; RRID:SCR_016368) [74] with the settings "-end-to-end, -very-sensitive and -L 30" to select unique mapped paired-end reads. Valid interaction paired-end reads were identified by HiC-Pro v2.8.1 (HiC-Pro, RRID:SCR_017643) [75] and retained for further analysis while invalid read pairs, including dangling-end, self-cycle, re-ligation, and dumped products were discarded. The scaffolds were further clustered, ordered, and oriented onto pseudo-

chromosomes by LACHESIS (LACHESIS, RRID:SCR_017644) [76],with parameters as follows: "CLUSTER_MIN_RE_SITES=100, CLUSTER_MAX_LINK_DENSITY=2.5, CLUSTER NONINFORMATIVE RATIO = 1.4, ORDER MIN N RES IN TRUNK=60, ORDER MIN N RES IN SHREDS=60". Finally, regions with obvious discrete chromatin interaction were detected and their placements and orientations were manually adjusted (Supplementary Fig. S12). Validation of the assembly was performed using Benchmarking Universal Single-Copy Orthologs with v5 (BUSCO, RRID:SCR_015008) [21] to assess the completeness and presence of 1614 genes in the embryophyta_odb10 database in "genome" mode (Supplementary Table S3a).

RNA extraction, sequencing and transcriptome assembly

Total RNA was extracted from fresh leaves and roots of the same individual of *Ensete glaucum* that was used for genomic sequencing using RNeasy Plant Mini Kit (Qiagen China (Shanghai) Co Ltd. China). Illumina libraries were built from 1 μg total RNA of each sample with TruSeq RNA Library Preparation Kit (Illumina, USA) and were then sequenced using Illumina Novaseq platform to generate paired-end reads. A transcriptome assembly was produced using RNAseq data using Trinity (Trinity, RRID:SCR_013048) [77] with parameters: "--genome_guided_bam EGL.star.bam --max_memory 50G --genome_guided_max_intron 10000" and mapped on the genome with PASA (PASA, RRID:SCR_014656) [78] with setting:" --MIN_PERCENT_ALIGNED=80 --MIN_AVG_PER_ID=80".

Genome size estimation

Using the Illumina DNA sequence, genome size was estimated from the 17-mer frequency using Jellyfish v2.0 (Jellyfish, RRID:SCR_005491) [79] with the formula k-num/k-depth

(where k-num is the total number of 17-mers, 30,417,960,841; and k-depth the highest k-mer depth, 54; Table 2). 21-mer data were used in findGSE [80] and Genomescope 2.0 (Genomescope R, RID:SCR_017014) [81] to estimate the genome size and heterozygosity (Supplementary Fig. S1).

MGSE v0.4 [22] were also used to estimate the genomic size based on read-mapping coverage. The NGS reads were mapped to assembly by BWA v0.7.17. The coverage of single copy genes (BUSCO genes) was calculated by MGSE.

Gene annotation

We adopted a combination of *ab initio* gene prediction, homology-based gene prediction and transcriptome-based gene prediction strategy. RepeatMasker v4.0.9 (RepeatMasker, RRID:SCR_012954) with option "-no_is –xsmall" was used to generate a repeat softmasked genome file. RNAseq data from leaf and root tissues were mapped to the masked genome assembly with STAR v2.7 (STAR, RRID:SCR_004463) [82] with option: "--outSAMtype BAM SortedByCoordinate --outSAMstrandField intronMotif --outFilterIntronMotifs RemoveNoncanonical". The RNA alignment was input into BRAKER2 v2.1.5 (BRAKER, RRID:SCR_018964) [83], a combination of GeneMark (GENEMARK, RRID:SCR_011930) [84] and AUGUSTUS (RRID:SCR_008417) [85], to perform *ab initio* gene predictions with the default settings. The gene models from BRAKER2 were inputted into MAKER v2.31.10 (MAKER, RRID:SCR_005309) [86] as model, and the RNA alignment of *E. glaucum* and proteins form *M. acuminata* v2 were used as EST and protein evidence, respectively. We also utilized GeMoMa v2.3 (GeMoMa, RRID:SCR_017646) [87] to perform homology-based gene prediction using *M. acuminata* v2 [16] as reference annotated genome. EvidenceModeler (EvidenceModeler, RRID:SCR_014659) [88] was used to combine de novo and homology-

based predictions and our transcriptome evidence to produce the final structural gene annotation.

To annotate the function of predicted genes, we performed BLASTP (e-value = 1e−10) (BLASTP, RRID:SCR_001010) from the BLAST+ package [89] for each predicted coding sequence against the databases: UniProtKB/Swiss-Prot, UniProtKB/TrEMBL [90] and NR (non-redundant protein database at NCBI). These sequences are then processed to produce a non-identical (often referred to as pseudo non-redundant) prediction. To assign a putative function to a polypeptide we kept only the best hit based on three parameters: (1) Qcov (Query coverage = length high-scoring segment pair (HSP)/length query), (2) Scov (Subject coverage = length HSP/length subject) and (3) identity. Additional functional information was added by scanning sequences with InterProScan v5.46 (InterProScan, RRID:SCR_005829) [91]. Blast2GO v6.0.1 (Blast2GO, RRID:SCR_005828) [92] was used to integrate the results of BLAST and InterProScan, and to link the GO (Gene ontology) terms to genes accordingly (Supplementary Table S4) The functional annotation procedure is given in greater detail at [93].

BUSCO was run in mode “transcriptome” using the embryophyta_odb10 database to assess the gene annotation results and found 1529 (94.7%) complete BUSCOs (Supplementary Table S3b).

Gene family analyses

Orthogroups (OGs) identification in Musaceae

Protein-coding genes from *M. acuminata* [16], *M. balbisiana* v1.1 [15] and *M. schizocarpa v*1 [13] were retrieved from the Banana Genome Hub [18]. Protein-coding genes predicted from *E. ventricosum* was downloaded at NCBI Genome (GCA_00

0818735.3) to allow discrimination of *Ensete* specific OGs *E. glaucum* specific OGs. Combined with *E. glaucum* protein-coding genes, we used OrthoFinder v2.5.2 (RRID:SCR_017118) [94] and Diamond [95] with default parameters (summary in Supplementary Fig. S5). Visualization (Fig. 3B) was produced with UpsetR [96]. Gene ontology (GO) enrichments were calculated using TopGO [97] with Fisher's exact test (Supplementary Fig. S2 and Supplementary Table S8).

Gene family expansion and contraction

To identify gene family expansion and contraction, we expanded previous analyses with OrthoFinder by adding a representative of Musaceae sister clades in Palms (*Phoenix dactylifera*, date palm, [98]) and Poales (*Oryza sativa v7*, rice, [99]; data downloaded from Phytozome [100]) but omitting *E. ventricosum* due to gene redundancy. The longest transcripts were kept if alternative splicing occurred. Divergence time estimation with Approximate Likelihood Calculation used MCMCTREE in PAML v4.9j (PAML, RRID: SCR_014932). Computational Analysis of gene Family Evolution (CAFE v4.2.1, RRID:SCR_018924) [101] was used to model the evolution of gene family sizes and stochastic birth and death processes and summarized in the phylogenetic tree (Fig. 3C).

Transcription factors

Protein coding gene sequences for *E. glaucum* and *M. acuminata* v2 were searched in PlantTFDB v5.0 (PLANTTFDB, RRID:SCR_003362) and iTAK online v1.6 [102]. Predicted transcription factors (TFs) were verified through a Hidden Markov Model (HMM) with PFAM searching tools using the cutoff E-value of 0.01 (Fig. 3D and Supplementary Table S9). Genes were verified by PFAM (Pfam, RRID: SCR_004726), CDD (Conserved Domain Database, RRID:SCR_002077, and SMART (SMART, RRID:SCR_005026) databases.

Whole-genome duplication (WGD)

To identify the whole-genome duplication events, we applied WGDI pipeline (whole-genome duplication identification v0.4.7 [103]). The predicted proteins of *E. glaucum* were blasted against themselves and then a collinearity analysis was conducted. The *K*s (the synonymous rates of substitution) between genes in paired collinearity gene groups were calculated and the *K*s peak was detected. For comparison, the same processes were also applied to *Musa acuminata* v2 [16].

Synteny analyses

Structural comparisons of the *E. glaucum* genome were performed with *M. acuminata* v4 (designated the A genome) and *M. balbisiana* (B genome). The very recent release of *M. acuminata* v4 assembly was preferred in this case as it improved pericentromeric regions and provided telomere-to-telomere gapless chromosomes [17]. Assemblies were aligned with minimap2 (Minimap2, RRID:SCR_018550) [104] and visualized results using D-Genies (D-GENIES, RRID:SCR_018967) v1.2.0 [105]. Protein-coding genes were processed to identify reciprocal best hits (RBH) with BLASTP (e-value 1e-10) followed by MCScanX (e-value 1e-05, max gaps 25) [106] and results imported in SynVisio [58] for syntenic block visualization. Scale bars and coloring of the chromosome bars was adjusted using Adobe Photoshop CC2018.

The karyotype of *E. glaucum* in Fig. 8C, was prepared from lengths of each pseudo-chromosome (Supplementary Table S2) with the estimated centromere position (using the Egcen array midpoints, Supplementary Table S13) to estimate the left (darker colored) and right (lighter colored) chromosome arms. Chromosome lengths and centromere positions for *M. acuminata* were taken from [17] (Fig. 2a); syntenic blocks were calculated using the SynVisio diagram (Fig. 8A).

Repetitive DNA identification and annotation

For repetitive DNA analysis, publicly available programs (see below and [38]) as well as manual searches and sequence comparisons were applied. Geneious v.10.2.6 (Geneious, RRID: SCR_010519) (Biomatters Ltd., Auckland, New Zealand) was used to produce the dotplots of Figs 5A, B, 7E and 8B, and supplementary Figs. S9).

In the assembly, repeated sequences were first searched with REPET v2.5 pipeline [107]. The top 100 repeated sequences were plotted on the four reference *Musa* genome assemblies (i.e. *M. acuminata*, *M. balbisiana*, *M. schizocarpa* and *E. glaucum*) using BlastAndDrawDensity.py script described in [17] and available on the Github repository [108],

Transposable Elements

Transposable elements were annotated by EDTA pipeline [109], which integrates various software to discovers TE including: long terminal repeat (LTR) retrotransposons [110–112], terminal inverted repeat (TIR) transposons [113], short TIR transposons or miniature inverted transposable elements (MITEs) [114], and Helitrons [115]. According to suggestions in [109], we also adopt RepeatModeler2 (RepeatModeler, RRID:SCR_015027) v2.0.1 [116] to find remaining TEs.

We also discover repetitive elements through REPET v2.2 [117] package including TEdenovo and TEannot. The TEdenovo procedure was used on masked assembly to produce a batch of 4229 TE consensus sequences. From these 2800 consensus sequences, only those with full length fragments present in the assembly were kept for further analysis, quantification and annotation with the TEannot procedure. A first annotation was performed using public Repbase (Repbase, RRID:SCR_021169) release 20.05, followed by *Gypsy/Copia* retroelement family identification using Hidden Markov Models (hmmsearch version 3) to search consensus for corresponding retro-transposase PFAM domains PF04195 and PF14244, respectively. The

above results were then combined and CD-HIT v4.1.8 (CD-HIT, RRID:SCR_007105) [118] was used to reduce redundancy. The LTR retrotransposons were sent to TEsorter [119] to classify into lineage level and RT domain amino acid sequences were extracted. Phylogenetic trees of *Copia* and *Gypsy* were inferred by RT domain alignment results (Supplementary Figure S4). The proportions of TEs in the assembly are given in Table 2 (Supplementary Tables S11 and S12) and chromosomal distributions in Fig. 2.

To estimate ages of LTR retrotransposons and the time of insertion (Fig 4B, C and Supplementary Figure S5), complete elements were found by LTRharvest v1.6.1 (LTRharvest, RRID:SCR_018970) [110] and LTR_retriever (LTR_retriever, RRID:SCR_017623) [111] and then classified by TEsorter v1.3. The estimation of time was based on the divergence of the 5' and 3' end LTRs, and these two LTRs of every LTR retrotransposons were extracted into separate files with a custom script. The 5' and 3' LTRs were aligned by MUSCLE v3.8.1551 [120]. The divergence distances under K2P evolutionary model were calculated by R package ape v5.4-1 (ape, RRID:SCR_017343). The average base substitution rate was selected to be 11.3E-8 [121]. The insertion time T was calculated as T = K/(2*r*), with r as the rate of nucleotide substitution and K as the divergence distance between LTR pairs. The script to perform the analysis is on Github [122].

Graph based clustering of reads using RepeatExplorer

A sample of 2Gb of the Illumina HiSeq raw reads were used for assembly-free analysis by RepeatExplorer2 [39]. Graph-based clusters of similar sequence fragments were generated under default parameters. Clusters were assigned to repeat classes and retroelement lineages using the automated Repeat Masker and Domain hits provided by the program (Supplementary Table S11). Comparative analysis with sample Illumina sequence reads from other five Musaceae species, namely *M. acuminata* v2 [16], *M. balbisiana* v1.1 [15] and *M. schizocarpa* v1 [13], *E. ventricosum* [20] and *Musella lasiocarpa* (in preparation) were also analyzed

(Supplementary Figure S3) and compared with RepeatExplorer2 following "comparative repeat analysis" protocol. The results were visualized by R script "plot_comparative_clustering_summary.R" (Fig, 4A).

SSR Tandem Repeats

The genome assembly was searched for SSR (microsatellite) motifs using the SSR mining pipeline developed by Biswas et al [48]. Searches were standardized for mining perfect SSRs from mono to hexa-nucleotide repeats (minimum repeat number of 12 for mononucleotides, 8 for di-, 5 for tri-, tetra- and penta-, and 4 repeats for penta- and hexa-nucleotides). SSRs abundance and nature was analyzed based on density in the genome (about 1 per 4000bp), array length (Figure 7B and Supplementary Table S14; SSR search parameters minimum lengths mono=1*12=12nt, di=8*2=16nt, tri=3*5=15nt, tetra=4*5=15nt, penta=5*4=20nt and hexa=6*4=24nt; total SSR count 123884; Class I>20nt and Class II≤20nt), nucleotide base composition of the SSR loci (70% were AT-rich) and abundance of each motif).

Fluorescent *in situ* hybridization (FISH)

Chromosome preparation and FISH was performed as described by Schwarzacher and Heslop-Harrison [123] with minor modifications. Plants of *E. glaucum*, *E. ventricosum*, *Musella lasiocarpa* (purchased commercially) and *M. balbisiana* 'Butuhan' (ITC1074) [124] were grown in the glasshouse at the University of Leicester, UK. Actively growing root tips were treated with 2 mM 8-hydroxyquinoline and fixed with 96% ethanol:glacial acetic acid (3:1). For chromosome preparations, roots were digested with a modified enzyme solution (32U/ml cellulose, Sigma-Aldrich C1184; 20U/ml 'Onozuka' RS cellulose; 35U/ml pectinase from *Aspergillus niger*, Sigma-Aldrich P4716; 20U/ml Viscozyme, Sigma-Alderich V2010) in 10mM citric acid/sodium citrate buffer (pH4.6) for 3-5h at 37°C and then kept in buffer for 12-

30h at 4°C. Meristems were dissected in 60% acetic acid and routinely 2-6 slide preparations were made from each root. Slides were stored at -20°C until FISH.

The 45S rDNA probe was labelled by random priming (Invitrogen) with digoxigenin dUTP or biotin dUTP (Roche) using the linearized clone pTa71 [125] containing the 45S rDNA repeat unit of *Triticum aestivum*. 50-100ng of labelled probe was used per slides and detection of hybridization sites was carried out with Fluorescein-conjugated anti-digoxigenin (Roche) or Streptavidin conjugated to Alexa-647 (Molecular Probes, Invitrogen). The remaining probes were designed from the consensus sequence of the centromeric repeat Egcen (Fig. 5) and the 5S rDNA (Fig. 7) or as simple sequence repeats (Fig. 6); as directly labelled oligonucleotides (200-500ng per slide) they needed no further detection and were as follows:

CenCy3: EGL_2640R: [Cyanine3]GAC CGT CGC ATT TTT TGG CGA AAC CAT GCT CGT ACG ACT TCC CAT GGG CTA AAA CGT TAG GA

CenFAM: EGL_G2640L: [6FAM]GGC CTA TAT TTT GAA ATT CCG AGA CGG TGC ATG AAA AAC CGA TCG AAA CGA AAC ATT GCG

5S_4M_Cy3: [Cyanine3]TCA GAA CTC CGA AGT TAA GCG TGC TTG GGC GAG AGT AGT AC

5S_3R_Cy3: [Cyanine3]AGT ACT AGG ATG GGT GAC CCC CTG GGA AGT CCT CGT GTT GC

5S_6L_Cy3: [Cyanine3]GCG ATC ATA CCA GCA CTA AAG CAC CGG ATC CCA TCA GAA CTC C

(AAG)15_FAM: [6FAM]AAG AAG AAG AAG AAG AAG AAG AAG AAG AAG AAG AAG AAG AAG AAG

(CT)23_TAMRA: [TAMRA]CTC TCT CTC TCT CTC TCT CTC TCT CTC TCT CTC TCT CTC TCT CTC T

For hybridization, probes were prepared in 40% (v/v) formamide, 20% (w/v) dextran sulphate, 2x SSC (sodium chloride sodium citrate), 0.03µg of salmon sperm DNA, 0.12% SDS (sodium dodecyl sulphate) and 0.12mM EDTA (ethylenediamine-tetra acetic acid). Chromosomes and 40-50µl of probe mixture were denatured together at 72°C for 8 mins,

cooled down slowly and allowed to hybridize overnight at 37°C. Post-hybridization washes were at 42°C in 0.1xSSC, giving a stringency of 80-85% for the short oligo probes, and 70-75% for the 45SrDNA probe. Chromosomes were counterstained with 4µg/ml DAPI (4´,6-diamidino-2-phenylindole) and mounted in CitifluorAF. Slides were examined using Nikon Eclipse 80i microscope and images were captured with a DS-QiMc monochrome camera, and NIS-Elements v2.34 (Nikon, Tokyo, Japan) assigning color and merging channels. Overlays of hybridization signal (shown in green or red) and DAPI images (in cyan or blue) were enhanced with Adobe Photoshop CC2018 using only cropping and functions that treat all pixels of the image. Seven FISH runs with different combinations of probes and replicates were performed, and between 5 and 15 metaphases per slide (99 metaphases in total from 15 slides) were analyzed in detail.

Data Availability

All supporting data can be found in the GigaScience database [127].

Raw sequence reads (RNA-seq, Illumina HiSeq, the Oxford Nanopore and Hi-C) were deposited in the NCBI under accession number: PRJNA736572. In specifically, the Oxford Nanopore raw reads: SRX11350424 and SRX11350425; RNA-seq raw reads, as follows: leaf: SRX11350426, root: SRX11350427; genomic Illumina short-read data: SRX11350423; raw reads of the Hi-C library: SRX11350428 and SRX11350429. The raw reads data were also deposited in Genome Sequence Archive (GSA) of the China National Center for Bioinformation (accession code: CRA004283).

The assembled genome was also deposited to GenBank in NCBI under the accession number: JAHSUZ000000000. Genome Assembly, gene and TE annotation data, transcriptomic data are also available on the Banana Genome Hub (http://banana-genome-hub.southgreen.fr/)

for download or exploration via a dedicated Genome Browser (Jbrowse) and syntenic browser (SynVisio).

Additional Files

Supplementary Figures

Pdf -word document containing Supplementary Figures S1-S12 containing

Supplementary Figure S1. Genomescope analysis of heterozygosity.

Supplementary Figure S2. GO enrichment terms.

Supplementary Figure S3. RepeatExplorer clustering summary in Musaceae species.

Supplementary Figure S4. LTR retroelement trees EGL and MAC.

Supplementary Figure S5. Gypsy and Copia insertion times in *Musa* and *E. glaucum*.

Supplementary Figure S6: Egcen FISH to *Musa* chromosomes.

Supplementary Figure S7. EgCen and *Nanica* in Assemblies of *E. glaucum* and *Musa*.

Supplementary Figure S8 Synteny of *E. glaucum* with *Musa* A and B genome.

Supplementary Figure S9. Dotplots of individual chromosomes.

Supplementary Figure S10. Inversions on chromosome 5.

Supplementary Figure S11. Length distribution of ONT reads.

Supplementary Figure S12. Hi-C interaction contact map.

Supplementary Tables

Excel Spreadsheet containing Supplementary Tables S1-S14

Supplementary Table S1. Contig statistics based on assembly of ONT sequencing data.

Supplementary Table S2. Chromosome lengths and number of contigs anchored in *Ensete glaucum* assembly.

Supplementary Table S3. Quality assessment of the gene annotation of *Ensete glaucum* using BUSCOs v5.

Supplementary Table S4. Complete gene list. homology and GO.

Supplementary Table S5. Statistics for shared orthogroups (OG) and gene clustering among *E. glaucum*, *E. ventricosum*, *Musa acuminata*, *M. balbisiana*, and *M. schizocarpa* genomes.

Supplementary Table S6. Positively selected genes and their annotation.

Supplementary Table S7: Result of gene family size change analysis.

Supplementary Table S8 a) Top 20 GO molecular function enrichments for *E. glaucum* and shared *E. glaucum/E. ventricosum* gene families; b) Top 20 GO biological pathways enrichments for *E. glaucum* and shared *E. glaucum/E. ventricosum* gene families.

Supplementary Table S9. Comparison of transcriptional factor between *Ensete glaucum* and *Musa acuminata.*

Supplementary Table S10. Transposable elements and other repeat proportions comparison in assembly (RepeatMasker).

Supplementary Table S11. Repeat content (RepeatExplorer) comparison between different Musaceae genomes.

Supplementary Table S12. Abundance of major tandemly repeated DNA repeats in Illumina raw reads.

Supplementary Table S13. Inferred centromere positions from locations of interrupted tandem arrays of the Egcen centromeric sequence on the chromosome assemblies.

Supplementary Table S14. Comparative survey of microsatellite sequences in *Ensete glaucum* genome with other sister species.

Declarations

List of abbreviations

BLAST: Basic Local Alignment Search Tool; bp: base pairs; BUSCO: Benchmarking Universal Single-Copy Orthologs; BWA: Burrows-Wheeler Aligner; DAPI: 4´,6-diamidino-2-phenylindole, Egcen: *Ensete glaucum* centromere sequence, FISH: fluorescence in situ hybridization; GeMoMa: Gene Model Mapper; Gb: gigabase pairs; GC: guanine-cytosine; GO: gene ontogeny; CTAB: cetyl trimethylammonium bromide; GWAS: Genome Wide Association Studies; Hi-C: High-throughput chromosome conformation capture; ITS: internal transcribed spacer of rDNA; kb: kilobase pairs; KEGG: Kyoto Encyclopedia of Genes and Genomes; GeMoMa: Gene Model Mapper; LACHESIS: Ligating Adjacent Chromatin Enables Scaffolding In Situ; LINE: long interspersed nucleotide elements; LTR: long terminal repeat; Mb: megabase pairs; ML: maximum likelihood; miRNA: microRNA; Mya: million years ago; NCBI: National Center for Biotechnology Information; NR: RefSeq non-redundant proteins; NOR: Nucleolar Organizing Region; NTS: non transcribed spacer of rDNA; ONT: Oxford Nanopore Technologies; PAML: Phylogenetic Analysis by Maximum Likelihood; PacBio: Pacific Biosciences; PASA: Program to Assemble Spliced Alignments; RAxML: Randomized Accelerated Maximum Likelihood; RNA-seq: RNA sequencing; rDNA: ribosomal DNA; SRA: Sequence Read Archive; SSR: Simple sequence repeat; TE: transposable element; TF: Transcription Factor; tRNA: transfer RNA; WGD: whole genome duplication.

Consent for publication

The origin of *E. glaucum* plants is given in Materials and Methods. They were collected in China and conserved in the South China Botanical Garden, Chinese Academy of Sciences, with appropriate agreements. No live material was exported out of the country. Other plants for chromosome preparations were obtained from the International Transit Centre, ITC genebank, with official Standard Material Transfer Agreement (SMTA), acknowledged in the manuscript.

Competing Interests

The authors declare that they have no competing interests.

Funding

This work was supported by grants from National Science Foundation of China (32070359), Guangdong Basic and Applied Basic Research Foundation (2021A1515012410), Overseas Distinguished Scholar Project of SCBG (Y861041001) and Undergraduate Innovation Training Program of Chinese Academy of Sciences (KCJH-80107-2020-004-97). M.R. acknowledges the support of the CGIAR Research Program on Roots, Tubers and Bananas (RTB).

Author contributions

Q.L. and J.S.H.H. designed the project and with M.R. and Z.W. contributed to project coordination. Z.W. and Q.H. collected samples and conducted DNA and RNA extraction. T.S. and J.S.H.H. conducted FISH experiments. Z.W. carried out genome assemblies; Z.W., M.R. G.D., and M.K.B. conducted gene annotation and comparative genomic analyses; Z.W., T.S., J.S.H.H. and F.C.B. conducted repetitive sequence analysis. All authors contributed to writing and editing the manuscript.

Acknowledgments

This work was technically supported by the high-performance cluster of the UMR AGAP - CIRAD of the South Green Bioinformatics Platform (http://www.southgreen.fr). Assistance and discussion with Celia Hansen and Paulina Tomaszewska, University of Leicester, are acknowledged. We thank Ye Yushi for the assistance with growing and maintaining the plants. We thank the International Musa Germplasm Transit Centre (https://www.bioversityinternational.org/banana-genebank/) for samples of *M. balbisiana*.

References

1. Wu Z, Raven PH, Hong D, Missouri Botanical Garden. Musaceae. 1. *Ensete*. *Flora of China*. Science Press and Missouri Botanical Garden Press. Beijing and St. Louis; 297–8, 2000.

2. Borrell JS, Biswas MK, Goodwin M, Blomme G, Schwarzacher T, Heslop-Harrison JS (Pat), et al. Enset in Ethiopia: a poorly characterized but resilient starch staple. *Ann Bot*. 2019; doi: 10.1093/aob/mcy214.

3. Zhao T, Zwaenepoel A, Xue J-Y, Kao S-M, Li Z, Schranz ME, et al. Whole-genome microsynteny-based phylogeny of angiosperms. *Nat Commun*. 2021; doi: 10.1038/s41467-021-23665-0.

4. Christelová P, Valárik M, Hřibová E, De Langhe E, Doležel J. A multi gene sequence-based phylogeny of the Musaceae (banana) family. *BMC Evol Biol*. 2011; doi: 10.1186/1471-2148-11-103.

5. Janssens SB, Vandelook F, De Langhe E, Verstraete B, Smets E, Vandenhouwe I, et al. Evolutionary dynamics and biogeography of Musaceae reveal a correlation between the

diversification of the banana family and the geological and climatic history of Southeast Asia. *New Phytol*. 2016; doi: 10.1111/nph.13856.

6. Cheesman EE. Classification of the Bananas: The Genus *Ensete* Horan. *Kew Bull*. 1947; doi: 10.2307/4109206.

7. Simmonds NW. Notes on Banana Taxonomy. *Kew Bull*. 1960; doi: 10.2307/4114778.

8. Li HW. Musaceae of Yunnan. *Acta phytotaxonomica sinica*. 1978.

9. Ochiai Y. From forests to homegardens: A case study of *Ensete glaucum* in Myanmar and Laos. *Tropics*. 2012; doi: 10.3759/tropics.21.59.

10. Song J-J, Liao J-P, Tang Y-J, Chen Z-Y. Chromosome numbers in Orchidantha (Lowiaceae) and their biogeographic and systematic implications. *Annales Botanici Fennici*. Finnish Zoological and Botanical Publishing Board; 41:429–332004.

11. Majumdar K, Sarkar A, Deb D, Majumder J, Datta B. Distribution record of *Ensete glaucum* (Roxb.) Cheesm.(Musaceae) in Tripura, Northeast India: a rare wild primitive banana. *Asian J Conserv Biol.* 2:164–72013.

12. Yang Q-S, Gao J, He W-D, Dou T-X, Ding L-J, Wu J-H, et al. Comparative transcriptomics analysis reveals difference of key gene expression between banana and plantain in response to cold stress. *BMC Genomics*. 2015; doi: 10.1186/s12864-015-1551-z.

13. Belser C, Istace B, Denis E, Dubarry M, Baurens F-C, Falentin C, et al. Chromosome-scale assemblies of plant genomes using nanopore long reads and optical maps. *Nature Plants*. 2018; doi: 10.1038/s41477-018-0289-4.

14. D'Hont A, Denoeud F, Aury J-M, Baurens F-C, Carreel F, Garsmeur O, et al. The banana (*Musa acuminata*) genome and the evolution of monocotyledonous plants. *Nature*. 2012; 488:213.

15. Wang Z, Miao H, Liu J, Xu B, Yao X, Xu C, et al. *Musa balbisiana* genome reveals subgenome evolution and functional divergence. *Nature Plants*. 2019; doi: 10.1038/s41477-019-0452-6.

16. Martin G, Baurens F-C, Droc G, Rouard M, Cenci A, Kilian A, et al. Improvement of the banana "*Musa acuminata*" reference sequence using NGS data and semi-automated bioinformatics methods. *BMC Genomics*. 2016; doi: 10.1186/s12864-016-2579-4.

17. Belser C, Baurens F-C, Noel B, Martin G, Cruaud C, Istace B, et al. Telomere-to-telomere gapless chromosomes of banana using nanopore sequencing. *Commun Biol*. 2021; doi: 10.1038/s42003-021-02559-3.

18. Droc G, Lariviere D, Guignon V, Yahiaoui N, This D, Garsmeur O, et al. The Banana Genome Hub. *Database*. 2013; doi: 10.1093/database/bat035.

19. Yemataw Z, Muzemil S, Ambachew D, Tripathi L, Tesfaye K, Chala A, et al. Genome sequence data from 17 accessions of *Ensete ventricosum*, a staple food crop for millions in Ethiopia. *Data in Brief*. 2018; doi: 10.1016/j.dib.2018.03.026.

20. Harrison J, Moore KA, Paszkiewicz K, Jones T, Grant MR, Ambacheew D, et al. A Draft Genome Sequence for *Ensete ventricosum*, the Drought-Tolerant "Tree Against Hunger." *Agronomy*. Multidisciplinary Digital Publishing Institute; 2014; doi: 10.3390/agronomy4010013.

21. Simão FA, Waterhouse RM, Ioannidis P, Kriventseva EV, Zdobnov EM. BUSCO: assessing genome assembly and annotation completeness with single-copy orthologs. *Bioinformatics*. 2015; doi: 10.1093/bioinformatics/btv351.

22. Pucker B. Mapping-based genome size estimation. bioRxiv 2019:607390. doi:10.1101/607390.

23. Bartos J, Alkhimova O, Dolezelová M, De Langhe E, Dolezel J. Nuclear genome size and genomic distribution of ribosomal DNA in *Musa* and *Ensete* (Musaceae): taxonomic implications. *Cytogenet Genome Res*. 2005; doi: 10.1159/000082381.

24. Wang R, Yang Y, Jing Y, Segar ST, Zhang Y, Wang G, et al. Molecular mechanisms of mutualistic and antagonistic interactions in a plant-pollinator association. *Nat Ecol Evol*. 2021; doi: 10.1038/s41559-021-01469-1.

25. González AV, Gómez-Silva V, Ramírez MJ, Fontúrbel FE. Meta-analysis of the differential effects of habitat fragmentation and degradation on plant genetic diversity. *Conserv Biol*. 2020; doi: 10.1111/cobi.13422.

26. Liu A-Z, Kress WJ, Wang HF, Li D. Insect pollination of *Musella* (Musaceae), a monotypic genus endemic to Yunnan, China. *Plant Syst and Evol*. 235:135–462002;

27. Sardos J, Breton C, Perrier X, Houwe IVD, Paofa J, Rouard M, et al. Wild to domesticates: genomes of edible diploid bananas hold traces of several undefined genepools. *bioRxiv*. 2021; doi: 10.1101/2021.01.29.428762.

28. Martin G, Cardi C, Sarah G, Ricci S, Jenny C, Fondi E, et al. Genome ancestry mosaics reveal multiple and cryptic contributors to cultivated banana. *Plant J*. 2020; doi: 10.1111/tpj.14683.

29. Maughan PJ, Lee R, Walstead R, Vickerstaff RJ, Fogarty MC, Brouwer CR, et al. Genomic insights from the first chromosome-scale assemblies of oat (*Avena* spp.) diploid species. *BMC Biol*. 2019; doi: 10.1186/s12915-019-0712-y.

30. Marrano A, Britton M, Zaini PA, Zimin AV, Workman RE, Puiu D, et al. High-quality chromosome-scale assembly of the walnut (*Juglans regia* L.) reference genome. *Gigascience*. 2020; doi: 10.1093/gigascience/giaa050.

31. Yang X, Kang M, Yang Y, Xiong H, Wang M, Zhang Z, et al. A chromosome-level genome assembly of the Chinese tupelo *Nyssa sinensis*. *Sci Data*. 2019; doi: 10.1038/s41597-019-0296-y.

32. Voillemot M, Pannell JR. Inbreeding depression is high in a self-incompatible perennial herb population but absent in a self-compatible population showing mixed mating. *Ecol Evol*. 2017; doi: 10.1002/ece3.3354.

33. Sun G, Xu Y, Liu H, Sun T, Zhang J, Hettenhausen C, et al. Large-scale gene losses underlie the genome evolution of parasitic plant *Cuscuta australis*. *Nat Commun*. 2018; doi: 10.1038/s41467-018-04721-8.

34. Redwan RM, Saidin A, Kumar SV. The draft genome of MD-2 pineapple using hybrid error correction of long reads. *DNA Res*. 2016; doi: 10.1093/dnares/dsw026.

35. Franco-Zorrilla JM, López-Vidriero I, Carrasco JL, Godoy M, Vera P, Solano R. DNA-binding specificities of plant transcription factors and their potential to define target genes. *Proc Natl Acad Sci U S A*. 2014; doi: 10.1073/pnas.1316278111.

36. Cenci A, Guignon V, Roux N, Rouard M. Genomic analysis of NAC transcription factors in banana (*Musa acuminata*) and definition of NAC orthologous groups for monocots and dicots. *Plant Mol Biol*. 2014; doi: 10.1007/s11103-013-0169-2.

37. Xiao Y-Y, Kuang J-F, Qi X-N, Ye Y-J, Wu Z-X, Chen J-Y, et al. A comprehensive investigation of starch degradation process and identification of a transcriptional activator MabHLH6 during banana fruit ripening. *Plant Biotechnol J*. 2018; doi: 10.1111/pbi.12756.

38. Lerat E. Identifying repeats and transposable elements in sequenced genomes: how to find your way through the dense forest of programs. *Heredity (Edinb)*. 2010; doi: 10.1038/hdy.2009.165.

39. Novák P, Neumann P, Macas J. Global analysis of repetitive DNA from unassembled sequence reads using RepeatExplorer2. *Nat Protoc*. 2020; doi: 10.1038/s41596-020-0400-y.

40. Wu W, Yang Y-L, He W-M, Rouard M, Li W-M, Xu M, et al. Whole genome sequencing of a banana wild relative *Musa itinerans* provides insights into lineage-specific diversification of the Musa genus. *Sci Rep*. 2016; doi: 10.1038/srep31586.

41. Biscotti MA, Olmo E, Heslop-Harrison JSP. Repetitive DNA in eukaryotic genomes. *Chromosome Res*. 2015; doi: 10.1007/s10577-015-9499-z.

42. Heslop-Harrison JSP, Schwarzacher T. Organisation of the plant genome in chromosomes. *Plant J*. 2011; doi: 10.1111/j.1365-313X.2011.04544.x.

43. Čížková J, Hřibová E, Humplíková L, Christelová P, Suchánková P, Doležel J. Molecular Analysis and Genomic Organization of Major DNA Satellites in Banana (*Musa* spp.). *PLoS ONE*. 2013; doi: 10.1371/journal.pone.0054808.

44. Suntronpong A, Kugou K, Masumoto H, Srikulnath K, Ohshima K, Hirai H, et al. CENP-B box, a nucleotide motif involved in centromere formation, occurs in a New World monkey. *Biol Lett*. 2016; doi: 10.1098/rsbl.2015.0817.

45. Aragón-Alcaide L, Miller T, Schwarzacher T, Reader S, Moore G. A cereal centromeric sequence. *Chromosoma*. 1996; doi: 10.1007/BF02524643.

46. Heslop-Harrison JS, Murata M, Ogura Y, Schwarzacher T, Motoyoshi F. Polymorphisms and genomic organization of repetitive DNA from centromeric regions of *Arabidopsis* chromosomes. *Plant Cell*. 1999; doi: 10.1105/tpc.11.1.31.

47. Lermontova I, Sandmann M, Mascher M, Schmit AC, Chaboute M-E. Centromeric chromatin and its dynamics in plants. *Plant J*. 2015; doi: 10.1111/tpj.12875.

48. Biswas MK, Natarajan S, Biswas D, Nath UK, Park J-I, Nou I-S. LSAT: Liliaceae Simple Sequences Analysis Tool, a web server. *Bioinformation*. 2018; doi: 10.6026/97320630014181.

49. Biswas MK, Darbar JN, Borrell JS, Bagchi M, Biswas D, Nuraga GW, Demissew S, Wilkin P, Schwarzacher T, Heslop-Harrison JS. The landscape of microsatellites in the enset (*Ensete ventricosum*) genome and web- based marker resource development. *Sci Rep*. 2020; doi: 10.1038

50. Liu Q, Li X, Zhou X, et al. The repetitive DNA landscape in Avena (Poaceae): chromosome and genome evolution defined by major repeat classes in whole-genome sequence reads. Bmc Plant Biol 2019;19(1). doi:10.1186/s12870-019-1769-z.

/s41598-020-71984-x.

51. Goffová I, Fajkus J. The rDNA Loci—Intersections of Replication, Transcription, and Repair Pathways. Int J Mol Sci 2021;22(3):1302. doi:10.3390/ijms22031302.

52. Tulpová Z, Kovařík A, Toegelová H, et al. Anatomy, transcription dynamics and evolution of wheat ribosomal RNA loci deciphered by a multi-omics approach. bioRxiv 2021:2020.08.29.273623. doi:10.1101/2020.08.29.273623.

53. Osuji JO, Crouch J, Harrison G, Heslop-Harrison JS. Molecular Cytogenetics of *Musa* Species, Cultivars and Hybrids: Location of 18S-5.8S-25S and 5S rDNA and Telomere-like Sequences. *Ann Bot*. 1998; doi: 10.1006/anbo.1998.0674.

54. Baurens F-C, Noyer J-L, Lanaud C, Lagoda PJL. Assessment of a species-specific element (Brep 1) in banana. *Theor Appl Genet*. 1997; doi: 10.1007/s001220050643.

55. Garcia S, Wendel JF, Borowska-Zuchowska N, Aïnouche M, Kuderova A, Kovarik A. The Utility of Graph Clustering of 5S Ribosomal DNA Homoeologs in Plant Allopolyploids, Homoploid Hybrids, and Cryptic Introgressants. *Front Plant Sci*. 2020; doi: 10.3389/fpls.2020.00041.

56. Castilho A, Heslop-Harrison JS. Physical mapping of 5S and 18S-25S rDNA and repetitive DNA sequences in *Aegilops umbellulata*. *Genome*. 1995; doi: 10.1139/g95-011.

57. Dubcovsky J, Dvorák J. Ribosomal RNA multigene loci: nomads of the Triticeae genomes. *Genetics*. 1995; doi: 10.1093/genetics/140.4.1367.

58. Bandi V, Gutwin C. Interactive Exploration of Genomic Conservation. *46th Graphics Interface Conference on Proceedings of Graphics Interface 2020*. Waterloo, Canada: Canadian Human-Computer Communications Society.

59. Li W, Challa GS, Zhu H, Wei W. Recurrence of chromosome rearrangements and reuse of DNA breakpoints in the evolution of the Triticeae genomes. *G3 (Bethesda)*. 2016; doi: 10.1534/g3.116.035089.

60. Lewin HA, Richards S, Lieberman Aiden E, et al. The Earth BioGenome Project 2020: Starting the clock. *Proc Natl Acad Sci U S A*. 2022;119(4):e2115635118. doi:10.1073/pnas.2115635118.

61. GBIF.org: GBIF. https://doi.org/10.15468/dl.f9meez (2021). Accessed 2021 Apr 25.

62. POWO: Plants of the World Online | Kew Science. Plants of the World Online. http://www.plantsoftheworldonline.org/ Accessed 2021 Sep 3.

63. Bolger AM, Lohse M, Usadel B. Trimmomatic: a flexible trimmer for Illumina sequence data. *Bioinformatics*. 2014; doi: 10.1093/bioinformatics/btu170.

64. Andrews S. Babraham Bioinformatics—FastQC A Quality Control Tool for High Throughput Sequence Data. https://www.bioinformatics.babraham.ac.uk/projects/fastqc/

65. Chen S, Zhou Y, Chen Y, Gu J. fastp: an ultra-fast all-in-one FASTQ preprocessor. *Bioinformatics*. 2018; doi: 10.1093/bioinformatics/bty560.

66. Belton J-M, McCord RP, Gibcus JH, Naumova N, Zhan Y, Dekker J. Hi-C: a comprehensive technique to capture the conformation of genomes. *Methods*. 2012; doi: 10.1016/j.ymeth.2012.05.001.

67. Belaghzal H, Dekker J, Gibcus JH. Hi-C 2.0: An optimized Hi-C procedure for high-resolution genome-wide mapping of chromosome conformation. *Methods*. 2017; doi: 10.1016/j.ymeth.2017.04.004.

68. NextDenovo. https://github.com/Nextomics/NextDenovo. Accessed 2019 Oct 26.

69. Liu H, Wu S, Li A, Ruan J, Wu S, Li A, et al. SMARTdenovo: a de novo assembler using long noisy reads. *Gigabyte*. 2021; doi: 10.46471/gigabyte.15.

70. Vaser R, Sović I, Nagarajan N, Šikić M. Fast and accurate de novo genome assembly from long uncorrected reads. *Genome Res*. 2017; doi: 10.1101/gr.214270.116.

71. Li H, Durbin R. Fast and accurate long-read alignment with Burrows-Wheeler transform. *Bioinformatics*. 2010; doi: 10.1093/bioinformatics/btp698.

72. Hu J, Fan J, Sun Z, Liu S. NextPolish: a fast and efficient genome polishing tool for long-read assembly. *Bioinformatics*. 2020; doi: 10.1093/bioinformatics/btz891.

73. Walker BJ, Abeel T, Shea T, Priest M, Abouelliel A, Sakthikumar S, et al. Pilon: an integrated tool for comprehensive microbial variant detection and genome assembly improvement. *PLoS One*. 2014; doi: 10.1371/journal.pone.0112963.

74. Langmead B, Salzberg SL. Fast gapped-read alignment with Bowtie 2. *Nat Methods*. 2012; doi: 10.1038/nmeth.1923.

75. Servant N, Varoquaux N, Lajoie BR, Viara E, Chen C-J, Vert J-P, et al. HiC-Pro: an optimized and flexible pipeline for Hi-C data processing. *Genome Biol*. 2015; doi: 10.1186/s13059-015-0831-x.

76. Burton JN, Adey A, Patwardhan RP, Qiu R, Kitzman JO, Shendure J. Chromosome-scale scaffolding of de novo genome assemblies based on chromatin interactions. *Nat Biotechnol*. 2013; doi: 10.1038/nbt.2727.

77. Grabherr MG, Haas BJ, Yassour M, Levin JZ, Thompson DA, Amit I, et al. Trinity: reconstructing a full-length transcriptome without a genome from RNA-Seq data. *Nat Biotechnol*. 2011; doi: 10.1038/nbt.1883.

78. Haas BJ, Delcher AL, Mount SM, Wortman JR, Smith RK, Hannick LI, et al. Improving the Arabidopsis genome annotation using maximal transcript alignment assemblies. *Nucleic Acids Res*. 2003; doi: 10.1093/nar/gkg770.

79. Marçais G, Kingsford C. A fast, lock-free approach for efficient parallel counting of occurrences of k-mers. *Bioinformatics*. 2011; doi: 10.1093/bioinformatics/btr011.

80. Sun H, Ding J, Piednoël M, Schneeberger K. findGSE: estimating genome size variation within human and Arabidopsis using k-mer frequencies. *Bioinformatics*. 2018; doi: 10.1093/bioinformatics/btx637.

81. Ranallo-Benavidez TR, Jaron KS, Schatz MC. GenomeScope 2.0 and Smudgeplot for reference-free profiling of polyploid genomes. *Nat Commun*. 2020; doi: 10.1038/s41467-020-14998-3.

82. Dobin A, Davis CA, Schlesinger F, Drenkow J, Zaleski C, Jha S, et al. STAR: ultrafast universal RNA-seq aligner. *Bioinformatics*. 2013; doi: 10.1093/bioinformatics/bts635.

83. Brůna T, Hoff KJ, Lomsadze A, Stanke M, Borodovsky M. BRAKER2: automatic eukaryotic genome annotation with GeneMark-EP+ and AUGUSTUS supported by a protein database. NAR Genom Bioinform. 2021 Jan 6;3(1):lqaa108. doi: 10.1093/nargab/lqaa108.

84. Lomsadze A, Burns PD, Borodovsky M. Integration of mapped RNA-Seq reads into automatic training of eukaryotic gene finding algorithm. *Nucleic Acids Res*. 2014; doi: 10.1093/nar/gku557.

85. Stanke M, Diekhans M, Baertsch R, Haussler D. Using native and syntenically mapped cDNA alignments to improve de novo gene finding. *Bioinformatics*. 2008; doi: 10.1093/bioinformatics/btn013.

86. Campbell MS, Law M, Holt C, Stein JC, Moghe GD, Hufnagel DE, et al. MAKER-P: a tool kit for the rapid creation, management, and quality control of plant genome annotations. *Plant Physiol*. 2014; doi: 10.1104/pp.113.230144.

87. Keilwagen J, Hartung F, Grau J. GeMoMa: Homology-based gene prediction utilizing intron position conservation and RNA-seq data. *Methods Mol Biol*. 2019; doi: 10.1007/978-1-4939-9173-0_9.

88. Haas BJ, Salzberg SL, Zhu W, Pertea M, Allen JE, Orvis J, et al. Automated eukaryotic gene structure annotation using EVidenceModeler and the Program to Assemble Spliced Alignments. *Genome Biol*. 2008; doi: 10.1186/gb-2008-9-1-r7.

89. Camacho C, Coulouris G, Avagyan V, Ma N, Papadopoulos J, Bealer K, et al. BLAST+: architecture and applications. *BMC Bioinformatics*. 2009; doi: 10.1186/1471-2105-10-421.

90. Magrane M, Consortium U. UniProt Knowledgebase: a hub of integrated protein data. *Database (Oxford)*. 2011; doi: 10.1093/database/bar009.

91. Zdobnov EM, Apweiler R. InterProScan - an integration platform for the signature-recognition methods in InterPro. *Bioinformatics*. 2001; doi: 10.1093/bioinformatics/17.9.847.

92. Conesa A, Götz S, García-Gómez JM, Terol J, Talón M, Robles M. Blast2GO: a universal tool for annotation, visualization and analysis in functional genomics research. *Bioinformatics*. 2005; doi: 10.1093/bioinformatics/bti610.

93. Droc G: ensete_annotation. https://github.com/gdroc/ensete_annotation (2021). Accessed 2021 Oct 25.

94. Emms DM, Kelly S. OrthoFinder: solving fundamental biases in whole genome comparisons dramatically improves orthogroup inference accuracy. *Genome Biol*. 2015; doi: 10.1186/s13059-015-0721-2.

95. Buchfink B, Xie C, Huson DH. Fast and sensitive protein alignment using DIAMOND. *Nat Methods*. 2015; doi: 10.1038/nmeth.3176.

96. Lex A, Gehlenborg N, Strobelt H, Vuillemot R, Pfister H. UpSet: Visualization of Intersecting Sets. *IEEE Trans Vis Comput Graph*. 2014; doi: 10.1109/TVCG.2014.2346248.

97. Alexa A, Rahnenfuhrer J. topGO: enrichment analysis for gene ontology. R package version 2.24.0. 2010.

98. Hazzouri KM, Gros-Balthazard M, Flowers JM, Copetti D, Lemansour A, Lebrun M, et al. Genome-wide association mapping of date palm fruit traits. *Nat Commun*. 2019; doi: 10.1038/s41467-019-12604-9.

99. Ouyang S, Zhu W, Hamilton J, Lin H, Campbell M, Childs K, et al. The TIGR Rice Genome Annotation Resource: improvements and new features. *Nucleic Acids Res*. 2007; doi: 10.1093/nar/gkl976.

100. Goodstein DM, Shu S, Howson R, Neupane R, Hayes RD, Fazo J, et al. Phytozome: a comparative platform for green plant genomics. *Nucleic Acids Res*. 2012; doi: 10.1093/nar/gkr944.

101. Han MV, Thomas GWC, Lugo-Martinez J, Hahn MW. Estimating gene gain and loss rates in the presence of error in genome assembly and annotation using CAFE 3. *Mol Biol Evol*. 2013; doi: 10.1093/molbev/mst100.

102. Zheng Y, Jiao C, Sun H, Rosli HG, Pombo MA, Zhang P, et al. iTAK: A Program for Genome-wide Prediction and Classification of Plant Transcription Factors, Transcriptional Regulators, and Protein Kinases. *Mol Plant*. 2016; doi: 10.1016/j.molp.2016.09.014.

103. Sun P, Jiao B, Yang Y, Shan L, Li T, Li X, et al. WGDI: A user-friendly toolkit for evolutionary analyses of whole-genome duplications and ancestral karyotypes. 2021; doi: 10.1101/2021.04.29.441969.

104. Li H. Minimap2: pairwise alignment for nucleotide sequences. *Bioinformatics*. 2018; doi: 10.1093/bioinformatics/bty191.

105. Cabanettes F, Klopp C. D-GENIES: dot plot large genomes in an interactive, efficient and simple way. *PeerJ*. 2018; doi: 10.7717/peerj.4958.

106. Wang Y, Tang H, DeBarry JD, Tan X, Li J, Wang X, et al. MCScanX: a toolkit for detection and evolutionary analysis of gene synteny and collinearity. *Nucleic Acids Res*. 2012; doi: 10.1093/nar/gkr1293.

107. Amselem J, Cornut G, Choisne N, Alaux M, Alfama-Depauw F, Jamilloux V, et al. RepetDB: a unified resource for transposable element references. *Mob DNA*. 2019; doi: 10.1186/s13100-019-0150-y.

108. Belser C: Pahang-associated-data. GitHub. https://github.com/institut-de-genomique/Pahang-associated-data (2021). Accessed 2021 Oct 25.

109. Ou S, Su W, Liao Y, et al. Benchmarking transposable element annotation methods for creation of a streamlined, comprehensive pipeline. *Genome Biol*. 2019;20(1):275.

110. Ellinghaus D, Kurtz S, Willhoeft U. LTRharvest, an efficient and flexible software for de novo detection of LTR retrotransposons. *BMC Bioinformatics*. 2008; doi: 10.1186/1471-2105-9-18.

111. Ou S, Jiang N. LTR_retriever: A Highly Accurate and Sensitive Program for Identification of Long Terminal Repeat Retrotransposons. *Plant Physiol*. 2018; doi: 10.1104/pp.17.01310.

112. Xu Z, Wang H. LTR_FINDER: an efficient tool for the prediction of full-length LTR retrotransposons. *Nucleic Acids Res*. 2007; doi: 10.1093/nar/gkm286.

113. Su W, Gu X, Peterson T. TIR-Learner, a new ensemble method for TIR Transposable Element Annotation, Provides Evidence for abundant new transposable elements in the maize genome. *Mol Plant*. 2019; doi: 10.1016/j.molp.2019.02.008.

114. Shi J, Liang C. Generic Repeat Finder: A high-sensitivity tool for genome-wide *de novo* repeat detection. *Plant Physiol*. 2019; doi: 10.1104/pp.19.00386.

115. Xiong W, He L, Lai J, Dooner HK, Du C. HelitronScanner uncovers a large overlooked cache of Helitron transposons in many plant genomes. *Proc Natl Acad Sci U S A*. 2014; doi: 10.1073/pnas.1410068111.

116. Flynn JM, Hubley R, Goubert C, Rosen J, Clark AG, Feschotte C, et al. RepeatModeler2 for automated genomic discovery of transposable element families. *Proc Natl Acad Sci U S A*. 2020; doi: 10.1073/pnas.1921046117.

117. Flutre T, Duprat E, Feuillet C, et al. Considering Transposable Element Diversification in De Novo Annotation Approaches. Plos One. 2011;6(1):e16526.

118. Fu L, Niu B, Zhu Z, Wu S, Li W. CD-HIT: accelerated for clustering the next-generation sequencing data. *Bioinformatics*. 2012; doi: 10.1093/bioinformatics/bts565.

119. Zhang R-G, Wang Z-X, Ou S, Li G-Y. TEsorter: lineage-level classification of transposable elements using conserved protein domains. Accessed 2019 Oct 15.

120. Edgar RC. MUSCLE: multiple sequence alignment with high accuracy and high throughput. *Nucleic Acids Res*. 2004; doi: 10.1093/nar/gkh340.

121. Ma J, Bennetzen JL. Rapid recent growth and divergence of rice nuclear genomes. *Proc Natl Acad Sci U S A*. 2004; doi: 10.1073/pnas.0403715101.

122. Wang Z: LTR-insertion-time-estimation. GitHub. https://github.com/wangziwei08/LTR-insertion-time-estimation/(2021). Accessed 2021 Oct 25.

123. Schwarzacher T, Heslop-Harrison JS. Practical *in situ* Hybridization. Oxford, UK: BIOS Scientific Publishers Ltd; 2000.

124. Ruas M, Guignon V, Sempere G, Sardos J, Hueber Y, Duvergey H, et al. MGIS: managing banana (*Musa* spp.) genetic resources information and high-throughput genotyping data. *Database*. 2017; doi: 10.1093/database/bax046.

125. Gerlach WL, Bedbrook JR. Cloning and characterization of ribosomal RNA genes from wheat and barley. *Nucleic Acids Res*. 1979;7(7):1869-1885. doi:10.1093/nar/7.7.1869.

126. Manchester SR, Kress WJ. Fossil bananas (Musaceae): Ensete oregonense sp. nov. from the Eocene of western North America and its phytogeographic significance. *Am J Bot*. 1993;80(11):1264-1272. doi:https://doi.org/10.1002/j.1537-2197.1993.tb15363.x.

127. Wang Z; Rouard M; Biswas MK; Droc G; Cui D; Roux N; Baurens F; Ge X; Schwarzacher T; Heslop-Harrison JS; Liu Q. Supporting data for "A chromosome-level reference genome of Ensete glaucum gives insight into diversity, chromosomal and repetitive sequence evolution in the Musaceae" GigaScience Database. 2022;http://doi.org/10.5524/102198

Figure legends

Figure 1. *Ensete glaucum* plant morphology and distribution map.

(A) *E. glaucum* in South China Botanical Garden, Chinese Academy of Sciences. The pseudo-stem of this plant is about 3.5m tall. (B) Inflorescence with male and female flowers showing bracts and flowers alternately arranged along the main axis. (C) Staminate flowers, and visiting black shield wasp (*Vespa bicolor*, Vespidae, Hymenoptera) (D) Female flowers. (E) Fruits. Bars represent 5 cm in B. 2 cm in C, D and 1cm in E. (F) Native distribution of *Ensete glaucum*, *E. superbum*, *Musa* and *Ensete* species by countries or provinces (for China, India-Assam, and Australia). Musaceae are not currently native in the Americas, although *Ensete* is present in the fossil record [126]. *E. glaucum* always occurs in the same provinces as *Musa* and sometimes with other Asian *Ensete* species. Map adapted from POWO [62].

Figure 2. *Ensete glaucum* chromosome assembly and genome features.

Circos plot of (a) The nine pseudo-molecules of the EGL assembly corresponding to the nine chromosomes. Black dots indicate centromere positions and 5S and 45S the positions of rDNA loci; scale in Mbp; (b) Gene density; (c) Repeat density; (d) *Copia* LTR retroelement density; (e) *Gypsy* LTR retroelement density; (f) DNA transposon density; (g) Simple sequence repeat (microsatellite) density; (h) Syntenic genomic blocks, linked by curved lines (arbitrary color) in middle of the plot.

Figure 3. Gene family evolution and conservation.

(A) The synonymous substitutions (*K*s) frequency density distributions of orthologs within EGL or MAC, whose peaks indicate whole-genome duplications (WGDs). (B) Intersection diagram showing the distribution of shared orthogroups (OGs) (at least two sequences per OG)

among *Musa* and *Ensete* genomes. Codes: E, *E. glaucum*; V, *E. ventricosum*; A, *M. acuminata*; B, *M. balbisiana*; S, *M. schizocarpa.* (C) Gene family expansion and contraction with a phylogenetic tree showing timeline of divergence of monocot species. MRCA: Most Recent Common Ancestor. Numbers denote the gene family expansion (orange) and contraction (green). (D) Histogram of the comparative abundance （number of genes） of transcriptions factors between *M. acuminata* and *E. glaucum.*

Figure 4. Comparative analysis of repetitive DNA in Musaceae using RepeatExplorer.

(A) Bar chart showing the sizes (numbers of reads) of the most abundant individual graph-based read clusters (upper part; black bars) and display of their distribution among six Musaceae species (coloured rectangle sizes in lower part proportional to the number of reads in a cluster for each species, based on the annotation of the clusters). Clusters and species were sorted by using hierarchical clustering. Species codes: EGL, *Ensete glaucum*; EVE, *E. ventricosum*; MAC, *Musa acuminata*; MBA, *M. balbisiana*; MLA, *Musella lasiocarpa*; MSC, *M. schizocarpa*. (B) The distribution of insertion times of LTR retroelements (members of *Copia* and *Gypsy* classes) in *E. glaucum.* (C) The ages of total LTR- retroelement insertions in *E. glaucum, M. acuminata* and *M. balbisiana*. Mya = Million years ago.

Figure 5. *Ensete glaucum* centromeric repeat structure.

(A) Dot plot (self-comparison of sequences) showing start of a 134bp Egcen tandem array. (B) Dot plot showing part of a chromosomes assembly (eg04) plotted against part of a single ONT read with blocks of the Egcen tandem repeat (appearing as dense rectangles at this scale) interspersed with *Nanica* elements (red; five homologous copies in both orientations) and LTR retroelements (green; two non-homologous sub-families), (C) Bar chart showing frequency distribution of the Egcen centromeric tandem repeat, *Nanica* transposable elements (x10 on axis), and locations of 45S and 5S rDNA along the assemblies for each pseudo-chromosome.

Long Egcen arrays occur at one of more sites at the centromeric regions of all chromosomes. (D, E, F) *In situ* hybridization of Egcen probe detected by red fluorescence to cyan-fluorescing DAPI-stained chromosomes of (D) EGL, *Ensete glaucum*; (E) EVE, *E. ventricosum*; and (F) MLA, *Musella lasiocarpa*. The red Egcen signals collocate with the primary centromeric constriction on all 9 pairs of chromosomes. Bar=5µm.

Figure 6. Microsatellite (SSR) distribution in *Ensete glaucum*.

（A） Abundance (count) and total number of monomers of microsatellites (SSR) with motifs between 1 and 6 bp long. (B, C） *In situ* hybridization of synthetic microsatellite probes to DAPI-stained (blue) chromosomes, showing (B) AAG is relatively uniformly distributed along chromosomes compared to (C) where the greater abundance of AG/CT in distal chromosome regions is seen. （D) Abundance of AAG, AG, all microsatellites，and genes along the chromosome assemblies. In agreement with the *in situ* hybridization result, AAG is more uniformly distributed, while AG (along with genes and all the microsatellites pooled) show greater abundance in distal chromosome regions except for the arm of chromosome eg06 carrying the 45S rDNA (NOR). Bar = 5µm.

Figure 7. rDNA in *Ensete glaucum*

(A-C) *In situ* hybridization to chromosomes (stained blue with DAPI) showing locations of (A) the 45S rDNA (green) on one pair of chromosomes (eg06) while Egcen (red) is located at the centromeres of all nine chromosome pairs. Unspecific signal is marked by x. （B) Chromosomes showing location of 45S rDNA loci (green on eg06; the two sites are on two chromosomes which are adjacent to each other and the two loci have fused); the 5S rDNA loci (red) are located near the end of one chromosome pair eg05). （C) The 5S rDNA (red on eg05) in a more dispersed pattern with Egcen (green) at all 9 pairs of centromeres; inset shows 5S rDNA chromosomes at higher magnification. The 5S rDNA signal is dispersed over a longer

region of the chromosome, while the 45S rDNA locus is dense and occupies much of the chromosome arm. Bar = 5µm. X=stain precipitate. (D) Histogram showing density of genes (light green), Egcen (blue) and 45S rDNA copies (red) on chromosome eg06. The arm carrying the 45S rDNA is depleted in protein-coding genes. (E) Part of a single ONT read covering 24kb spanning part of the 5S rDNA array. The unusually long 1056bp tandemly repeated 5S rDNA monomers (14 copies) are interrupted by an LTR retroelement. LTRs, with no homology to the 5S rDNA, are seen (bottom left and top right) in the red box.

Figure 8. Synteny of *Ensete glaucum* and *Musa acuminata*

(A) Synteny plot（Synvisio）connecting syntenic genes in the nine chromosomes of *E. glaucum* (egxx) and 11 chromosomes of *M. acuminata* (maxx). Syntenic blocks of high homology are indicated by uniformly colored areas in the graphs. Only eg05 and ma05 maintain synteny over the full chromosome length, although there are some rearrangements (s. Three ma chromosomes are represented by part of one eg chromosome, while other ma chromosomes are fusions of more than one eg chromosome. （B) Dot plot comparing DNA sequences of *E. glaucum* and *M. acuminata* (for more detailed dot-plots see Supplementary Figure S9).（C) Representation of the syntenic blocks in the karyotypes of *E. glaucum* and *M. acuminata*. Chromosome rearrangements are shown, complementing the Synteny plot, while inversions and relative expansions and contractions of genome regions are clear.

 Click here to access/download;Figure;Fig1 Revised 2022-01-24.pdf

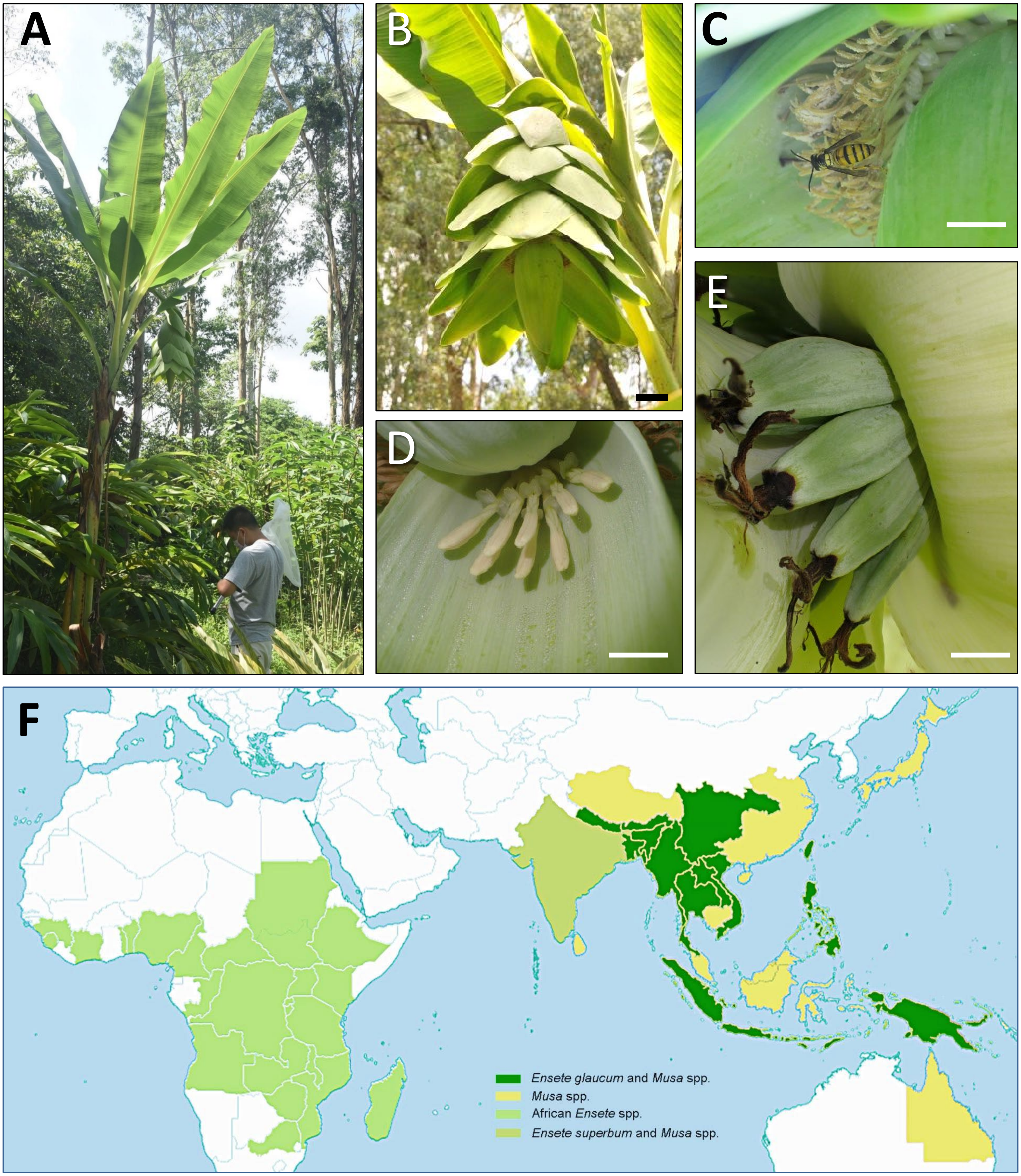

Fig 2

Click here to access/download;Figure;Fig2 Revised 2022-01-24.pdf

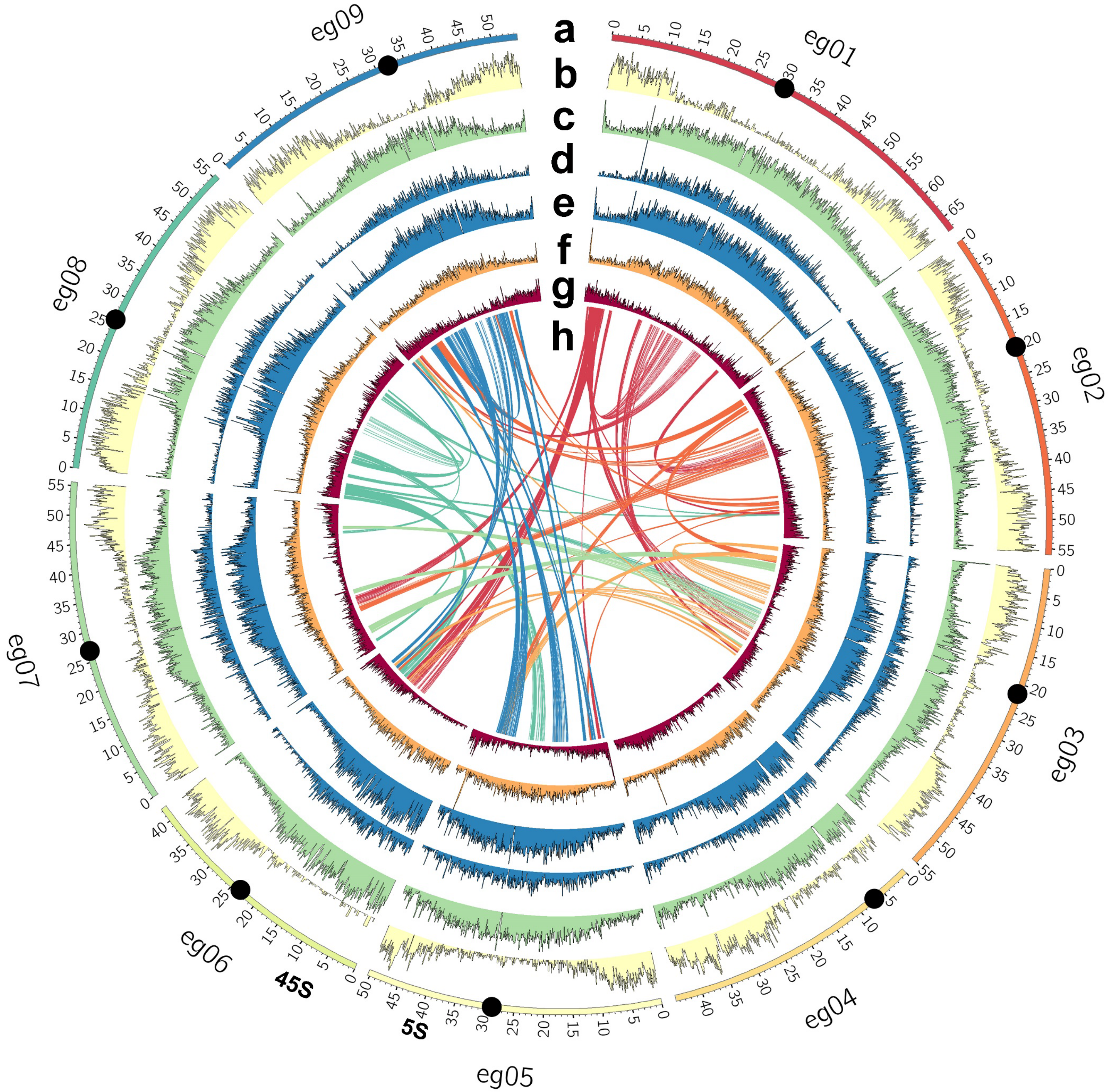

Fig 3
Click here to access/download;Figure;Fig3 Revised 2022-01-24.pdf

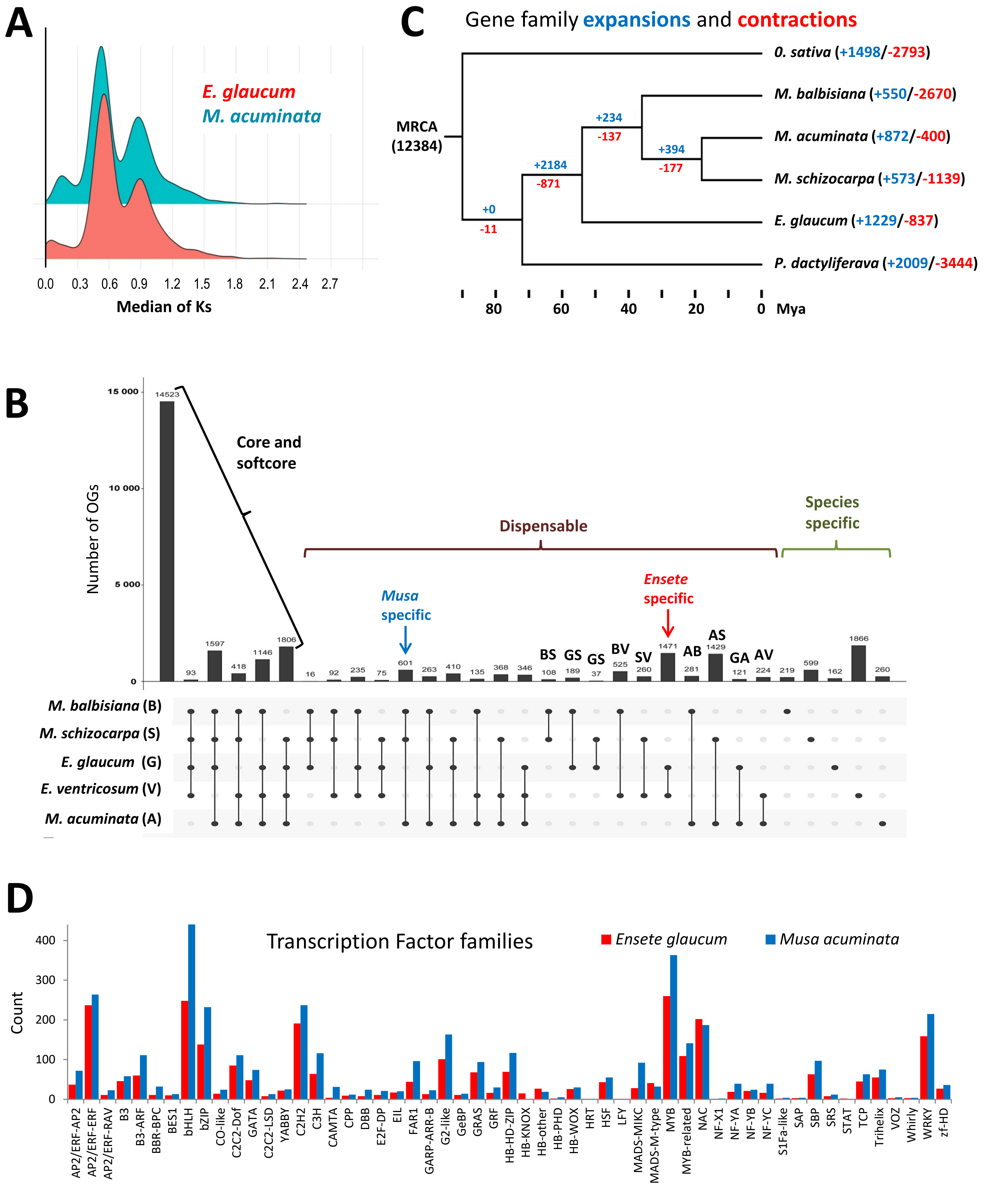

Click here to access/download;Figure;Fig4 Revised 2022-01-24.pdf

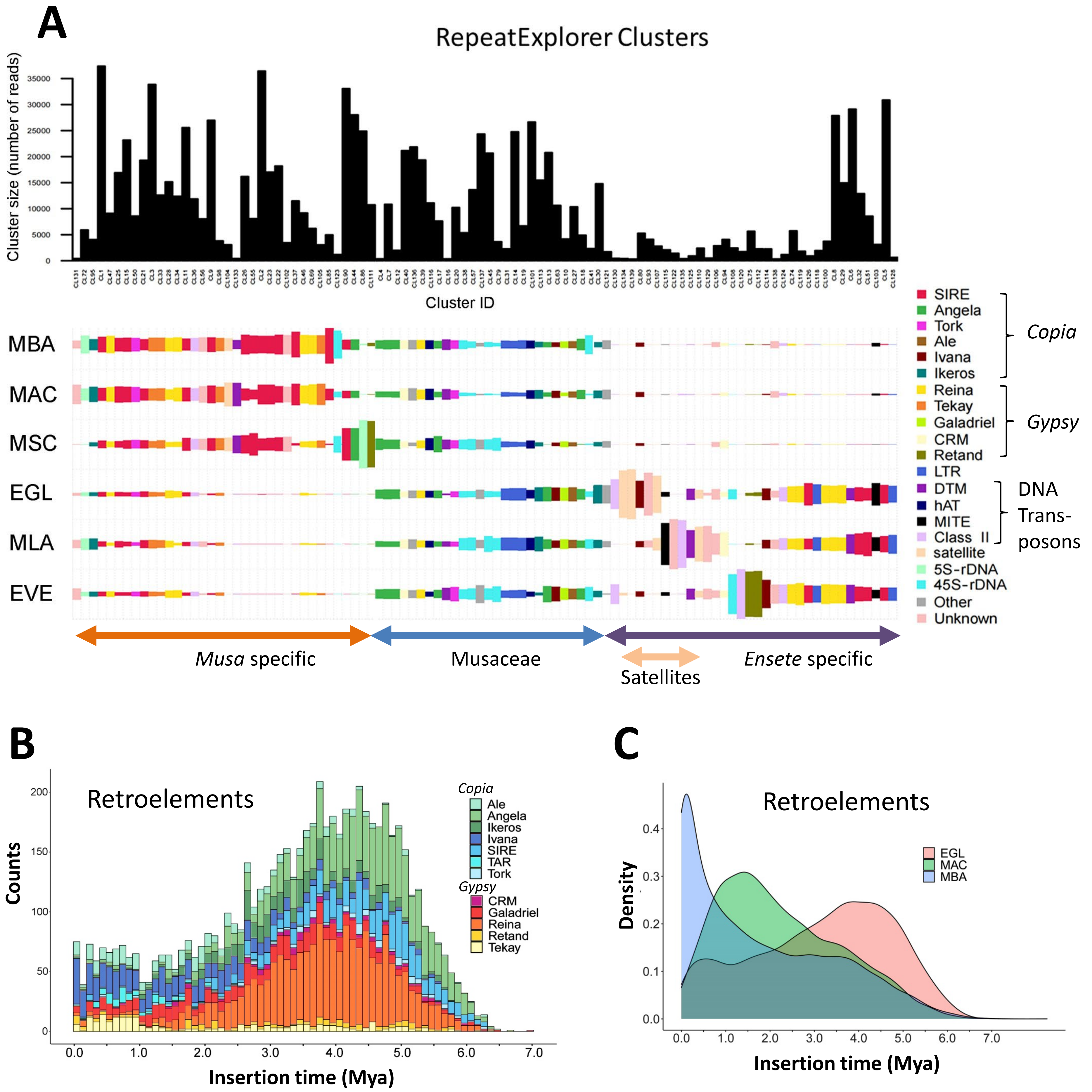

Fig 5 Click here to access/download;Figure;Fig5 Revised 2022-01-24.pdf

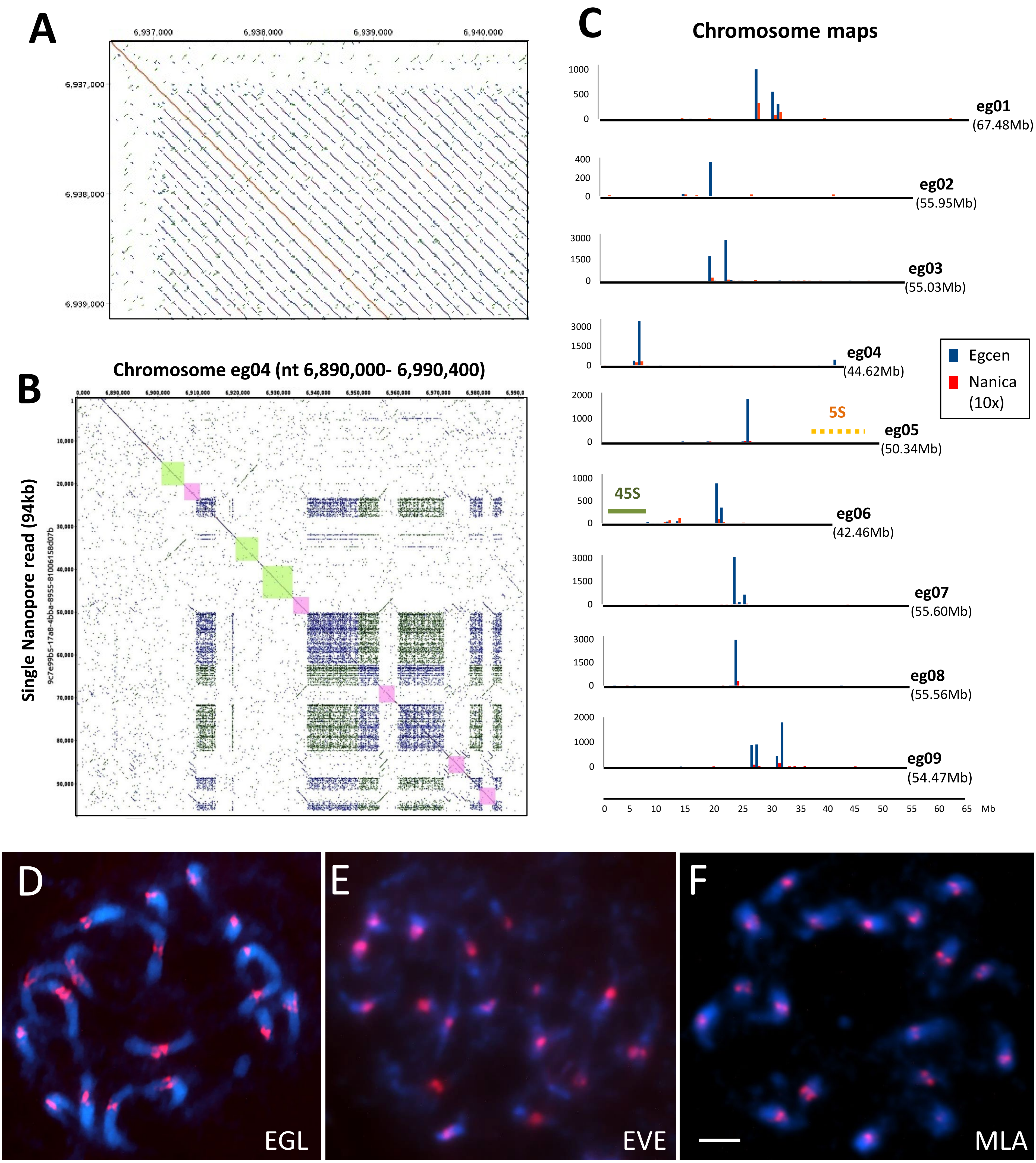

Click here to access/download;Figure;Fig6 Revised 2022-01-24.pdf

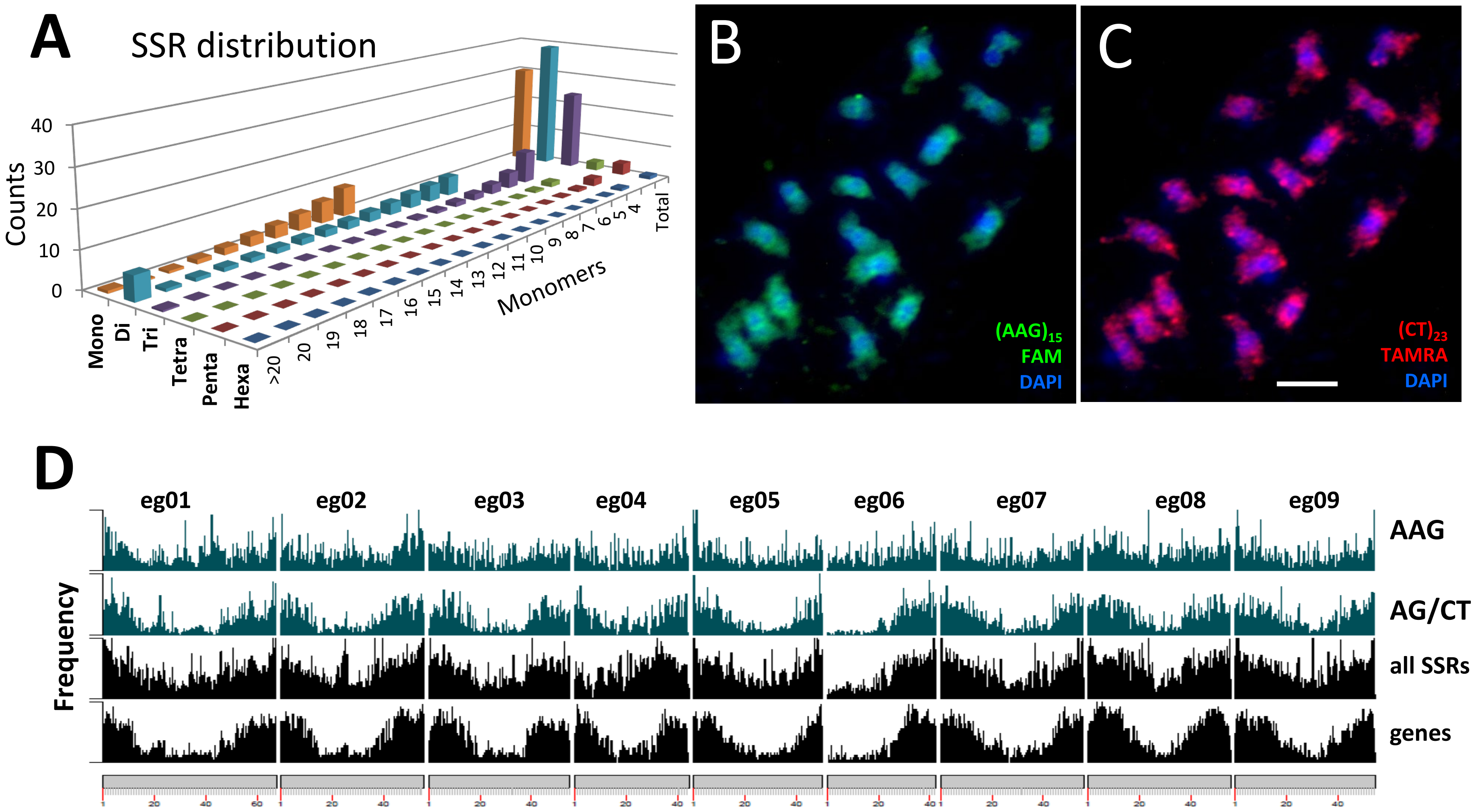

Fig 7

Click here to access/download;Figure;Fig7 Revised 2022-01-24.pdf

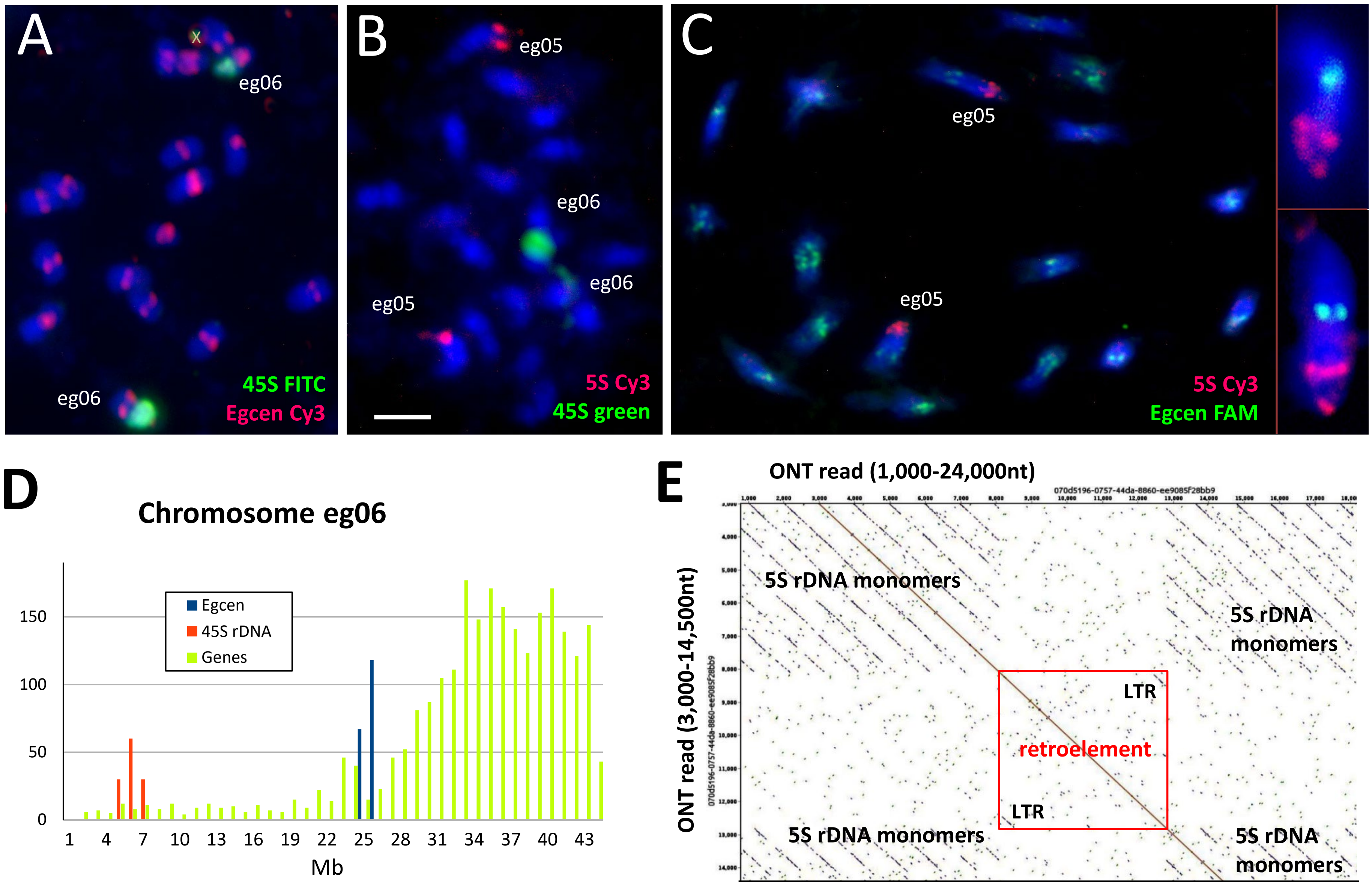

Click here to access/download;Figure;Fig8 Revised 2022-01-24.pdf

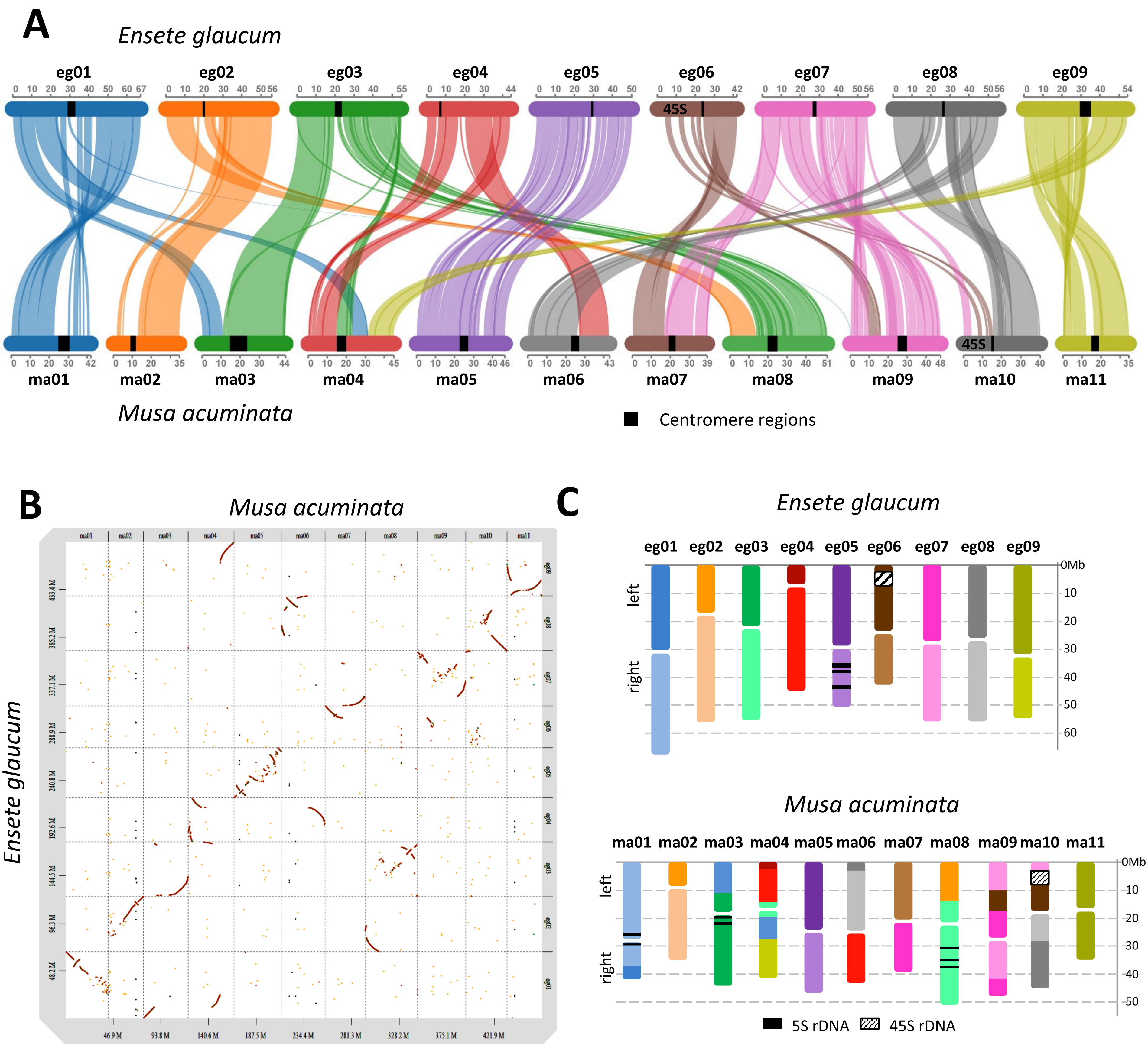

Supplementary figure

Click here to access/download
Supplementary Material
3_Supplementary_ Figures_revised_2022-01-24a.pdf

Supplementary table

Click here to access/download
Supplementary Material
4_SupplementaryTables_supplementary material.xlsx
