# Supplementary material for: A chromosome-level reference genome of Ensete glaucum gives insight into diversity and chromosomal and repetitive sequence evolution in the Musaceae
Source: Gigascience. 2022 Apr 30;11:giac027. doi: 10.1093/gigascience/giac027 (PMC9055855; doi:10.1093/gigascience/giac027)
Supplement: giac027_Response_to_Reviewer_Comments_Original_Submission [file giac027_response_to_reviewer_comments_original_submission.pdf]

Author's Response To Reviewer Comments

Close

26 January 2020
Dear Editor Hongfang Zhang,
We thank the editor and reviewers for their assessment of our manuscript "A chromosome-level reference genome of Ensete glaucum gives insight into diversity, chromosomal and repetitive sequence evolution in the Musaceae" (GIGA-D-21-00354) and the opportunity to prepare a revised manuscript for publication in GigaScience.
We are grateful for the extensive comments which were very valuable for improving our manuscript. We have now addressed all of them as outlined below, and we have carefully revised the manuscript, figures, tables and supplementary data accordingly. A clean version of the manuscript is submitted.
All authors have approved the revised submission and as before declare no conflict of interest.

One referee query whether the manuscript should be a “research article” and not a “data note”; this would remain obviously your editorial decision.

We hope that our revision meets the requirement for publication in GigaScience, and are looking forward to hearing from you in due course.
With regards
Ziwei Wang, Qing Liu, Mathieu Rouard, Trude Schwarzacher and Pat Heslop Harrison (in the name of all authors)
wangziwei@scbg.ac.cn, liuqing@scib.ac.cn, m.rouard@cgiar.org, ts32@le.ac.uk, and phh4@le.ac.uk

Wang et al. "A chromosome-level reference genome of Ensete glaucum gives insight into diversity, chromosomal and repetitive sequence evolution in the Musaceae" (GIGA-D-21-00354)
Response to reviewers’ reports
Reviewers’ comments are copied and our answer follows after each comment.

Reviewer #1: Comments to the authors
Wang et al. generated a chromosome-scale genome sequence assembly of Ensete glaucum based on ONT long reads. This is a valuable resource for comparison against various Musaceae species. This assembly will certainly help to identify genes underlying agronomic traits in Musaceae. Important data sets are already well integrated into the banana genome hub and available to the community. The authors harnessed this highly contiguous assembly for analyses of synteny against Musa acuminata and for the investigation of repeats/TEs. Overall, the quality of this work is high and the manuscript is well written.
Answer: We thank the reviewer for the positive comments.
I am not sure why this submission is classified as a data note, because it could also pass as a research article.
Answer: Our article was originally submitted as a Research Article, but following Editor’s request, we had changed it to a Data Note. We agree with the reviewer as we analysed extensively the assembly and propose some interpretation with regards to the evolution of Musaceae.

I noticed a few issues and provided some specific comments that might be helpful to further improve the quality of this work:
Answer: We thank the referee for the detailed and helpful comments.

1) There are many numbers in the abstract. I would recommend to reduce this to the most important ones. For example, the BUSCO results could be removed.
Answer: We agree with this comment and have deleted the BUSCO value, and length and proportion of centromere satellites in the abstract.

2) There is only one short paragraph about existing genome sequences. I would recommend to extend this and to mention the banana genome hub as the central community resource.

Answer: As suggested, we have extended this section to include detail about the available Musa genome assemblies and the banana genome hub (see p. 5, lines 85-90).

3) Please indicate if the coverage estimations are based on the haploid or diploid genome size (Table 1).
Answer: Genome size is based on haploid measurement, as is the convention but should have been stated. Added now to the Table legend.

4) Please provide additional details about the BUSCO results (C, S, D, F, M) in line114 and/or in Table 2.
Answer: Additional BUSCO details have been added to the text summarizing the data in Table S3. See Page 7; lines 125–127.

5) I find the sentence in line 120/121 confusing when reading for the first time. This suggests to me that more sequence was anchored than present in the initial assembly. The sentence is correct, but it might be better to present the total assembly size first and to describe the anchored proportion in a separate sentence.
Answer: We agree and have changed the sentence to read (Page 7; lines 129-131):
'The contig-level assembly size is 495,175,598 bp, and 97.2% of these contigs are anchored to 9 pseudo-chromosomes after Hi-C scaffolding, resulting in a 481,507,213 bp final genome assembly.'

6) It would be helpful to clearly distinguish between the genome (DNA) and the genome sequence (the assembly). That would make it easier to understand the discussion of differences between both (e.g. collapsed repeats).
Answer: We agree. We have made clear where we mean "assembly" and where we mean genome throughout the manuscript.

7) Genome size estimation is always tricky. I would recommend to run several tools and to provide the estimated range (findGSE, gce, MGSE, GenomeScope, ...). It is also important to run the k-mer-based approaches with different k-mer sizes. Apparently, GenomeScope was used for the heterozygosity analysis, but not for the genome size estimation. That is surprising.
Answer: Thank you for this comment. We fully agree with the reviewer that genome size estimation 'is always tricky' and we faced this situation here. There is discussion of genome size measurements "Comparison of Musa acuminata assemblies" in Belser et al. 2021 with respect to Musa acuminata. We had used several size estimation tools and approaches (with different k-mer sizes) as are conventionally used, and our estimations, and the difference from the assembly and between methods are within the range normally found; our results reveal nothing unusual nor noteworthy. We believe it is best to show results from a similar approach to those published for assemblies of other species, rather than giving extensive comparisons of different methods, given that no one is clearly better than any other. The ancestral genome duplication (evident in the central circle of Fig. 1) influences most methods and means that small changes in parameters change estimates. GenomeScope had been one of several methods of genome size estimation considered.
Following the referee's comment, we have once more generated 17-25mer as input data (see below). We find that findGSE results are quite stable around 588-590 Mb, while GenomeScope results are lower and unstable; it seems as the mer get larger, the estimated size continuously gets larger. The web program version of GenomeScope with k=17, gave a value that was an outlier being below the assembled contig size (as shown in Fig. S1, 468,990,370bp). We also tried MGSE, and the coverage estimates based on mean and median (see below) span the genome size estimates using k-mers.
After careful reconsideration, we believe as before that an estimated haploid genome size of 563,295,571bp as we had reported in Table 2 is the most appropriate estimate. We had based this estimate on the 17-mer peak frequency of Illumina DNA sequencing (with the formula k-num/k-depth where k-num is the total number of 17-mers, 30,417,960,841; and k-depth the highest k-mer depth, 54; see Materials, Methods and Validation section) rather than using findGSE or GenomeScope software. It is also consistent with the gene coverage in raw reads analysed by MGSE as suggested by the referee. The section in the MS has been updated; see page 7, lines 136-142.

Genome size estimation using different software programs
findGSE:
Genome size estimate for jellyfish.all.findGSE.17.kmers.fa.histo: 588760154 bp.
Genome size estimate for jellyfish.all.findGSE.18.kmers.fa.histo: 581881350 bp.
Genome size estimate for jellyfish.all.findGSE.19.kmers.fa.histo: 585421172 bp.
Genome size estimate for jellyfish.all.findGSE.20.kmers.fa.histo: 588031514 bp.
Genome size estimate for jellyfish.all.findGSE.21.kmers.fa.histo: 588939614 bp.
Genome size estimate for jellyfish.all.findGSE.22.kmers.fa.histo: 590416567 bp.

Genome size estimate for jellyfish.all.findGSE.23.kmers.fa.histo: 590204947 bp.
Genome size estimate for jellyfish.all.findGSE.24.kmers.fa.histo: 590395417 bp.
Genome size estimate for jellyfish.all.findGSE.25.kmers.fa.histo: 590573612 bp.

GenomeScope run on local computer:
GenomeScope analyzing jellyfish.all.17.kmers.fa.histo p=2 k=17 outdir=17
Model converged het:0.115 kcov:55.7 err:0.00236 model fit:1.09 len:242980381
GenomeScope analyzing jellyfish.all.18.kmers.fa.histo p=2 k=18 outdir=18
Model converged het:0.00182 kcov:27.6 err:0.0017 model fit:0.984 len:497383705
GenomeScope analyzing jellyfish.all.19.kmers.fa.histo p=2 k=19 outdir=19
Model converged het:0.00184 kcov:27.3 err:0.00173 model fit:0.97 len:502344401
GenomeScope analyzing jellyfish.all.20.kmers.fa.histo p=2 k=20 outdir=20
Model converged het:0.00176 kcov:27 err:0.00172 model fit:0.964 len:506689085
GenomeScope analyzing jellyfish.all.21.kmers.fa.histo p=2 k=21 outdir=21
Model converged het:0.00172 kcov:26.8 err:0.00171 model fit:0.958 len:510593088
GenomeScope analyzing jellyfish.all.22.kmers.fa.histo p=2 k=22 outdir=22
Model converged het:0.00167 kcov:26.5 err:0.00168 model fit:0.952 len:514042691
GenomeScope analyzing jellyfish.all.23.kmers.fa.histo p=2 k=23 outdir=23
Model converged het:0.00158 kcov:26.3 err:0.00166 model fit:0.949 len:517304358
GenomeScope analyzing jellyfish.all.24.kmers.fa.histo p=2 k=24 outdir=24
Model converged het:0.00153 kcov:26.1 err:0.00163 model fit:0.945 len:520395633
GenomeScope analyzing jellyfish.all.25.kmers.fa.histo p=2 k=25 outdir=25
Model converged het:0.00148 kcov:25.8 err:0.0016 model fit:0.94 len:523313733

GenomeScope run on server:
468990370 bp for web-version of GenomeScope k=21 Figure S1
407601233 bp for web-version of GenomeScope k=17 het 0,.156

MGSE based on BUSCO gene site coverage:
" processing: result/EGL.NGS.sort.bam.cov
average coverage in reference regions (mean): 62.9484059496
average coverage in reference regions (median): 75.0"

With the mean coverage as 62.95x and the median as 75x, and 36.878GB of Illumina sequence, this represents 585,830,000 bp or 491,797,000 bp genome sizes, consistent with the k-mer estimate but perhaps distorted by the ancient whole genome duplication, heterozygous genes and recent duplications.

8) Statistics about the pseudochromosomes in Table 2 could be removed. For example, it is not necessary to say that the L50 number of 9 chromosomes is 5.
Answer: We have removed the N50 and L50 lines in Table 2. We had previously given them to facilitate numeric comparisons with published values for less complete assemblies.

9) Please explain the difference in BUSCO results between predicted genes and BUSCO run in genome mode. Which genes are missing in the annotation? Table S3 suggests that the automatic BUSCO annotation (genome mode) is superior to the annotation generated in this study (analyzed in transcriptome mode).
Answer: The two BUSCO results were used to evaluate different aspects. The "genome" mode in BUSCO was to assess the genome assembly completeness; it uses assembly Fasta files as input and it de novo searches the BUSCO genes in the assembly. The transcriptome mode here, on the other hand, was to evaluate our gene annotation quality; we used CDS translated from predicted genes as input, so they are relatively independent. The genome mode BUSCO is commonly superior to the annotation transcriptome mode (see e.g. https://doi.org/10.1016/j.cell.2020.09.043; Table 2).

10) Some statements about the CENs and telomeres would be interesting. These could give a good impression of the assembly results. Estimating their copy numbers could help to explain the difference between assembly size and estimated genome size.
Answer: There are lengthy statements about CENs under the heading Tandem (satellite) repeats and centromeric sequences (seeWe did not analyse the assemblies at telomeres; as with the head-to-head junctions (see point 17), we found problems with the ONT technology, now reported by Tan et al. 12 Jan 2022 (Identifying and correcting repeat calling errors in nanopore sequencing of telomeres BioRXiv https://doi.org/10.1101/2022.01.11.475254). So, with also the cell-to-cell variation, full-length telomere assemblies could not be made. There were some short telomere sequences within a few ONT

reads, both mapped internally and terminally on the chromosome assemblies, and these sequences are deposited). We have added telomeres, along with the rDNA, into the comment about the difference between assembly lengths see Page 7; lines 131-132.

11) Are there any genetic markers that could be used to check the assembly accuracy?
Answer: There are extensive publications showing various types of DNA-based markers in Musa and Ensete ventricosum, but not E. glaucum. Both the high level of synteny observed and presence of genes in the BUSCO analysis suggests that many SNP-based markers will work, and provides a check of assembly and its accuracy.
The synvisio and dotplot analyses in Fig. 8 further prove extensive synteny between Musa acuminata and E. glaucum. The SSR analysis in Fig. 6 and Table S14 indicates the conservation of frequency of SSR motifs (in combination with our previous analysis of SSR in Musa and E. ventricosum, Biswas et al. 2019). There is no genetic map for any Ensete species to check marker or sequence ordering, but the Hi-C contact matrix (Figure S12) gives another independent check of assembly accuracy. Given our high-quality sequence, future work is more likely to use survey sequencing rather than more limited and laborious genome-wide marker surveys.

12) In my opinion, the section "Gene distribution and whole-genome duplication analysis" could be removed. Genes are never equally distributed across a genome and repeats/TEs are usually clustered around the centromeres. Therefore, this part does not add any novel insights. The second paragraph comes to the conclusion that all Musaceae share the same WGDs. This seems obvious to me. Was there a different expectation?
Answer: We have deleted the heading and merged it with the previous section now called 'Genome size, heterozygosity and organization'. We agree there are no novelties here, but it is important to cross-check and document known (expected) results and show these are seen, before we contrast features that differ between the species (see also point 18). Ensete is unique (unexpected) with the centromeric repeat and different LTR retrotransposons as emphasised later.

13) Orthogroup identification could be complemented with a synteny analysis. A comparison to Musa acuminata (https://doi.org/10.1038/s42003-021-02559-3) could help to check the accuracy of the orthogroups.
Answer: Thank you for the suggestion. Orthogroup identification using OrthoFinder (and MCL more generally) is a robust method that have been used extensively in literature, including GreenPhylDB by Guignon et al, 2021(shared authors with our paper). So, we trusted the accuracy and direct comparability to published analyses of results for subsequent analyses. Combining MCL based approaches and microsynteny is indeed a promising strategy for accuracy, although to our knowledge popular tools or pipeline doing both is not that easy to apply.

14) The statement "Genes with Ka/Ks > 1 were under positive selection (Supplementary Table S6)." does not fit well to the rest of this paragraph. Given that there are >35k genes, some would show values >1 by chance. Some statistical test would be needed to find out which genes are actually under positive selection. What is the conclusion from the identification of such genes? Any enrichment of particular functions?
Answer: This is a good point, but analysing which genes are there by chance or due to biology is a lengthy analysis. We have added an additional figure (Figure S2A) to show GO enriched terms of positively selective genes. These summarise the extensive enrichment of biological regulatory processes. We have added this fact in the main manuscript and point out the novel features. see page 10, lines203).Further analysis will be required to examine the details and consequences of genes under positive selection and their enrichment, which are not the focus of this manuscript.

15) The statement about the sugar transporters is interesting. This would be a good chance to connect these comparative genomics results with the transcriptome analyses.
Answer: Again, we agree with the comment, but as a genome-wide and structural analysis of the genome, study of individual gene groups and pathways is beyond the scope of this manuscript.

16) Transcription factor families are mentioned, but not discussed. It is not surprising that MYBs are the largest TF gene family. However, it would be interesting to know if there are any striking differences compared to M. acuminata (https://doi.org/10.1371/journal.pone.0239275). Some MYBs like the anthocyanin regulators respond to sugar treatments. Is there a connection to the large number of sugar transporters? Any duplications/deletions compared to M. acuminata? This could be another opportunity to better connect different aspects of this study.
Answer: Transcription family analysis in a comparative context is certainly important and something

we work on (e.g. Cenci A, Rouard M. Evolutionary analyses of GRAS transcription factors in angiosperms. Frontiers in Plant Science. 2017 Mar 2;8:273) and will be studied further in more extensive work, but would be beyond the scope of the current paper.
17) It is interesting to read that head-to-head and tail-to-tail repeats appeared collapsed. Previous studies identified that these arrangements of repeats are associated with low local read quality (e.g. https://doi.org/10.1093/nar/gkaa206, https://doi.org/10.1186/s12864-021-07877-8). I would not expect that both strands of the DNA molecules are sequenced. The authors might want to check this and provide additional explanation.
Answer: We agree we "would not expect", but there is discussion (unpublished, uncitable and not archived) on the ONT/Nanopore user forum about the phenomenon of a read including a substantial reverse-complement fragment. As suggested, we do see a rapid (although limited) change in read quality between the forward- and reverse-complement components of a single read (giving some support to the artefactual nature). We feel it is important to indicate that caution is required in analysis but this is not the place for a detailed study of ONT technology and the base-calling software.

18) I am surprised that TEs were the most abundant class of repeats. Could this be caused by treating at all the different TEs as one group? CENs should appear with a much higher copy number than individual TEs or TE families.
Answer: We are not surprised at the high proportion of TEs which is largely as expected from Musa and most other species. The differences in abundance between classes in Musaceae is unexpected. Many assemblies collapse the TEs so they may be hugely under-represented in the assembly but not in the reads; see comparison of Musa acuminata in Belser et al. 2021 with 246 Mbp of the genome (52.6%) as TEs in V4, compared to 152 Mbp in V2. The length (and genome proportion) of CENs, typically several megabases, far lower than the proportion of retroelements in the genome, is typical of many species.

19) The centromeric patterns could be compared to the situation in Arabidopsis thaliana: https://www.science.org/doi/10.1126/science.abi7489.
Answer: We think it is important to analyse, with the contrast in presence or absence of a centromeric tandem repeat (cf. human vs African Green Monkey). We had already carried out the first analysis of the Arabidopsis centromeric repeat (Heslop-Harrison et al 1999, now confirmed; and cited in the present manuscript with respect to the CENP-B box) but we think a theoretical bioinformatic analysis to compare Ensete and Arabidopsis would need to be complemented by CENH3, ChIP and methylation analysis. It will also need to be in a comparative context of our Musaceae and Arabidopsis (including the former Cardaminopsis) studies of centromeric tandem repeats, and include recent findings in wheat, rice and maize. Again we feel that this is beyond the scope of the present paper.

20) Are SSR less frequent around the centromeres and on the NOR chromosome arm or is this just a lack of detection in these regions?
Answer: SSRs are less common around centromeres and the NOR arm; we used both genome sequence analysis (informatics) and chromosomal in situ hybridization (cytology) (Figure 6B, C): they are independent methods showing the same distribution.

21) Why is AG/CT more abundant than other SSRs? This could be compared to other species.
Answer: We have recently discussed this issue, including comparison with Ensete ventricosum (first pass Illumina assembly only), Musa and many other species (Biswas et al. 2019 - their supplementary figure S2). The extension to Ensete glaucum is made in Fig. 6 and Table S14 and we have now extended the discussion. Page 18; Lines 374-378.

22) References for the length of 45S rDNA length in other species are missing.
Answer: We added three comparator references. However, neither number of copies nor full-length assemblies have been made in many other species with genome assemblies using a directly comparable and accurate unselected read-mapping approach. Analysis of genome assemblies, and particularly programs such as RepeatMasker, give wrong results. Hence addition of comparators using rigorous methods requires extensive (although straightforward) analysis of large amounts of sequence data from other species, beyond the scope of our Musaceae work.

23) How many 45S rDNA copies can be inferred from the ONT reads. The coverage is way higher thus this estimation should be more reliable.
Answer: The coverage of Illumina reads is more than adequate to give an accurate estimate and does not change significantly whether 10% or 100% of the data are used. The challenge with ONT reads is determining the number of copies in each read, but the result is consistent with Illumina read analysis.

24) NOR chromosome arm is depleted of protein encoding genes, but there should be plenty of rRNA genes. Please specify this in the sentence.
Answer: Phrasing is corrected to say protein-coding genes are depleted in the NOR arm as figure 7D shows. The tandemly repeated rRNA genes are restricted to a short chromosomal region of this arm (about 3-4Mb of 24Mb, although somewhat collapsed in the assembly) and it is notable that the remainder (non rDNA part) of the arm is also depleted of genes.
The rDNA monomer is about 10 kb in length and includes transcribed and non-transcribed spacers. rDNA units are interspersed by other repeats (tandem repeats and retroelements) and hence leave little space for other genes. From Figure 2 (circos plots), we also see that the remaining arm is very rich in repeats corresponding to the low gene density; and the synteny analysis of Fig 8A shows the same low gene density for the Musa acuminata NOR arm (compare eg06 and ma10). We can speculate why this is, but we feel that stating the fact is all we can do at this point.

25) The synteny section is lengthy. The statements in context of previous studies are good, but removing some purely descriptive parts might make it more interesting. The corresponding figures show everything and could stand on their own.
Answer: Chromosome structural evolution is the most important, novel, and rigorous analysis which the whole-chromosome assembly allows us, for the first time, to carry out. There are very few equivalents for any plant or animal, and it is therefore emphasized. We feel the entire narrative (results and discussion) is needed for the benefit of the reader.
26) What is the value of genotyping-by-sequencing if not combined with GWAS?
Answer: Genotyping-by-sequencing has been useful to conduct intraspecific diversity analyses and was also proven as an efficient way to study chromosome structure of banana cultivars as done in Cenci et al. (shared authors with our paper) in https://doi.org/10.1093/aob/mcaa032

27) Which ONT flow cell type? Which Guppy version?
Answer: The flow cell version was R9.4.1 and the guppy version was 2.0.8. We have added this information to manuscript. (see page26, line 568).
28) It does not become clear how the Hi-C library was prepared (line 562). What is the improvement? Please explain this here.
Answer: We added the detail. (see page 26; lines 576-587).

29) Please add the detailed parameters of the assembly and polishing.
Answer: More details are added in the manuscript in the relevant M&M sections about the assembly. (see pages 27 and 28)

30) BWA reference is missing. Why was BWA not used for the mapping of the Hi-C reads?
Answer: Thank you for noticing; we have added the reference. We used bowtie2 for mapping the Hi-C reads rather than BWA because bowtie2 is the default mapper of HiC-pro.

31) The statement in line 592/593 suggests that Hi-C was used for validation. However, it was also used for correction in the previous step. Anyways, this result should be moved from the method to the result section.
Answer: As stated, the Hi-C and ONT data were used for the primary assembly; we do not state Hi-C was used for 'validation' as that would be a circular argument (in contrast, other published assemblies use Hi-C after assembly for validation and correction). However, the Hi-C contact map is shown in the Materials and Methods as its generation was a key point of the Methods, and it can be usefully compared with other publications.
32) Trinity assembly and PASA steps lack details.
Answer: More details are added in the manuscript. (see page 28; lines 625-628)

33) Parameters of STAR mapping and gene prediction steps are missing.
Answer: we have now added the information. Software programs where we used specific parameters, we have added these; the rest were used with default settings.

34) There is some discrepancy concerning the Musa acuminata genome assembly versions. It seems that v2 is used in some cases and v4 in others. Please check this.
Answer: All the analyses were performed initially with the released version 2 of Musa acuminata 'DH Pahang'. Late in the preparation of the manuscript the version 4 was published (involving common co-authors), and we felt that this new resource could be more appropriate for the analyses of chromosome structure as the location of centromeric regions was improved. Therefore, we used V4 for these analysis (represented in Figure 8) and made it clear in the manuscript in the material and methods/Synteny

section (see page 32; lines 708-711) . For the other analyses, each time the version 2 is appropriately indicated.

35) Please make the customized script available via github (line 732) if this is different from the one mentioned in line 737.
Answer: The script was available on github at https://github.com/wangziwei08/LTR-insertion-time-estimation which gives more details. There was an issue with the link in reference 114, which is fixed now.

36) Are the TE results consistent if a different 2Gb subsets of the illumina data are analyzed?
Answer: Good question, but YES, results are consistent. Actually, we used several subsets, and smaller sets with this species and others, and the results do not change within one set of reads once more than c. 1Gb of data are used (also evident from the RepeatExplorer papers of Neuman, Macas et al.).

37) How were the centromere positions determined? I think that I have missed that in the method section. It must be connected to the CEN repeats, but the precise approach could be explained in more detail.
Answer: The centromere region is unequivocally identified by the presence of the repeat Egcen that localises to all chromosomes by FISH at the cytological primary constrictions that are seen as gaps by DAPI staining; this has been described in the result section and Figure 5D-F legend.
We used BLAST of the Egcen consensus to the assemblies, noting the array positions and represented results as bars on graphs for each chromosome in Figure 5B. Single or double arrays correspond to the chromosomal FISH picture (Figure 5D-F). For calculating an inferred centromere mid position (Table S13) we used the start and end of the Egcen arrays and calculated the midpoint for each array with the mean if there are two arrays assuming one is on the right and one on the left of the centromere region. In Figure 8A, a large bar represents the centromere region and in Figure 8B a gap is drawn to indicate that the centromere is not at one particular nucleotide.
This is now explained in the Figure legends to Fig 5 and Table S13 in more detail.

38) The read data sets are not released thus I cannot check if all raw data sets were included. It would be particularly important to have the FAST5 files of the ONT data to study base modifications in the future.
Answer: We agree strongly about having access to FAST5 files (and indeed have asked authors of other papers for these data but most have deleted them). The possibility of upload of FAST5 raw data to NCBI is a new capability but currently the function is not working correctly. The files are available on hard disk by mail, and will be uploaded when the function is available. The raw read dataset of ONT, NGS, Hi-C and RNA have released on NCBI, under BioProject: PRJNA736572 (SRR15039764 - SRR15039770).
39) The link to the banana genome hub appears to be broken in the data availability statement. The data sets on the genome hub look fine.
Answer: Thank you for pointing out this issue. There was a typo with the hyperlink that is now fixed to redirect to a dedicated page to download the datasets. http://banana-genome-hub.southgreen.fr/

40) The terms "core" and "pseudo-core" in Fig. 3 are not frequently used in the literature. These genes seem to have different degrees of dispensability and might be conditionally dispensable (https://pubmed.ncbi.nlm.nih.gov/24548794/; https://doi.org/10.1186/s13007-021-00718-5).
Answer: We agree with the reviewer that this can be a tricky concept. The pseudo-core concept was used to describe a set of genes similar to what is defined as softcore in the Brachypodium pangenome by Gordon et al, 2017 https://doi.org/10.1038/s41467-017-02292-8. The term is intended to highlight the uncertainty with the number of genes and the challenge of assembling and annotating a particular gene model correctly in every sequenced genome. We changed it to softcore in the manuscript wherever it applied.

41) There seems to be some variation in the genome size estimation. I would recommend to present the results of multiple k-mer sizes (e.g. 17-25). The distribution of the resulting values might help to estimate the true genome size.
Answer: Please see our answer under point 7.

42) The presented sugar transporters are not among the top enriched GO terms (S2). Therefore, I am afraid that this analysis is not very informative. Could it be that the "enriched" GOs are just a "random" set?
Answer: Carbohydrate metabolism is an important feature of Musaceae but the extensive and detailed

analysis of the range of genes, including validation of GO term enrichment, is beyond the scope of the present genome assembly work (see also point 14). Fig. S2 is now revised.

43) Why is E. glaucum not presented as S5C? A direct comparison would make more sense.
Answer: E. glaucum result is now added as Fig S5C; it was not presented in Fig S5 before because it is resented in figure 4B, albeit with a different colour code.

44) S10: I would recommend to identify the precise break points. Next, it would be good to validate the accuracy of the assembly by finding individual reads that actually support the situation in E. glaucum. This would help to exclude an assembly artifact as reason for the difference.
Answer: Figure S10 was a contraction of a much more extensive figure where individual ONT reads were mapped to the exact translocation breakpoints. As with Fig. 5B, ONT reads were able to validate the assembly. The Hi-C contact plot of Fig. S12 also independently validates the accuracy of the assembly since discontinuities in the major diagonal would be evident. We have extended Figure S9 to add (D) the M. balbisiana to mb05 to eg05 dotplot showing the single inversion, and S10B to include some ONT reads spanning the breakpoint. The breakpoint of inversion between E. glaucum and M. balbisiana is after bp 4,478,642 (Fig. S10B) and 7,669,296.

45) It might be better to use a three letter abbreviation of the species ("Egl" instead of "Eg") in the gene IDs to avoid ambiguities in future genome sequencing projects.
Answer: We agree with the reviewer's remark that a 3-letter nomenclature for locus tags and chromosome names would have been more appropriate. It is not practical now to change everything as data are already online for users in the browser (banana-genome-hub) Possibly, if there is a new version of the assembly or gene annotation, it might be an opportunity to apply it.

46) The method section states that short DNA fragments below 12kb were removed. S11 suggests that two libraries were sequences: one with depletion of the short fragments and one without it. Please check this. Generally, I would recommend to try a different gDNA extraction protocol and to use SRE instead of BluePippin.
Answer: We thank the reviewer for the suggestion. We aimed to remove DNA fragments below 12kb, using two approaches. Some DNA molecules may get fragmented during sequencing or entering the sequencer. Fig. S11 shows the high proportion of ONT reads >20kbp for assembly, and we had 229x coverage which is enormously high (even if some reads are shorter – allowing for their removal). Musaceae DNA extraction is difficult: numerous publications in the 1990s and early 2000s discuss reasons, providing "improved" or "optimized" protocols. The DNA isolation approaches we used were already optimized and gave sufficient long-molecule DNA for long read sequencing.

47) The north of eg06 looks suspicious in the Hi-C analysis (S12). There is also no substantial synteny with any of the Musa chromosomes (S8). Could this be an indication that there are errors in the assembly?
Answer: We discuss the interesting picture with eg06, which is likely to be entirely caused by the low protein-coding gene density, presence of the NOR/45S rDNA tandem repeats, and other repeats (see also answer to point 24). The lack of protein-coding genes in this region of eg06 means no substantial syntenic relationships are shown - synteny need syntenic genes.

48) Table S1: What is the point in showing that all contigs are larger than 1, 2, and 5kb?
Answer: This was included for easy and direct comparison with tables in previous publications, although, some like L50, are of no meaning with a highly contiguous sequence. We have deleted these lines now.

49) 445 bHLHs in M. acuminata is almost twice the number of bHLHs detected in E. glaucum. Some other TF families also show this large difference, but other families show almost equal numbers. It could be interesting to further investigate this. The HB-KNOX value of M. acuminata is missing.
Answer: We agree that it would be interesting to analyse the differences in the bHLH family (including additional manual curation), and this will be studied in future more extensive work. See also point 16 regarding transcription factors.

Minor comments:

line 70/71: Some countries are named multiple times. Please change this.
Answer: Done

line 113: chromosomes > pseudochromosomes
Answer: Done

line273/274: Please check this sentence.
Answer: we corrected the sentence to read: “In E. glaucum, both Copia and Gypsy families show relatively constant activity over the last 2.5 My, with further major peaks of insertion activity at 3.5 to 5.5 Mya, (Fig. 4B, C) corresponding to the half-life of LTR- elements [14]”

line 428: Please rephrase "translated proteins" and SynVisio should only be named in the method section.
Answer: agreed and done.
line 436: "protein-coding genomes" ?
Answer: corrected into protein-coding genes.

line 464: "second (right)" ... should be replaced by north/south or q/p nomenclature. This also affects some following sentences.
Answer: With largely sub-metacentric chromosomes (and small differences between species ‘reversing’ arms), we do not think the designation p/q is appropriate (and N/S also indicates the relative sizes with N conventionally the short p arm). The key figures also show horizontal chromosomes with left and right arms as they were assembled.

line 625: "Musa acuminata" is a species name
Answer: Done

line 639: blast > BLAST
Answer: Done

line 731: of of > of
Answer: Done

line 811: RNA-sequencing > RNA-seq (I have not seen a section about RNA sequencing)
Answer: RNA-seq were generated for the purpose to increase the gene annotation and not for gene expression studies. It is hence mentioned in M&M and not discussed in detail in the result/discussion section.

S10: "E glaucum" > "E. glaucum"
Answer: This mistake in Figure S10 has now been corrected.

Reviewer #2: Comments to the authors

In this study, the authors described the generation of a high-quality reference genome of Ensete glaucum, which is one of the most cold-hardy species in the Musaceae. It is also well known for its drought tolerance. The authors compared the expansion and contraction of gene families and the composition of repeats among related species. The genome assembly, analysis, and annotation are certainly useful for comparative genomic studies as well as future breeding practice. Everything seems to make sense to me. Certainly, the results are descriptive, but this is more than sufficient for a data note.

Answer: We thank the referee for the very positive comments.

Close
