# Supplementary material for: A chromosome-level reference genome of Ensete glaucum gives insight into diversity and chromosomal and repetitive sequence evolution in the Musaceae
Source: Gigascience. 2022 Apr 30;11:giac027. doi: 10.1093/gigascience/giac027 (PMC9055855; doi:10.1093/gigascience/giac027)
Supplement: giac027_Reviewer_1_Report_Original_Submission — Boas Pucker -- 11/27/2021 Reviewed [file giac027_reviewer_1_report_original_submission.pdf]

Reviewer Report

Title: A chromosome-level reference genome of Ensete glaucum gives insight into diversity, chromosomal and repetitive sequence evolution in the Musaceae

Version: Original Submission **Date:** 11/27/2021

Reviewer name: Boas Pucker

Reviewer Comments to Author:

Wang et al. generated a chromosome-scale genome sequence assembly of Ensete glaucum based on ONT long reads. This is a valuable resource for comparison against various Musaceae species. This assembly will certainly help to identify genes underlying agronomic traits in Musaceae. Important data sets are already well integrated into the banana genome hub and available to the community. The authors harnessed this highly contiguous assembly for analyses of synteny against Musa acuminata and for the investigation of repeats/TEs. Overall, the quality of this work is high and the manuscript is well written. I am not sure why this submission is classified as a data note, because it could also pass as a research article.

I noticed a few issues and provided some specific comments that might be helpful to further improve the quality of this work:

1) There are many numbers in the abstract. I would recommend to reduce this to the most important ones. For example, the BUSCO results could be removed.

2) There is only one short paragraph about existing genome sequences. I would recommend to extend this and to mention the banana genome hub as the central community resource.

3) Please indicate if the coverage estimations are based on the haploid or diploid genome size (Table 1).

4) Please provide additional details about the BUSCO results (C, S, D, F, M) in line114 and/or in Table 2.

5) I find the sentence in line 120/121 confusing when reading for the first time. This suggests to me that more sequence was anchored than present in the initial assembly. The sentence is correct, but it might be better to present the total assembly size first and to describe the anchored proportion in a separate sentence.

6) It would be helpful to clearly distinguish between the genome (DNA) and the genome sequence (the assembly). That would make it easier to understand the discussion of differences between both (e.g. collapsed repeats).

7) Genome size estimation is always tricky. I would recommend to run several tools and to provide the estimated range (findGSE, gce, MGSE, GenomeScope, ...). It is also important to run the k-mer-based approaches with different k-mer sizes. Apparently, GenomeScope was used for the heterozygosity analysis, but not for the genome size estimation. That is surprising.

8) Statistics about the pseudochromosomes in Table 2 could be removed. For example, it is not necessary to say that the L50 number of 9 chromosomes is 5.

9) Please explain the difference in BUSCO results between predicted genes and BUSCO run in genome mode. Which genes are missing in the annotation? Table S3 suggests that the automatic BUSCO

annotation (genome mode) is superior to the annotation generated in this study (analyzed in transcriptome mode).

10) Some statements about the CENs and telomeres would be interesting. These could give a good impression of the assembly results. Estimating their copy numbers could help to explain the difference between assembly size and estimated genome size.

11) Are there any genetic markers that could be used to check the assembly accuracy?

12) In my opinion, the section "Gene distribution and whole-genome duplication analysis" could be removed. Genes are never equally distributed across a genome and repeats/TEs are usually clustered around the centromeres. Therefore, this part does not add any novel insights. The second paragraph comes to the conclusion that all Musaceae share the same WGDs. This seems obvious to me. Was there a different expectation?

13) Orthogroup identification could be complemented with a synteny analysis. A comparison to Musa acuminata (https://doi.org/10.1038/s42003-021-02559-3) could help to check the accuracy of the orthogroups.

14) The statement "Genes with Ka/Ks > 1 were under positive selection (Supplementary Table S6)." does not fit well to the rest of this paragraph. Given that there are >35k genes, some would show values >1 by chance. Some statistical test would be needed to find out which genes are actually under positive selection. What is the conclusion from the identification of such genes? Any enrichment of particular functions?

15) The statement about the sugar transporters is interesting. This would be a good chance to connect these comparative genomics results with the transcriptome analyses.

16) Transcription factor families are mentioned, but not discussed. It is not surprising that MYBs are the largest TF gene family. However, it would be interesting to know if there are any striking differences compared to M. acuminata (https://doi.org/10.1371/journal.pone.0239275). Some MYBs like the anthocyanin regulators respond to sugar treatments. Is there a connection to the large number of sugar transporters? Any duplications/deletions compared to M. acuminata? This could be another opportunity to better connect different aspects of this study.

17) It is interesting to read that head-to-head and tail-to-tail repeats appeared collapsed. Previous studies identified that these arrangements of repeats are associated with low local read quality (e.g. https://doi.org/10.1093/nar/gkaa206, https://doi.org/10.1186/s12864-021-07877-8). I would not expect that both strands of the DNA molecules are sequenced. The authors might want to check this and provide additional explanation.

18) I am surprised that TEs were the most abundant class of repeats. Could this be caused by treating at all the different TEs as one group? CENs should appear with a much higher copy number than individual TEs or TE families.

19) The centromeric patterns could be compared to the situation in Arabidopsis thaliana: https://www.science.org/doi/10.1126/science.abi7489.

20) Are SSR less frequent around the centromeres and on the NOR chromosome arm or is this just a lack of detection in these regions?

21) Why is AG/CT more abundant than other SSRs? This could be compared to other species.

22) References for the length of 45S rDNA length in other species are missing.

23) How many 45S rDNA copies can be inferred from the ONT reads. The coverage is way higher thus

this estimation should be more reliable.
24) NOR chromosome arm is depleted of protein encoding genes, but there should be plenty of rRNA genes. Please specify this in the sentence.
25) The synteny section is lengthy. The statements in context of previous studies are good, but removing some purely descriptive parts might make it more interesting. The corresponding figures show everything and could stand on their own.
26) What is the value of genotyping-by-sequencing if not combined with GWAS?
27) Which ONT flow cell type? Which Guppy version?
28) It does not become clear how the Hi-C library was prepared (line 562). What is the improvement? Please explain this here.
29) Please add the detailed parameters of the assembly and polishing.
30) BWA reference is missing. Why was BWA not used for the mapping of the Hi-C reads?
31) The statement in line 592/593 suggests that Hi-C was used for validation. However, it was also used for correction in the previous step. Anyways, this result should be moved from the method to the result section.
32) Trinity assembly and PASA steps lack details.
33) Parameters of STAR mapping and gene prediction steps are missing.
34) There is some discrepancy concerning the Musa acuminata genome assembly versions. It seems that v2 is used in some cases and v4 in others. Please check this.
35) Please make the customized script available via github (line 732) if this is different from the one mentioned in line 737.
36) Are the TE results consistent if a different 2Gb subsets of the illumina data are analyzed?
37) How were the centromere positions determined? I think that I have missed that in the method section. It must be connected to the CEN repeats, but the precise approach could be explained in more detail.
38) The read data sets are not released thus I cannot check if all raw data sets were included. It would be particularly important to have the FAST5 files of the ONT data to study base modifications in the future.
39) The link to the banana genome hub appears to be broken in the data availability statement. The data sets on the genome hub look fine.
40) The terms "core" and "pseudo-core" in Fig. 3 are not frequently used in the literature. These genes seem to have different degrees of dispensability and might be conditionally dispensable (https://pubmed.ncbi.nlm.nih.gov/24548794/; https://doi.org/10.1186/s13007-021-00718-5).
41) There seems to be some variation in the genome size estimation. I would recommend to present the results of multiple k-mer sizes (e.g. 17-25). The distribution of the resulting values might help to estimate the true genome size.
JellyFish (k=17): 563Mb
findGSE (k=21): 589Mb
GenomeScope (k=21): 489Mb (this is smaller than the actual assembly size)
42) The presented sugar transporters are not among the top enriched GO terms (S2). Therefore, I am afraid that this analysis is not very informative. Could it be that the "enriched" GOs are just a "random" set?

43) Why is E. glaucum not presented as S5C? A direct comparison would make more sense.
44) S10: I would recommend to identify the precise break points. Next, it would be good to validate the accuracy of the assembly by finding individual reads that actually support the situation in E. glaucum. This would help to exclude an assembly artifact as reason for the difference.
45) It might be better to use a three letter abbreviation of the species ("Egl" instead of "Eg") in the gene IDs to avoid ambiguities in future genome sequencing projects.
46) The method section states that short DNA fragments below 12kb were removed. S11 suggests that two libraries were sequences: one with depletion of the short fragments and one without it. Please check this. Generally, I would recommend to try a different gDNA extraction protocol and to use SRE instead of BluePippin.
47) The north of eg06 looks suspicious in the Hi-C analysis (S12). There is also no substantial synteny with any of the Musa chromosomes (S8). Could this be an indication that there are errors in the assembly?
48) Table S1: What is the point in showing that all contigs are larger than 1, 2, and 5kb?
49) 445 bHLHs in M. acuminata is almost twice the number of bHLHs detected in E. glaucum. Some other TF families also show this large difference, but orther families show almost equal numbers. It could be interesting to further investigate this. The HB-KNOX value of M. acuminata is missing.
Minor comments:
line 70/71: Some countries are named multiple times. Please change this.
line 113: chromosomes > pseudochromosomes
line273/274: Please check this sentence.
line 428: Please rephrase "translated proteins" and SynVisio should only be named in the method section.
line 436: "protein-coding genomes" ?
line 464: "second (right)" ... should be replaced by north/south or q/p nomenclature. This also affects some following sentences.
line 625: "Musa acuminata" is a species name
line 639: blast > BLAST
line 731: of of > of
line 811: RNA-sequencing > RNA-seq (I have not seen a section about RNA sequencing)
S10: "E glaucum" > "E. glaucum"

Level of Interest

Please indicate how interesting you found the manuscript: Choose an item.

Quality of Written English

Please indicate the quality of language in the manuscript: Choose an item.

Declaration of Competing Interests

Please complete a declaration of competing interests, considering the following questions:

- Have you in the past five years received reimbursements, fees, funding, or salary from an organisation that may in any way gain or lose financially from the publication of this manuscript, either now or in the future?
- Do you hold any stocks or shares in an organisation that may in any way gain or lose financially from the publication of this manuscript, either now or in the future?
- Do you hold or are you currently applying for any patents relating to the content of the manuscript?
- Have you received reimbursements, fees, funding, or salary from an organization that holds or has applied for patents relating to the content of the manuscript?
- Do you have any other financial competing interests?
- Do you have any non-financial competing interests in relation to this paper?

If you can answer no to all of the above, write 'I declare that I have no competing interests' below. If your reply is yes to any, please give details below.

I declare that I have no competing interests

I agree to the open peer review policy of the journal. I understand that my name will be included on my report to the authors and, if the manuscript is accepted for publication, my named report including any attachments I upload will be posted on the website along with the authors' responses. I agree for my report to be made available under an Open Access Creative Commons CC-BY license (http://creativecommons.org/licenses/by/4.0/). I understand that any comments which I do not wish to be included in my named report can be included as confidential comments to the editors, which will not be published.

Choose an item.

To further support our reviewers, we have joined with Publons, where you can gain additional credit to further highlight your hard work (see: https://publons.com/journal/530/gigascience). On publication of this paper, your review will be automatically added to Publons, you can then choose whether or not to claim your Publons credit. I understand this statement.

Yes Choose an item.
