# Supplementary material for: A chromosome-level reference genome of Ensete glaucum gives insight into diversity and chromosomal and repetitive sequence evolution in the Musaceae
Source: Gigascience. 2022 Apr 30;11:giac027. doi: 10.1093/gigascience/giac027 (PMC9055855; doi:10.1093/gigascience/giac027)
Supplement: giac027_Reviewer_2_Report_Original_Submission — Ning Jiang, Ph. D -- 12/9/2021 Reviewed [file giac027_reviewer_2_report_original_submission.pdf]

Reviewer Report

Title: A chromosome-level reference genome of Ensete glaucum gives insight into diversity, chromosomal and repetitive sequence evolution in the Musaceae

Version: Original Submission **Date:** 12/9/2021

Reviewer name: Ning Jiang, Ph. D

Reviewer Comments to Author:

Comments to the authors
In this study, the authors described the generation of a high-quality reference genome of Ensete glaucum, which is one of the most cold-hardy species in the Musaceae. It is also well known for its drought tolerance. The authors compared the expansion and contraction of gene families and the composition of repeats among related species. The genome assembly, analysis, and annotation are certainly useful for comparative genomic studies as well as future breeding practice. Everything seems to make sense to me. Certainly, the results are descriptive, but this is more than sufficient for a data note.

Level of Interest

Please indicate how interesting you found the manuscript: Choose an item.

Quality of Written English

Please indicate the quality of language in the manuscript: Choose an item.

Declaration of Competing Interests

Please complete a declaration of competing interests, considering the following questions:

- Have you in the past five years received reimbursements, fees, funding, or salary from an organisation that may in any way gain or lose financially from the publication of this manuscript, either now or in the future?
- Do you hold any stocks or shares in an organisation that may in any way gain or lose financially from the publication of this manuscript, either now or in the future?
- Do you hold or are you currently applying for any patents relating to the content of the manuscript?
- Have you received reimbursements, fees, funding, or salary from an organization that holds or has applied for patents relating to the content of the manuscript?
- Do you have any other financial competing interests?

- Do you have any non-financial competing interests in relation to this paper?

If you can answer no to all of the above, write 'I declare that I have no competing interests' below. If your reply is yes to any, please give details below.

I declare that I have no competing interests

I agree to the open peer review policy of the journal. I understand that my name will be included on my report to the authors and, if the manuscript is accepted for publication, my named report including any attachments I upload will be posted on the website along with the authors' responses. I agree for my report to be made available under an Open Access Creative Commons CC-BY license (http://creativecommons.org/licenses/by/4.0/). I understand that any comments which I do not wish to be included in my named report can be included as confidential comments to the editors, which will not be published.

Choose an item.

To further support our reviewers, we have joined with Publons, where you can gain additional credit to further highlight your hard work (see: https://publons.com/journal/530/gigascience). On publication of this paper, your review will be automatically added to Publons, you can then choose whether or not to claim your Publons credit. I understand this statement.

Yes Choose an item.
